# The evolution of extra-pair paternity and paternal care in birds

**DOI:** 10.1093/beheco/arad053

**Published:** 2023-06-23

**Authors:** Jørgen S Søraker, Jonathan Wright, Fredrik Øglænd Hanslin, Michael Le Pepke

**Affiliations:** Centre for Biodiversity Dynamics, Department of Biology, Norwegian University of Science and Technology Høgskoleringen 5, NO-7491 Trondheim, Norway; Department of Natural History, NTNU University Museum, Norwegian University of Science and Technology, Trondheim, Norway; Centre for Biodiversity Dynamics, Department of Biology, Norwegian University of Science and Technology Høgskoleringen 5, NO-7491 Trondheim, Norway; Department of Biology, Norwegian University of Science and Technology Høgskoleringen 5, NO-7491 Trondheim, Norway; Centre for Biodiversity Dynamics, Department of Biology, Norwegian University of Science and Technology Høgskoleringen 5, NO-7491 Trondheim, Norway

**Keywords:** extra-pair paternity, incubation, life history, nestbuilding, parental care, phylogenetic path analysis, provisioning

## Abstract

Extra-pair paternity (EPP) influences the relatedness between social parents and offspring. Therefore, one might expect the level of EPP to influence levels of paternal investment. Here, we investigated the effect of variation in EPP rates on male contributions to parental care within a phylogenetic framework of up to 271 primarily socially monogamous bird species representing 85 families. We used proportion of male provisioning and occurrence of male incubation and nestbuilding as measures of paternal care. We tested the relationship between EPP rates and different components of paternal care while controlling for various life-history traits, namely lifespan, clutch size, and body mass in a phylogenetic path analysis framework. EPP was significantly negatively associated with the occurrence (i.e., whether males participate or not) of male nestbuilding and incubation, but not with the relative amount (proportion) of nestbuilding or incubation performed by the male. Importantly, the proportion of provisioning and biomass delivery by males was clearly negatively associated with EPP. These analyses thus confirm that the effect of EPP on proportion of provisioning visits by males is similar to proportion of biomass delivery, an often assumed but rarely tested assumption. Analysing only Passerine species provided similar results, although only proportion of provisioning was significantly negatively associated with EPP. This study, therefore, provides the most comprehensive support to date of a negative relationship between EPP and paternal care across species. However, a causal relationship between EPP and paternal care cannot necessarily be concluded. We also identify key methodological improvements for future research within the topic.

## Teaser

Birds need to take care of their young. Such parental care can be done by both parents, and include various forms of care like nestbuilding, incubation and provisioning. In species with higher levels of extra-pair paternity, where males other than the social father sire offspring, the level of care provided by the male is expected to be lower. Here we show support for this hypothesis, and confirm previous assumptions.

## INTRODUCTION

Parental investment, defined as any investment from parents that enhances offspring fitness at the cost of investment in the parent’s future offspring, is expected to differ between the sexes depending upon the species’ mating system (Trivers 1972). Extensive research in this area has indeed revealed substantial adaptive variation in both the amount and type of male and female parental care within and among different taxa (Kölliker et al. 2014; Clutton-Brock 1991). The evolution of parental care is strongly influenced by other life-history traits and is associated with high adult death rates (Klug and Bonsall 2010) and with increases in offspring fitness during early life stages (Klug and Bonsall 2014). Theoretical models show that levels of paternal care are expected to vary with the operational sex ratio since it affects the degree of sexual selection and variation in paternity (Kokko and Jennions 2008).

Genetic relatedness is expected to be an important driver in the evolution of male and female parental care (Sheldon 2002). The level of paternity for males may be reduced by extra-pair paternity (EPP), but theoretical models differ in their predictions regarding how paternity should affect the level of paternal care (Sheldon 2002). Male care may not be influenced by levels of paternity if there is synchronous breeding and a lack of potential new partners within the same breeding season, leaving parental care as the only fitness-enhancing activity open to males irrespective of variation in paternity (Smith 1977; Grafen 1980). Likewise, the level of paternity may depend upon the individual quality of the males, resulting in predictable among-individual levels of paternity (Wright 1998). Therefore, no adaptive facultative change in within-individual levels of care is predicted since future breeding opportunities will likely match current ones (Wright 1998). Responses in paternal care can also depend upon the male’s ability to assess any changes in his paternity, which might not always be possible (Mauck et al. 1999). For these reasons, among-individual adaptive paternal care responses to variation in paternity may differ between ecological versus evolutionary timescales, that is, between facultative individual-level responses versus average species-level responses (Whittingham et al. 1992; Westneat and Sherman 1993; Wright 1998; Kokko 1999). Therefore, in most cases, theory suggests that there should not necessarily be an effect of paternity on male parental care within species, but a clear positive relationship is still predicted across species (Westneat and Sherman 1993).

EPP occurs frequently in bird species otherwise regarded as socially monogamous (Brouwer and Griffith 2019). The level of EPP has been shown to vary both within and among bird families and orders (Griffith et al. 2002; Brouwer and Griffith 2019) and to be an important determinant for sex roles on an interspecific level because it changes the average relatedness between offspring and caring individuals (Gonzalez-Voyer et al. 2022).

Increasing amounts of genetic data concerning such variation in father-offspring relatedness across bird species, combined with the various ongoing theoretical developments (Whittingham et al. 1992; Klug et al. 2012), have led to several comparative investigations exploring the relationship between levels of EPP and paternal care across avian taxa (Møller and Birkhead 1993; Schwagmeyer et al. 1999; Møller 2000; Møller and Cuervo 2000; Arnold and Owens 2002). The original comparative study by Møller and Birkhead (1993) involved 52 socially polygamous or monogamous bird species and demonstrated the expected positive relationship between the percentage of male nestling provisioning and paternity, but found no support for the effect of paternity on the proportion of male courtship feeding, incubation, or nestbuilding. However, this study was criticized both for including poor EPP estimates and including polyandrous populations (Dale 1995), and partly involving measures of extra-pair copulations rather than EPP (Dunn and Lifjeld 1994). Re-examinations of similar datasets found no support for the original study’s positive association between the proportion of male provisioning and paternity but were able to show a positive effect of paternity on share of male incubation (Schwagmeyer et al. 1999). However, these results were again challenged by Møller and Cuervo (2000), who subsequently used an updated dataset where they controlled for certain potentially confounding factors and found evidence for an effect of EPP on male provisioning, but not on other aspects of paternal care. Moreover, species, where female reproductive success was highly dependent upon male care, showed lower levels of EPP (Møller 2000), which is consistent with the hypothesis that females relying on male care for reproduction would exhibit decreased levels of EPP (Gowaty 1996). In later years, Crouch and Mason-Gamer (2018) investigated this relationship within a structural equation modeling framework and found support for a negative relationship between share of male provisioning visits and EPP, while other studies found that EPP was negatively associated with male nest building (Lifjeld et al. 2019), incubation (Matysioková and Remeš 2013; Lifjeld et al. 2019), or parental care in general (Remeš et al. 2015).

Various comparative analyses have thus attempted to examine key theoretical predictions concerning the relationship between EPP and paternal care in birds. However, these studies have shown variable results regarding the different components of care and have included methodological shortcomings regarding the scoring of paternal care (Arnold and Owens 2002). For example, scoring relative contribution of feeding behavior into categories (e.g., female provide all care, male provide some care, and male provide all care) does not capture the full variation in the amount of paternal feeding behavior. In addition, there has been little examination of the effects on parental care of EPP relative to other factors that might also directly or indirectly affect levels of paternal care, despite the presence of various potentially confounding factors in the ecology and mating systems of the different species concerned (Wright 1998; Brouwer and Griffith 2019). For example, life-history traits like lifespan have previously been shown to be negatively associated with levels of EPP, presumably due to increased costs of divorce in such systems with beneficially long pair durations (Valcu et al. 2021). Body mass has also been shown to be negatively correlated with EPP (Møller and Birkhead 1994), and dichromatism has been identified as a predictor of the strength of sexual selection and increases in EPP in monogamous species (Møller and Birkhead 1994; Petrie et al. 1998; Valcu et al. 2023). Clutch size can potentially affect the probability of EPP, and has also been hypothesized to affect our ability to accurately detect levels of EPP (Arnold and Owens 2002). While these confounding variables may influence EPP, they can also be affected by EPP. For example, variation in opportunities for EPP may again influence levels of sexual selection and dichromatism (Petrie et al. 1998). Therefore, such confounding factors are important to account for and is one of the aims for the present study.

Covariation between EPP and life-history traits can be due to shared evolutionary history (Liu et al. 2022) and it is, therefore, crucial to use the appropriate statistical methods to disentangle the effects of life-history traits and phylogeny to better understand their effect on any relationship between EPP and paternal care. Parental care may also directly be affected by life-history traits such as lifespa because greater investment in parental care is expected in short-lived species (Klug and Bonsall 2010). Therefore, we need to control for the effect of key life-history traits on EPP and on parental care, respectively, when investigating effects of EPP on parental care. Accounting for the effects of phylogeny is important if parental care has coevolved with EPP and life-history traits (Møller and Birkhead 1993; Møller and Cuervo 2000). Although previous studies have highlighted that various aspects of parental care are likely to evolve in response to levels of EPP (Møller and Cuervo 2000), a negative relationship between EPP and paternal care can also evolve from variation in the social mating system (Bennett and Owens 2002). Therefore, some studies have restricted their scope to only include the order Passeriformes, a variable group of species regarding the level of EPP with mostly biparental care (Bennett and Owens 2002; Lifjeld et al. 2019).

The aim of this study is, therefore, 2-fold: First, we aim to investigate the statistical relationships among the key life-history traits of body mass, maximum lifespan, and clutch size that might affect EPP, and secondly, the effects of all of these on various aspects of male parental care (occurrence of male incubation and nestbuilding, proportion of male provisioning and biomass delivery) in bird species regarded as socially monogamous. All previous investigations have relied upon the assumption that feeding frequency is a reliable proxy for provisioning investment by the two sexes, but this may not necessarily be the case for most bird species that differ in parental loads sizes and prey types delivered to dependent offspring (see Wright et al. 1998; Wright et al. 2010; Pande and Dahanukar 2012). Increasing numbers of studies estimating load sizes (i.e., biomass) of prey delivered by parent birds mean that these data are now available for many of the species concerned (e.g., Pande and Dahanukar 2012; Perlut et al. 2012). We were, therefore, able to additionally test the validity of this earlier assumption and to test not only the association between EPP and male proportion of provisioning but also biomass delivery. We included species that were mainly monogamous but sometimes facultative polygamous to see how they affected the results. However, cooperative breeders were excluded from this study due to the range of mating and social systems involved, which would complicate any predictions regarding levels of EPP and variation in the cooperative effort of males within these social groups (Dunn and Cockburn 1998). Although earlier studies have showed that cooperative breeding has only a small effect on paternal care as it relates to EPP (Lifjeld et al. 2019), certain studies have showed strong intraspecific variation in levels of EPP related to the degree of cooperation between individuals (Du and Lu 2009). Finally, we quantified phylogenetic signals in the different forms of paternal care to determine the influence of phylogeny on the evolution of these traits. An updated dataset with these additional variables and our use of phylogenetic path analyses of the different life-history traits should, therefore, provide a more accurate understanding of the (co)evolution of paternity and paternal care than the studies conducted until now.

## MATERIALS AND METHODS

### Life-history data

We used Dunning (2007) as a source of bird species’ body mass estimates. Clutch sizes were compiled from Cramp (1977) and del Hoyo et al. (1992), and when ranges were given, the midpoint value of the range was used. The Animal Ageing and Longevity Database (Tacutu et al. 2018) was used to obtain maximum lifespan from wild populations. We classified maximum lifespan estimates into four categories based on sample size estimates obtained from the Animal Ageing and Longevity Database: tiny <10; small 10–100; medium 100–1000; and large: >1000. We observed a small but significant increase in maximum lifespan with sample size. We, therefore, estimated maximum lifespan corrected for sample size by mean-centering each species’ lifespan value on the mean value within its respective sample size category. There was, therefore, no effect of sample size on corrected maximum lifespan estimates (Supplementary Figure S1). We then reran all analyses (see below) using the corrected maximum lifespan estimates. All results remained qualitatively the same after correcting maximum lifespan for sample size (Supplementary Figure S2), and since sample sizes were reduced using this approach due to some species missing lifespan sample size estimates, we present the main analyses without this correction.

### Paternity and paternal care data

We mainly used Brouwer and Griffith (2019) as a source for rates of EPP among birds (254 species). We performed a systematic literature search for additional studies on EPP in birds using Google Scholar and the keywords “extra-pair paternity” and “bird,” which contributed 17 species to the dataset. The EPP estimates in Brouwer and Griffith (2019) were updated if new publications with non-overlapping datasets were found. Percentage of offspring sired by extra-pair males was used as a measure of EPP. We restricted our study to only include primarily socially monogamous species (i.e., species that form social pairs and have at least some biparental care) of birds (*n* = 271), and this also included monogamous species that sometimes show facultative polygamous to varying degrees (*n* = 25) A separate analysis was carried out excluding those species with occasional polygamous behavior (*n* = 246) in order to confirm that the results were qualitatively unaffected by their inclusion. The models excluding species with occasionally polygamous behavior yielded similar results with respect to the role of EPP on parental care components (Supplementary Figure S3). Therefore, we present a new, updated dataset for estimates of EPP across bird species (Supplementary Table S1 and Supplementary Figure S4).

We used six different measures of paternal care. For nestbuilding and incubation, we used two separate measures. First, we used the occurrence of male participation in these behaviors. That is a binary variable reflecting whether or not the male participates in at least some nestbuilding or incubation. Second, we used the quantified proportion of these behaviors performed by the male, measured as the proportion of the total nest built by the male or the proportion of the total time of incubation performed by the male. We used the proportion (percentage of total amount) for both provisioning (food delivery events to offspring) and biomass delivery (the biomass of food delivered to offspring) as a quantification of these behaviors, hereafter referred to as proportion of provisioning and proportion of biomass delivery, respectively.

Cramp (1977) and del Hoyo et al. (1992) and Birds of the World (birdsoftheworld.org) were used to extract information on the occurrence of male nestbuilding and incubation behavior. When these sources did not contain the data needed, we carried out species-specific searches on Google Scholar for articles containing the information. In total, data for 63 species were extracted from species-specific sources, either as the sole source, or as an additional source backing up other sources (see Supplementary Table S1 for details). Although several attempts have been made to quantify male contributions with regard to the actual proportion of nestbuilding and incubation, rough estimates of paternal effort relative to an approximately equal contribution between sexes have often had to be used instead (Møller and Birkhead 1993; Møller and Cuervo 2000). We, therefore, collected two different datasets for both nestbuilding and incubation behavior. First, the more conservative approach of expressing male occurrence of parental care activities as binary (yes/no) variables (*n* = 181 and *n* = 246 species on nestbuilding and incubation, respectively). Second, the reported proportions of these behaviors by males to investigate the same relationships on a proportional scale (*n* = 109 and *n* = 186 species on nestbuilding and incubation). Species with no nestbuilding by either sex were not included in the analyses assessing effects on nestbuilding as measure of paternal care.

Several previous studies have relied upon unpublished estimates of the proportion of male nestling provisioning obtained via personal communications (see Møller and Birkhead 1993; Schwagmeyer et al. 1999; Møller and Cuervo 2000), thus making the data collation process less reliable and transparent. We, therefore, performed a species-specific systematic search of the primary literature, using Google Scholar with “provisioning,” “feeding,” and “food biomass” as keywords in November 2021 for reports of the proportion of male provisioning and biomass delivery. We also split the dataset into two parts: 1) only studies that quantified and reported proportion of provisioning for male and females (*n* = 101 species) from which we obtained a proportion of total provisioning carried out by males; and 2) also including additional studies that only reported sex differences in provisioning without providing any values (*n* = 5 additional species), for example stating that the sexes shared provisioning equally (corresponding to a male provisioning proportion of 0.5). Although these two datasets should provide similar results, we analyzed them separately in order to test this assumption, resulting in one test with 101 species (only quantitative estimates of provisioning proportions, Supplementary Table S2), and one test with 106 species (also including simple statements of sex differences in provisioning, Supplementary Table S3). When provisioning data were available for different years, we used the mean of the reported values, except when sample sizes were given, in which case the value was the weighted mean across years. When more than one article or data source was available for the same population with overlapping years, the one with the largest sample size was used. The same procedures were also used for the proportion of total biomass delivery by males. Species data were available for nestbuilding (occurrence, *n* = 185; proportion, *n* = 111), incubation (occurrence, *n* = 251; proportion, *n* = 190), provisioning (proportion, *n* = 106), and biomass delivery (proportion, *n* = 29). Studies that reported numerical values were used to calculate perhaps more reliable proportional data for nestbuilding (*n* = 111), incubation (*n* = 182), provisioning (*n* = 101), and biomass delivery (*n* = 28). Because covariation between EPP and paternal care could also result from variation in social mating systems and the opportunity of desertion, and further that Passerines show extraordinary diversity in EPP (Arnold and Owens 2002), we also constructed models including only Passerine bird species with data for nestbuilding (occurrence, *n* = 141; proportion, *n* = 99), incubation (occurrence, *n* = 142; proportion, *n* = 128), proportion of provisioning (*n* = 72) and proportion of biomass delivery (*n* = 15). This analysis would also allow for comparisons with studies only including Passerines.

### Phylogenetic reconstruction

We compiled a set of 1000 time-calibrated bird phylogenetic trees (Jetz et al. 2012) based upon the Hackett et al. (2008) backbone from BirdTree.org, and we summarized these into a single maximum clade credibility tree using the maxCladeCred function in the “phangorn” package in R (Schliep 2011). The tree was pruned to match the species with EPP information (*n* = 271) using “ape” (Paradis and Schliep 2019). For two species missing from the phylogeny (*Ardea alba* and *Charadrius nivosus*) we used the phylogenetic placement of their closely related congeners (*Ardea herodias* and *Charadrius peronii*, respectively). Phylogenies and ancestral states were estimated and visualized using the “fastAnc” function in the “phytools” package (Revell 2012). Phylogenetic signals were estimated for trait associations and for each trait separately as Pagel’s *λ* (Pagel 1999) optimized numerically within the default bounds 0.00–1.00 using maximum likelihood in the “caper” package (Orme et al. 2013).

### Phylogenetic path analyses

We used phylogenetic generalized least-squares regressions (Grafen 1989) to perform phylogenetic path analysis (Hardenberg and Gonzalez-Voyer 2013) to investigate the effects of EPP and life-history traits on paternal care components. We performed six separate analyses of each of the paternal care response variables (occurrence of nestbuilding, proportion nestbuilding, occurrence of incubation, proportion incubation, proportion provisioning,, and proportion biomass delivery) being potentially affected by EPP, maximum lifespan, body mass, and clutch size. Furthermore, we included the path effects of maximum lifespan, body mass, and clutch size on EPP, respectively, in all models. By simultaneously accounting for the influences of the different life-history traits on both EPP and paternal care, we were, therefore, able to more appropriately test the separate association between EPP and parental care. We calculated Pearson’s phylogenetic correlation coefficients for all predictors using the phyl.vcv function in *phytools* to compute the phylogenetic trait variance-covariance matrix and then using a joint optimization of the *λ* value for each trait pair using the likMlambda function (Revell 2012, Supplementary Table S4). The path analyses were performed using the *phylopath* package (van der Bijl 2018), which implements linear phylogenetic regressions (for proportions of provisioning and biomass delivery) and logistic phylogenetic regressions (for occurrence of male nestbuilding and incubation) of the *phylolm* package (Tung Ho and Ané 2014) using Pagel’s lambda model of trait evolution. Furthermore, we used phylogenetic least square regression to test for an association between proportion of male provisioning and biomass delivery. We report standardized regression coefficients, their standard errors, and 95% confidence intervals (CI), which were calculated by taking 500 bootstrap replicates. All statistical analyses were performed in R v. 4.2 (R Core Team Rf 2018).

## RESULTS

We obtained EPP estimates for 271 bird species, with species level estimates ranging from 0.0% to 65.2%, and EPP was detected in 77% of the species (Supplementary Figure S4). Phylogenetic signals were strong in all paternal care components (*λ* ranging from 0.68 to 1.00, see Supplementary Table S4) and in all life-history traits (*λ* ranging from 0.78 to 1.00, Supplementary Table S4), and there was a relatively high phylogenetic signal in EPP (*λ* = 0.56, Supplementary Figure S4). Although the life-history traits were generally correlated, none were highly correlated (Supplementary Table S5).

### Associations between EPP and paternal care

**Figure 1:**
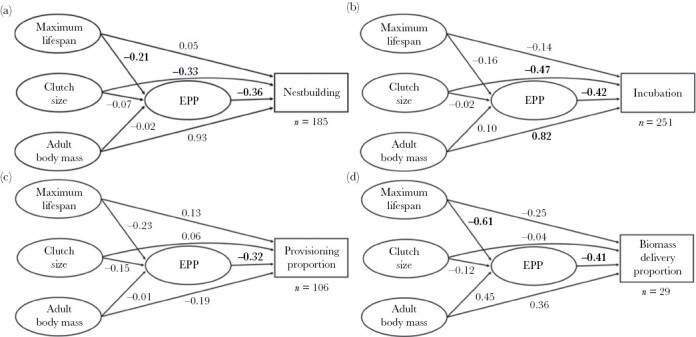
Phylogenetic path diagrams conceptualizing the relationships between key life-history traits, EPP for the four different measurements of paternal care: (a) occurrence of male nestbuilding; (b) occurrence of male incubation; (c) proportion of male provisioning; and (d) proportion of male biomass delivery. Values on each path arrow show phylogenetic regression coefficients, with bold values indicating statistically significant associations (*P* < 0.05). Sample size (*n*) for each analysis is also shown under the paternal care measure box.

**Figure 2:**
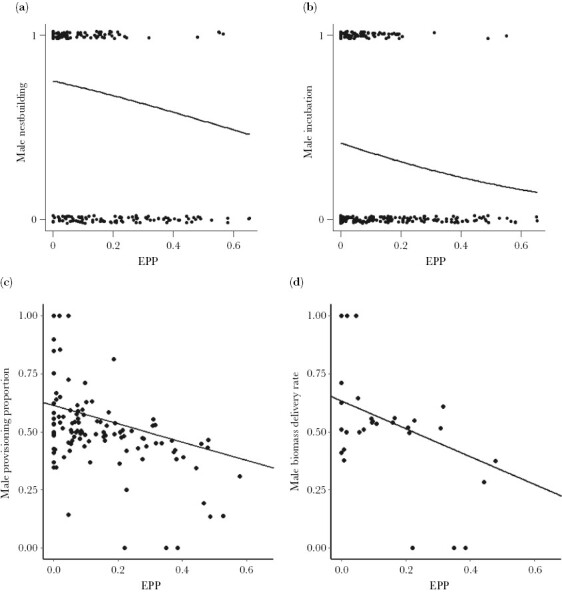
Relationships between the four different measurements of parental care and EPP. Each datapoint represent a species and the phylogenetic regression lines are shown.

Occurrence of male nestbuilding (as binomial trait) was negatively associated with EPP (*β*_EPP_ = −0.357, CI = [−0.665, −0.069], *n* = 185, Supplementary Table S6, Figures 1 and 2, and Supplementary Figure S5). Neither lifespan, clutch size nor adult body mass affected EPP (Supplementary Table S6). When treated as a proportional trait, we found a negative but statistically nonsignificant effect of EPP on proportion of nestbuilding (Supplementary Table S7 and Supplementary Figure S6). However, this model was biased towards species with no male nestbuilding, since all estimates of no participation were still included while quantified, non-zero estimates were rare. The model violated assumptions of the residual distribution, and although such models may be robust to such violations (Schielzeth et al. 2020), we need to be careful here with any interpretation of this result, and they are therefore placed in the supplementary materials. A binary model treating only Passerine species showed similar trends and effect sizes, but these were marginally significant (*β*_EPP_ = −0.293, CI = [−0.663, 0.021], *n* = 141, Supplementary Table S8), probably because this model contained 44 species less than the one involving all species. A proportional model with Passerine species also showed all the same trends as the proportional model involving all species but again showed somewhat nonsignificant results (Supplementary Table S9 and Supplementary Figure S7).

The path analyses treating occurrence of incubation (as binomial trait) as a measure of parental care revealed that EPP was negatively associated with occurrence of male incubation (*β*_EPP_ = −0.419, CI = [−0.967, −0.040], *n* = 251, Supplementary Table S10, Figures 1 and 2, and Supplementary Figure S5). Occurrence of male incubation was also less common for species with larger clutch sizes and males participated more often in incubation in species with larger body sizes. Neither lifespan, body size nor clutch size directly affected EPP in this analysis (Supplementary Table S10). The model including proportion of incubation (*n* = 190) showed a nonsignificant negative effect of EPP (Supplementary Tables S11 and S12), but it suffered from the same statistical complications as the model involving proportional nestbuilding (see above) and as a precautionary measure it has also been placed in the supplementary materials. The binary model including only Passerine species provided a marginally significant result with similar effect size as the model with all species (*β*_EPP_ = −0.599, CI = [−1.204, 0.000], *n* = 142, Supplementary Table S13). The proportional model with Passerine species (*n* = 128) also showed a nonsignificant negative effect (Supplementary Table S14 and Supplementary Figure S7), but also suffered the same statistical complication as the proportional model for all species and is placed in the Supplementary Materials.

**Figure 3:**
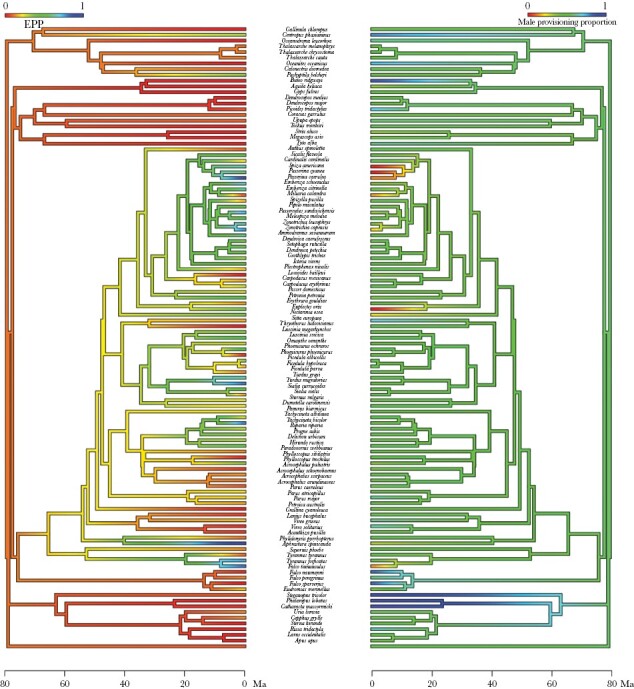
The evolution of EPP and proportion of male provisioning for 108 species, with both male provisioning proportion and EPP estimates color-coded. Both scales ranges from 0% (red) to 100% (dark blue), and EPP ranges from 0% to 65%, while proportion of male provisioning ranges from 0% to 100%. The timescale is in million years ago (Ma).

Proportion of male provisioning was negatively associated with EPP when controlling for the effects of life-history traits (*β*_EPP_ = −0.321, CI = [−0.525, −0.135], *n* = 106, Supplementary Table S3, Figures 1–3). Therefore, the proportion of male provisioning visits decreased as a result of higher EPP rates across these bird species. None of the life history traits (maximum lifespan, clutch size, or adult body mass) affected neither EPP nor the proportion of male provisioning significantly. The model also including proportion of male provisioning visits solely based upon reported values produced similar results (*β*_EPP_ = −0.320, CI = [−0.516, −0.132], *n* = 101, Supplementary Table S2), and so did the model including only Passerine species (*β*_EPP_ = −0.408, CI = [−0.602, −0.190], *n* = 72, Supplementary Table S15 and Supplementary Figure S7).

The last analyses here contained only 29 species but revealed the same negative relationship between EPP and proportion of biomass delivered by males (*β*_EPP_ = −0.412, CI = [−0.691, −0.069], *n* = 29, Supplementary Table S16 and Figures 1 and 2). Neither body mass nor clutch size affected EPP nor biomass proportion, while maximum lifespan was positively associated with EPP (Supplementary Table S16 and Figure 1). The model including only proportion male biomass delivery based on reported values produced similar results (*β*_EPP_ = −0.409, CI = [−0.708, −0.108], *n* = 28, Supplementary Table S17). A model including only Passerine species resulted in a nonsignificant (95% CI only just overlapped zero) with similar effect size (*β*_EPP_ = −0.395, CI = [−0.806, 0.063], *n* = 15, Supplementary Table S18 and Figure S7). A phylogenetic generalized least square regression of the effect of the proportion of male provisioning on male biomass delivery showed a strong positive relationship (*β* = 0.948, *P* < 0.001) between these two measures of paternal food provisioning (Supplementary Figure S8).

## DISCUSSION

This study conclusively demonstrates that the level of paternal care in socially monogamous birds is positively related to paternity, which is generally in-line with previous investigations on this topic (Møller and Birkhead 1993; Schwagmeyer et al. 1999; Møller and Cuervo 2000). However, in contrast to previous studies, we show that PP affects both the probability of occurrence of male pre-hatching care (nestbuilding and incubation) and the magnitude of post-hatching paternal care (proportions of male provisioning visits as well as biomass delivery). Moreover, the relatively high phylogenetic signals in both EPP and paternal care parameters suggest that these traits have been relatively phylogenetically conserved across the evolution of birds (e.g., Kamilar and Cooper 2013). Current theory suggests that an effect of EPP on paternal care might operate only on evolutionary timescales (Westneat and Sherman 1993; Wright 1998; Kokko 1999), rather than within-individual lifetimes (Sheldon 2002). However, the causal dynamics of this relationship remains unclear.

We found that occurrence of male nestbuilding as a binomial measure was negatively associated with EPP. However, when using the more problematical proportional scale estimates for male nestbuilding, our results suggest no proportional decrease in nestbuilding to levels of EPP. This latter result could be seen to confirm previous investigations, which found no support for such a relationship (Møller and Birkhead 1993; Møller and Cuervo 2000). However, a larger data set using our more conservative binomial measure shows a clear reduction in the probability of occurrence of male nestbuilding, which has not previously been demonstrated probably due to our larger sample size and more robust statistical models. So, we have reason to doubt the nonsignificant results here concerning the proportion of male nestbuilding due to the poor quality and bias in these data. It is possible that these inconsistent results are the result of a threshold-like effect, where the male nestbuilding effort only occurs at a sufficiently high level of EPP and then does not increase after this, rather than exhibiting the predicted continuous adjustment of care. However, this possibility requires theoretical justification and further empirical investigation.

The binomial models also showed that EPP was negatively related to occurrence of male incubation. Again, this was not found on the proportional scale, although this result should again be treated with caution (see “Methods” section). A lack of a proportional response in male incubation to EPP is again consistent with some earlier investigations (Møller and Birkhead 1993; Møller and Cuervo 2000), but challenges the findings of both older and newer studies (Schwagmeyer et al. 1999; Matysioková and Remeš 2013). Our new proportional incubation effort analysis included considerably more species than these previous studies (186 species versus 22 and 72 species, respectively). Furthermore, one of these previous studies (Matysioková and Remeš 2013) did not control for variation in life-history traits, and they excluded species with no occurrence of male incubation, as they were specifically interested in incubation behavior and not in other forms of male parental care. They also included only Passerine bird species, where males rarely participate in incubation, and, therefore, were perhaps more likely to find an effect of EPP. However, when restricting our analysis to Passerines, our model showed a negative relationship with CIs only just overlapping zero, indicating that the size of the dataset may be important for clear results in this case. Given the results of our two models on male contributions to incubation, it is also possible that there is a biological threshold effect on occurrence of male incubation, but again this requires further investigation, and it seems more likely that our results reflect differences in the quality of the datasets and robustness of the statistical models.

Incubation contributions to parental care are interesting because this behavior is not only time consuming but also timing constrained, since the male cannot leave before the female returns without adversely affecting offspring development (Ketterson and Nolan 1994). This potentially constrains male behavior during specific periods when they could be potentially spending time seeking extra-pair mates, and it also restricts the potential for male mate guarding at least early on in incubation (Ketterson and Nolan 1994; Schwagmeyer et al. 1999). Therefore, the lack of a significant response to EPP in the proportion of male incubation may be due to this reproductive trade-off for males in species with no male incubation (Westneat et al. 1990), although this form of male parental care does not exclude any opportunities for additional mating (Magrath and Komdeur 2003). However, our path model showed a clear negative effect of EPP on the probability of occurrence of male incubation, plus a positive effect of adult body mass on whether males incubate or not. Although not explicitly tested here, this does suggest that larger species may have a slower pace-of-life and lower EPP, which is also supported by the covariation of body mass and lifespan (Arnold and Owens 2002). Interestingly, however, adult body mass was not related in this way to any other measure of paternal care. In this model, mean clutch size was also negatively associated with male incubation. Hence, our analysis shows that larger bird species with smaller clutch sizes (i.e., a slow pace-of-life) are expected to more often show paternal incubation, as compared with smaller species with large clutch sizes.

Proportion of provisioning by males was found to be negatively affected by species-level EPP rates when controlling for the effects of several life-history traits. This is in-line with several previous studies (Møller and Birkhead 1993; Møller and Cuervo 2000), but not with Schwagmeyer et al. (1999). Again, our study contains considerably more species with male provisioning and EPP data, and this probably explains the difference in results with this study. Our results also strongly challenge recent findings suggesting a negative relationship between male care and EPP only in pre-hatching parental behaviors, although the study in question did not use a proportional scale of male parental care contributions (Lifjeld et al. 2019). Provisioning is likely the most energetically costly form of care, and should, therefore, provide the best evidence for any effect of EPP on male parental care, because selection on responses to EPP is expected to be greatest on more costly behaviors (Trivers 1972; Sheldon 2002).

Lastly, our results support the hypothesized negative relationship between EPP and the proportion of male biomass delivery. This is an important finding, as previous investigations focused solely on the effect on male provisioning visits (Møller and Birkhead 1993; Møller and Cuervo 2000), and thus rely upon the assumption that the proportion of male provisioning is a reliable proxy for the proportion of male biomass delivery. This does not necessarily have to be true, since species and individuals can differ markedly in their load sizes and prey types delivered per visit (Wright 1998; Wright et al. 2010; Pande and Dahanukar 2012). Nevertheless, we can confirm the previously assumed but empirically weakly supported relationship between these two measures of parental care, which is consistent with the findings of an effect of EPP on the proportion of male provisioning and biomass.

In two of the models (nestbuilding and biomass delivery), maximum lifespan was negatively associated with levels of EPP. This agrees with the state-dynamic models of Mauck et al. (1999) arguing that a shorter lifespan reduces the number of possible lifetime reproductive events, and so increases the importance of male investment in EPPs in the few breeding seasons and reproductive attempts available to short-lived species. Maximum lifespan also tended to be negatively associated with EPP in the model with binary incubation behavior and supported when proportion incubation was used, which suggests that maximum lifespan represents the effect of the average number of reproductive events per male lifespan on the species EPP rate.

Although our results suggest that EPP may have coevolved with the different parameters of paternal care, there are alternative explanations for such a negative relationship. In the species showing high levels of EPP, males may have evolved to increase their investment in extra-pair copulations, and, therefore, reduced any paternal care as a byproduct of a reproductive trade-off (Westneat et al. 1990). Under this scenario, males would gain a fitness benefit from potentially increasing their number of offspring, although it may affect their social offspring negatively (Westneat et al. 1990). The negative relationship between EPP and paternal care can also occur in the absence of co-evolution between the two traits. For example, variation in life-history traits among species, such as short durations of periods of care, can increase the risk of males deserting their offspring (Bennett and Owens 2002). This in combination with ecological factors like high breeding densities can again make it beneficial for males to desert their offspring to instead seek extra-pair copulations. In this case, the negative relationship between EPP and paternal care would be driven by evolutionary and ecologically mediated variation in mating systems, rather than a direct adaptive reduction in the level of male care in response to EPP (Bennett and Owens 2002). This issue has often been partly solved by restricting the scope to only include Passerine species due to lower desertion rates (Bennett and Owens 2002; Lifjeld et al. 2019), and so when structuring our models with only Passerines, we obtain similar effect sizes. The effects in these models were marginally significant, probably due to the lower sample sizes compared with the models that included data from all bird species. This indicates that variation within Passerines tends to follow the same relationships as those seen across all bird species, but we still cannot necessarily conclude that EPP is a causal driver of variation in levels of paternal care. Moreover, when restricting our analysis to strictly monogamous species, we obtained similar results as our main model, where the propensity for male desertion should be lower. Further, we only studied four different parameters of parental care, although other aspects of paternal care also exists, like incubation feeding, territory defence, and protection of fledged individuals (Møller and Cuervo 2000).

Although there has been an increased empirical effort to obtain data on both EPP and male parental care contributions in birds, there are still a few shortcomings that make any meta-analysis difficult. Published studies often provide different measures of their sample sizes in terms of the total number of provisioning visits and/or the number of different pairs observed. They rarely provide full information on sample sizes at all levels within and between pairs, years, and populations, which makes it problematic to correctly statistically weight the data from different studies or different parts of the same study in meta-analyses. Incomplete reporting also constrains studies like ours to use proportions of male care in terms of the relative differences between male and female levels of care, rather than being able to explore variation in both the absolute male and female levels of parental effort within a more complete analysis (Wright 1998). Future studies should provide complete information on both sample sizes and the absolute measures of care obtained. Recent open science initiatives requiring the free online availability of all published data sets is clearly the way forward in this regard and promises much for future meta-analyses and comparative investigations of mating systems and life-history variation in birds and other taxa building on the data provided by this study.

In conclusion, we can confirm strong support for a negative relationship between EPP and several measures of paternal care in birds. This represents the most comprehensive study of this relationship so far, and it justifies a core assumption regarding the use of proportion of male provisioning as a proxy for proportion of male biomass delivery. We also show that it is important in such analyses to take into account various aspects of species life histories, because they can affect both EPP and parental care effort in interesting and confounding ways.

## Supplementary Material

arad053_suppl_Supplementary_Figures_S1-7_Tables_S2-18

arad053_suppl_Supplementary_Table_S1

arad053_suppl_Supplementary_Table_S19

arad053_suppl_Supplementary_Table_S20

## Data Availability

Analyses reported in this article can be reproduced using the data provided by Søraker et al. (2023). The full dataset is also given in the Supplementary Table S1 (EPP and parental care data) and Supplementary Table S19 (life history traits) and Supplementary Table S20 (proportional nestbuilding and incubation behavior).
