# Supplementary material for: The evolution of extra-pair paternity and paternal care in birds
Source: Behav Ecol. 2023 Jun 23;34(5):780–9. doi: 10.1093/beheco/arad053 (PMC10516673; doi:10.1093/beheco/arad053)
Supplement: arad053_suppl_Supplementary_Figures_S1-7_Tables_S2-18 [file arad053_suppl_supplementary_figures_s1-7_tables_s2-18.docx]

**Supplementary materials**


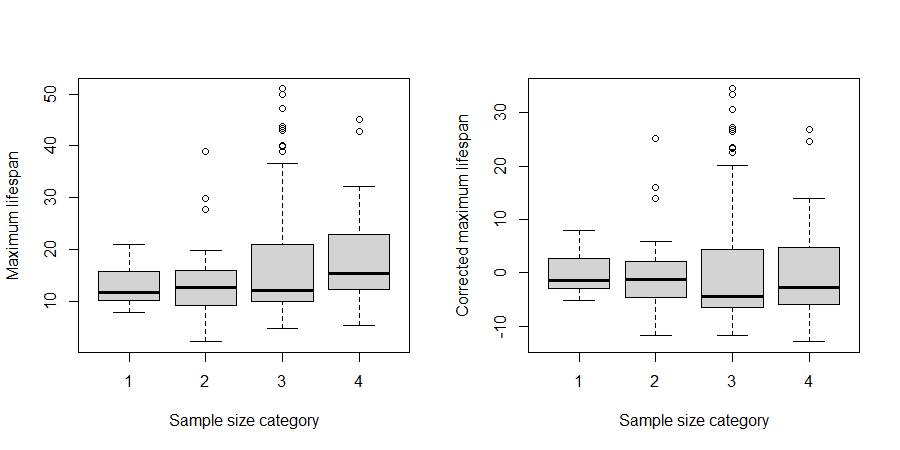


**Figure S1**: The effect of sampling category on lifespan before and after correction by category mean-centering. The sampling categories are determined by sample size; 1) tiny: <10, 2) small: 10-100, 3) medium: 100-1000, 4) large: >1000.


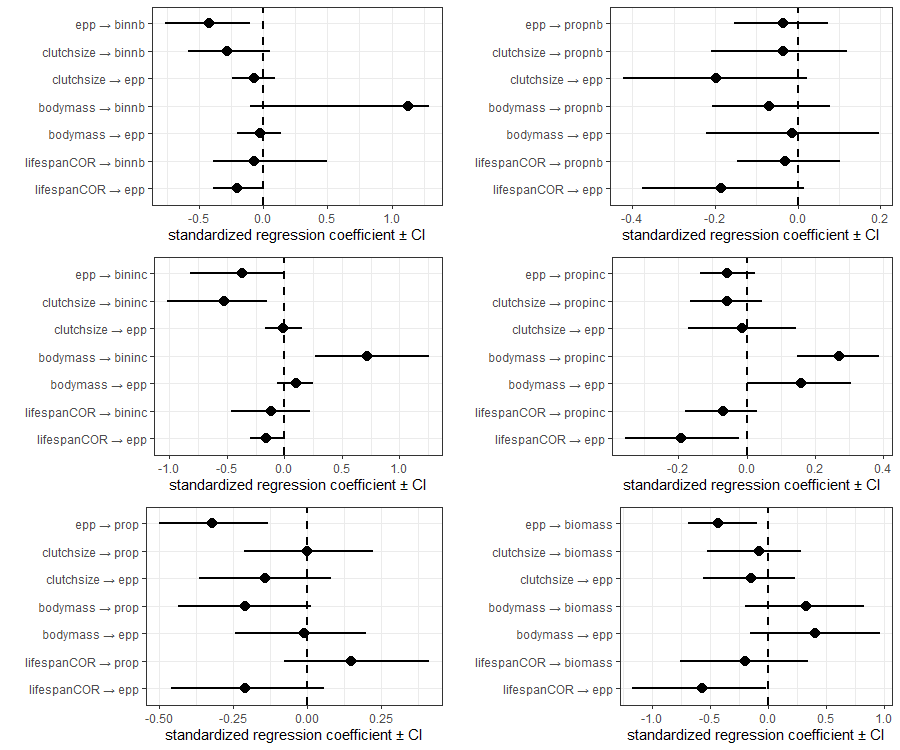


**Figure S2:** Direct phylogenetic path regression coefficients (±95% CI) between maximum lifespan corrected for sample size (lifespanCOR), clutch size (clutchsize) and adult body mass (bodymass) on EPP (epp) and between these four parameters and nestbuilding participation (binnb), proportion of nestbuilding (propnb), incubation participation (bininc), proportion of incubation (propinc) male provisioning rate (prop), and male biomass delivery rate (biomass). Sample size correction was based on sampling categories (Figure S1).

Included in the original analysis are species with occasionally polygyny. Therefore, an analysis excluding those 25 species were performed. The excluded species were *Acrocephalus arundinaceus, Acrocephalus bistrigiceps, Agelaius phoeniceus, Calamospiza melanocorys, Carpodacus erythrinus, Charadrius alexandrines, Charadrius morinellus, Charadrius nivosus, Cistothorus platensis, Dolichonyx oryzivorus, Euplectes orix, Lagopus lagopus, Lagopus leucura, Miliaria calandra, Notiomystis cincta, Passerculus sandwichensis, Petronia petronia, Phalaropus lobatus, Phylloscopus sibilatrix, Phylloscopus trochilus, Picoides tridactylus, Sicalis flaveola, Spiza americana, Steganopus tricolor* and *Troglodytes troglodytes.*


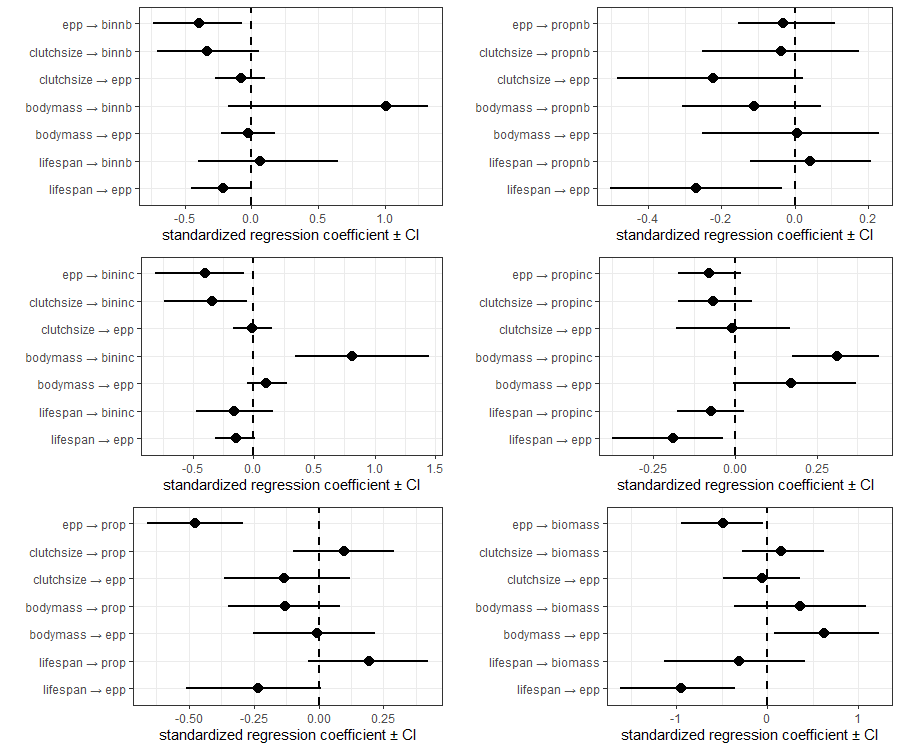


**Figure S3**: Direct phylogenetic path regression coefficients (±95% CI) between maximum lifespan (lifespan), clutch size (clutchsize) and adult body mass (bodymass) on EPP (epp) and between these four parameters and nestbuilding participation (binnb), proportion of nestbuilding (propnb), incubation participation (bininc), proportion of incubation (propinc) male provisioning rate (prop), and male biomass delivery rate (biomass) when excluding species with occasionally polygyny.


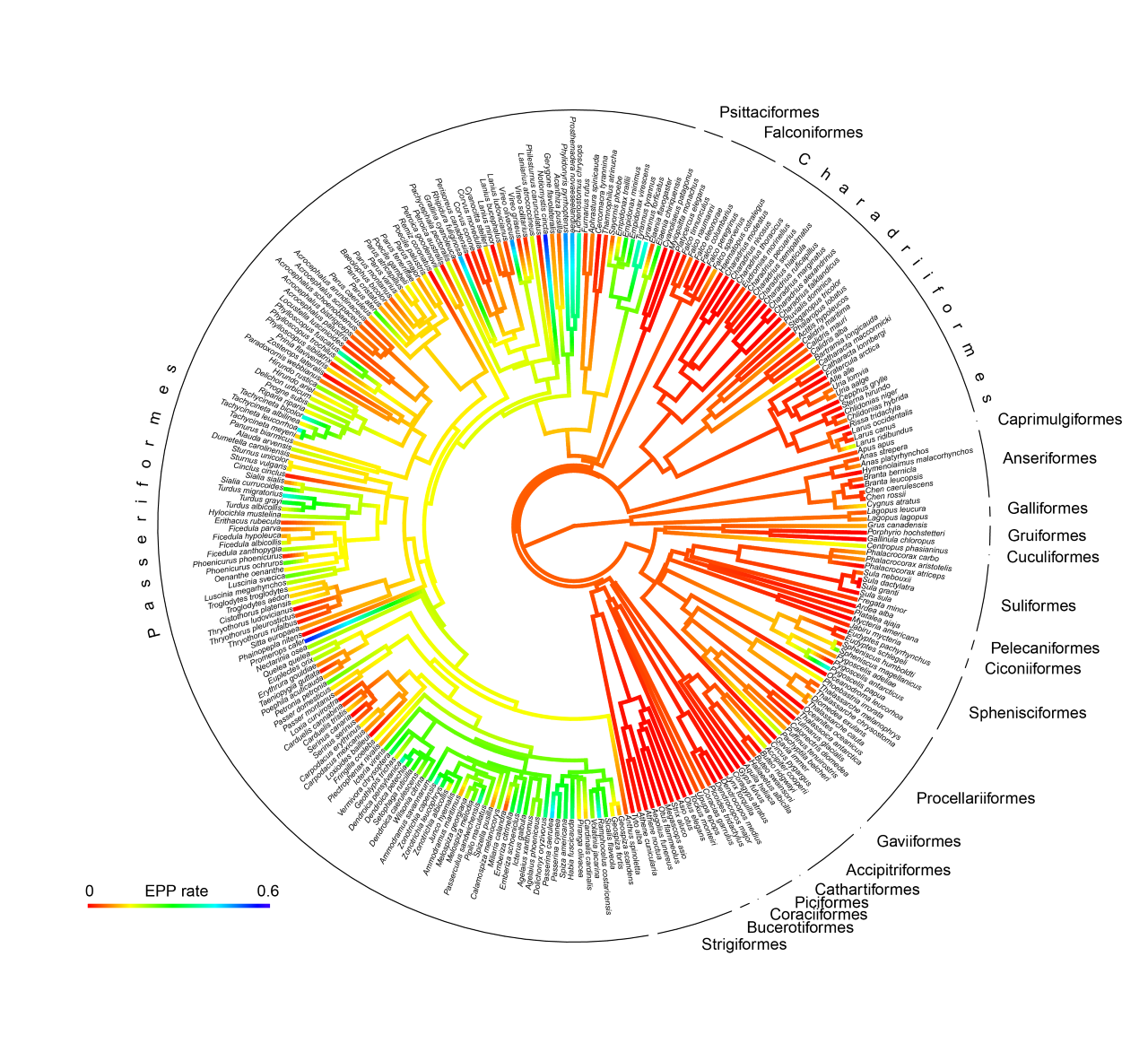


**Figure S4:** The evolution of extra-pair paternity (EPP) among 271 socially monogamous bird species. EPP rates range from 0% (dark red) to 65% (dark blue). The largest diversity in EPP rates was found within the passerines (157 species corresponding to 57.9% of the species), whereas all other more basal clades had low and less variable EPP rates. The ancestral EPP rate of the most recent common ancestor (originating 97.9 million years ago) of all bird species included here was estimated to be 11%, but with a very wide confidence interval (CI = [-48.8, 71.5]).


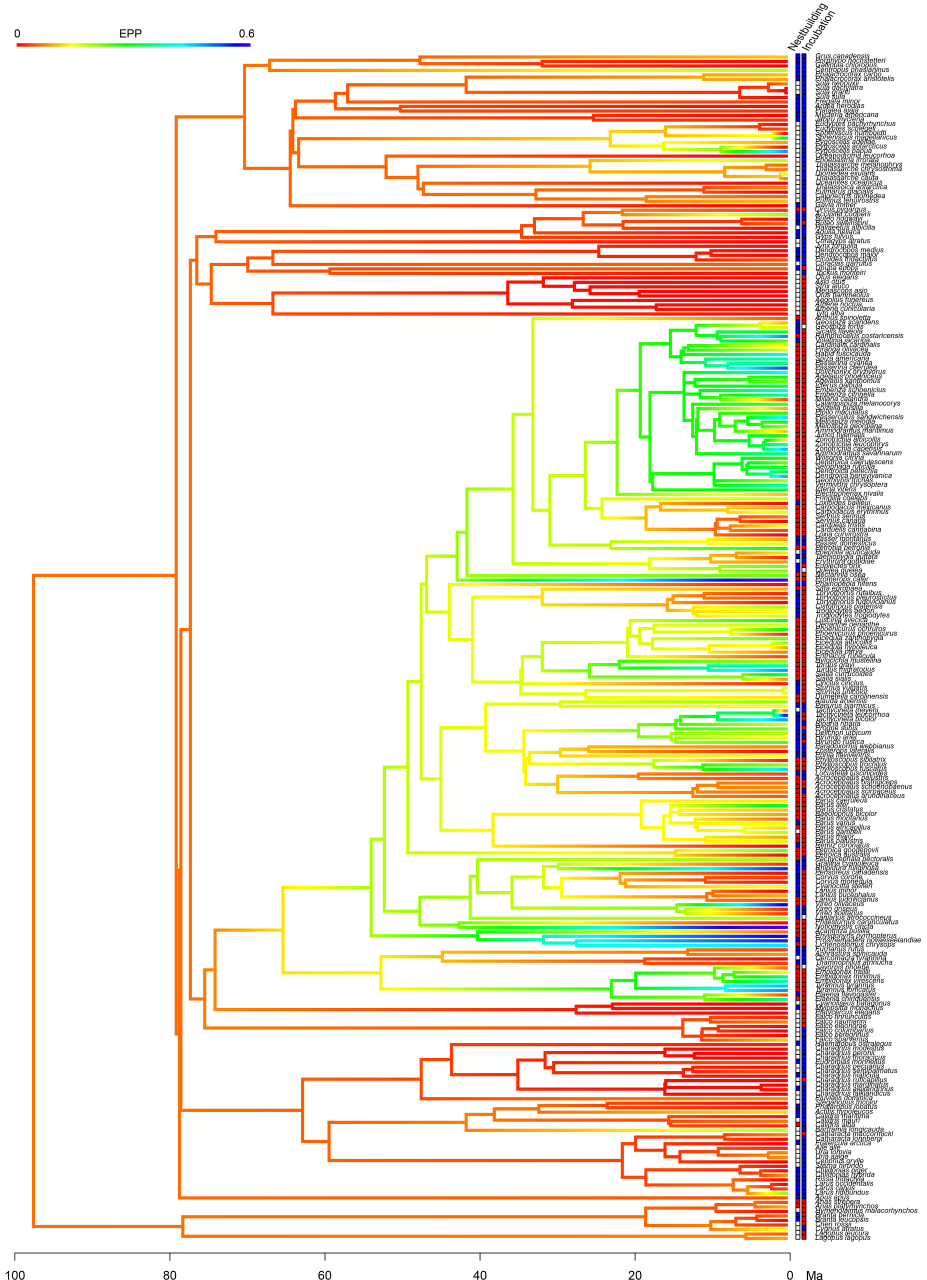


**Figure S5:** The rate EPP among 271 bird species indicating male participation in nestbuilding and incubation as a binary trait. White color indicates missing data, blue indicate male participation and red no male participation.


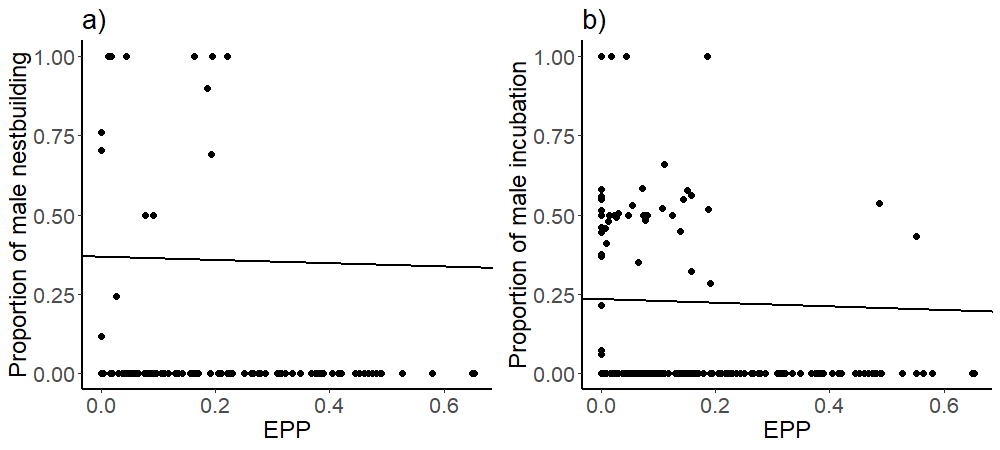


**Figure S6**: Proportion of male contributions to nestbuilding and incubation in relation to extra-pair paternity (EPP). Each datapoint represents a species, the value 0 represent no male involvement and 1 represents only male participation.


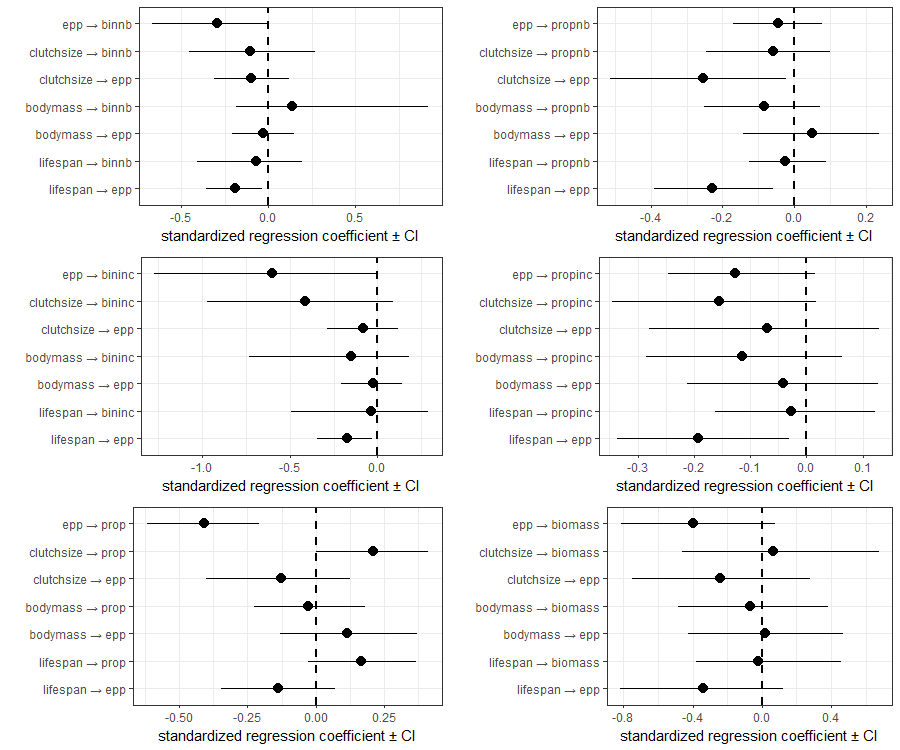


**Figure S7**: Direct phylogenetic path regression coefficients (±95% CI) between maximum lifespan (lifespan), clutch size (clutchsize) and adult body mass (bodymass) on EPP (epp) and between these four parameters and occurrence of male nestbuilding (binnb), proportion of nestbuilding (propnb), occurrence of male incubation (bininc), proportion of incubation (propinc) male provisioning rate (prop), and male biomass delivery rate (biomass) for only Passerine species.


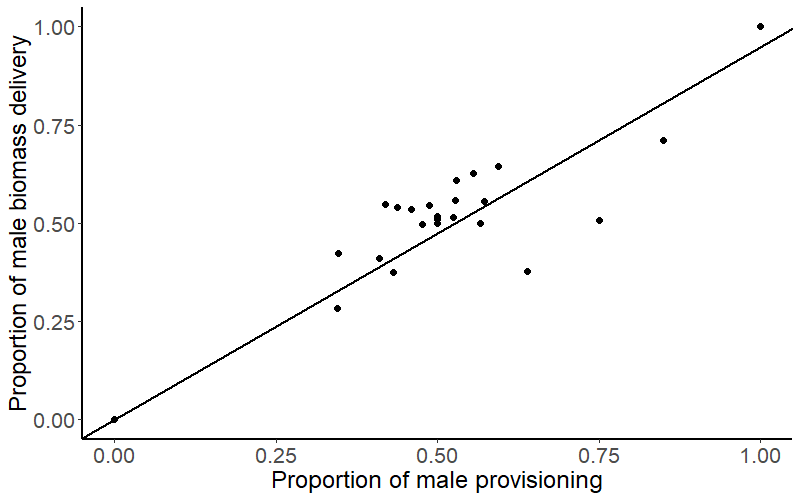


**Figure S8:** The relationship between the proportion of male provisioning visits and the proportion of male biomass delivery. Each point represents a species (n = 28), and the regression is based upon phylogenetic generalized least square regression.

**Table S2:** Effect of life history traits on EPP and effects of life history traits and EPP on male provisioning rate based on 101 species. Included are only studies which provided provisioning values for males and females. Phylogenetic path coefficients (estimate), their standard error (SE) and 95% confidence intervals (CI) are shown. Significant results are marked in bold.

|  | EPP |  |  | Provisioning rate | | |
| --- | --- | --- | --- | --- | --- | --- |
|  | Estimate | SE | CI | Estimate | SE | CI |
| Maximum lifespan | -0.230 | 0.123 | -0.472, 0.012 | 0.121 | 0.125 | -0.140, 0.346 |
| Clutch size | -0.149 | 0.110 | -0.363, 0.080 | 0.041 | 0.111 | -0.161, 0.253 |
| Body mass | 0.005 | 0.112 | -0.207, 0.240 | -0.188 | 0.112 | -0.396, 0.031 |
| EPP | NA | NA | NA | **-0.320** | **0.101** | **-0.516, -0.132** |

**Table S3:** Effects of the three different life-history traits on EPP, and the effects of the life-history traits and EPP on male provisioning rates, based upon 106 species. Phylogenetic path coefficients (Estimate), their standard error (SE) and 95% confidence intervals (CI) are shown. Significant results are marked in bold.

|  | EPP |  |  | Provisioning rate | | |
| --- | --- | --- | --- | --- | --- | --- |
|  | Estimate | SE | CI | Estimate | SE | CI |
| Maximum lifespan | -0.234 | 0.121 | -0.486, 0.006 | 0.131 | 0.124 | -0.101, 0.367 |
| Clutch size | -0.153 | 0.112 | -0.377, 0.064 | 0.063 | 0.113 | -0.172, 0.263 |
| Body mass | -0.005 | 0.113 | -0.220, 0.196 | -0.185 | 0.113 | -0.419, 0.006 |
| EPP | NA | NA | NA | **-0.321** | **0.099** | **-0.525, -0.135** |

**Table S4:** Phylogenetic signals (Pagel’s λ values estimated using maximum likelihood within the boundaries 0 and 1 with 95% CI) in EPP rates, life-history traits and paternal care traits. The sample size (n) showing the number of species included in the phylogenetic analysis for each trait is also given.

| Trait | Pagel’s λ | 95% CI | *n* |
| --- | --- | --- | --- |
| EPP | 0.56 | 0.36, 0.73 | 271 |
| ***Life history traits*** |  |  |  |
| Body mass | 0.91 | 0.86, 0.95 | 266 |
| Maximum lifespan | 0.78 | 0.65, 0.87 | 259 |
| Clutch size | 1.00 | 0.90, 1.00 | 268 |
| ***Paternal care traits*** |  |  |  |
| Nestbuilding (binomial) | 1.00 | 0.92, 1.00 | 191 |
| Incubation (binomial) | 0.89 | 0.79, 0.95 | 259 |
| Nestbuilding | 1.00 | 0.99, 1.00 | 116 |
| Nestbuilding^*^ | 1.00 | 0.99, 1.00 | 115 |
| Incubation | 1.00 | 0.99, 1.00 | 196 |
| Incubation* | 1.00 | 0.99, 1.00 | 187 |
| Provisioning rate | 0.68 | 0.34, 0.89 | 109 |
| Provisioning rate* | 0.68 | 0.35, 0.90 | 104 |
| Biomass delivery rate | 1.00 | 0.75, 1.00 | 29 |
| Biomass delivery rate* | 1.00 | 0.77, 1.00 | 28 |

* Parental care trait where only studies reporting values are included.

**Table S5**: Pearson’s phylogenetic correlation coefficients for EPP, body mass, lifespan and clutch size for 259 species (excluding species with any missing values).

|  | EPP | Bodymass | Lifespan | Clutch size |
| --- | --- | --- | --- | --- |
| EPP |  | -0.163 (P=0.008) | -0.303 (P<0.001) | 0.053 (P=0.392) |
| Bodymass |  |  | 0.574 (P<0.001) | -0.349 (P<0.001) |
| Lifespan |  |  |  | -0.410 (P<0.001) |

**Table S6:** Effects of the three different life-history traits on EPP, and the effects of the life-history traits and EPP on occurrence of male nestbuilding, based upon 185 species. Phylogenetic path coefficients (Estimate), their standard error (SE) and 95% confidence intervals (CI) are shown. Significant results are marked in bold.

|  | EPP |  | |  | | Nestbuilding | | | |  |
| --- | --- | --- | --- | --- | --- | --- | --- | --- | --- | --- |
|  | Estimate | | SE | | CI | | Estimate | SE | CI | |
| Maximum lifespan | **-0.214** | | **0.097** | | **-0.391, -0.024** | | 0.051 | 0.259 | -0.323, 0.632 | |
| Clutch size | -0.074 | | 0.084 | | -0.226, 0.074 | | **-0.329** | **0.186** | **-0.697, -0.014** | |
| Body mass | -0.021 | | 0.091 | | -0.226, 0.158 | | 0.934 | 0.782 | -0.152, 1.207 | |
| EPP | NA | | NA | | NA | | **-0.357** | **0.165** | **-0.665, -0.069** | |

**Table S7:** Effects of the three different life-history traits on EPP, and the effects of the life-history traits and EPP on proportion of male nestbuilding, based upon 111 species. Phylogenetic path coefficients (Estimate), their standard error (SE) and 95% confidence intervals (CI) are shown. Significant results are marked in bold.

|  | EPP |  | |  | | Nestbuilding | | | |
| --- | --- | --- | --- | --- | --- | --- | --- | --- | --- |
|  | Estimate | | SE | | CI | | Estimate | SE | CI |
| Maximum lifespan | **-0.244** | | **0.099** | | **-0.421, -0.045** | | -0.007 | 0.065 | -0.132, 0.122 |
| Clutch size | -0.207 | | 0.113 | | -0.399, 0.012 | | -0.066 | 0.086 | -0.212, 0.094 |
| Body mass | 0.002 | | 0.102 | | -0.214, 0.196 | | -0.073 | 0.076 | -0.215, 0.082 |
| EPP | NA | | NA | | NA | | -0.028 | 0.058 | -0.130, 0.080 |

**Table S8:** Effects of the three different life-history traits on EPP, and the effects of the life-history traits and EPP on occurrence of male nestbuilding, based upon 141 Passerine species. Phylogenetic path coefficients (Estimate), their standard error (SE) and 95% confidence intervals (CI) are shown. Significant results are marked in bold.

|  | EPP |  | |  | | Nestbuilding | | | |  |
| --- | --- | --- | --- | --- | --- | --- | --- | --- | --- | --- |
|  | Estimate | | SE | | CI | | Estimate | SE | CI | |
| Maximum lifespan | **-0.188** | | **0.081** | | **-0.358, -0.036** | | -0.068 | 0.145 | -0.398, 0.205 | |
| Clutch size | -0.096 | | 0.105 | | -0.307, 0.121 | | -0.104 | 0.185 | -0.518, 0.270 | |
| Body mass | -0.028 | | 0.093 | | -0.221, 0.159 | | 0.137 | 0.231 | -0.147, 0.910 | |
| EPP | NA | | NA | | NA | | -0.293 | 0.158 | -0.663, 0.021 | |

**Table S9:** Effects of the three different life-history traits on EPP, and the effects of the life-history traits and EPP on proportion of male nestbuilding, based upon 99 Passerine species. Phylogenetic path coefficients (Estimate), their standard error (SE) and 95% confidence intervals (CI) are shown. Significant results are marked in bold.

|  | EPP |  | |  | | Nestbuilding | | | |
| --- | --- | --- | --- | --- | --- | --- | --- | --- | --- |
|  | Estimate | | SE | | CI | | Estimate | SE | CI |
| Maximum lifespan | **-0.229** | | **0.090** | | **-0.394, -0.058** | | -0.026 | 0.057 | -0.124, 0.084 |
| Clutch size | **-0.253** | | **0.122** | | **-0.507, -0.005** | | -0.057 | 0.088 | -0.255, 0.114 |
| Body mass | 0.051 | | 0.105 | | -0.134, 0.238 | | -0.084 | 0.086 | -0.247, 0.085 |
| EPP | NA | | NA | | NA | | -0.045 | 0.061 | -0.156, 0.075 |

**Table S10:** Effects of the three different life-history traits on EPP, and the effects of the life-history traits and EPP on occurrence of male incubation, based upon 251 socially monogamous species. Phylogenetic path coefficients (Estimate), their standard error (SE) and 95% confidence intervals (CI) are shown. Significant results are marked in bold.

|  | EPP |  |  | Incubation | | |
| --- | --- | --- | --- | --- | --- | --- |
|  | Estimate | SE | CI | Estimate | SE | CI |
| Maximum lifespan | -0.156 | 0.079 | -0.316, 0.007 | -0.139 | 0.177 | -0.470, 0.155 |
| Clutch size | -0.016 | 0.076 | -0.176, 0.130 | **-0.468** | **0.198** | **-0.980, -0.092** |
| Body mass | 0.102 | 0.083 | -0.072, 0.256 | **0.823** | **0.284** | **0.384, 1.446** |
| EPP | NA | NA | NA | **-0.419** | **0.181** | **-0.967, -0.040** |

**Table S11:** Effects of the three different life-history traits on EPP, and the effects of the life-history traits and EPP on proportion of male incubation, based upon 190 species. Phylogenetic path coefficients (Estimate), their standard error (SE) and 95% confidence intervals (CI) are shown. Significant results are marked in bold.

|  | EPP |  | |  | | Incubation | | | |
| --- | --- | --- | --- | --- | --- | --- | --- | --- | --- |
|  | Estimate | | SE | | CI | | Estimate | SE | CI |
| Maximum lifespan | **-0.194** | | **0.081** | | **-0.346, -0.040** | | -0.071 | 0.048 | -0.155, 0.026 |
| Clutch size | -0.015 | | 0.084 | | -0.182, 0.148 | | -0.060 | 0.055 | -0.157, 0.044 |
| Body mass | **0.168** | | **0.083** | | **0.009, 0.322** | | **0.267** | **0.061** | **0.144, 0.393** |
| EPP | NA | | NA | | NA | | -0.061 | 0.043 | -0.146, 0.013 |

**Table S12:** Effect of the three life-history traits on EPP, and the effects of the life-history traits and EPP on proportion male incubation, based upon 182 species. Included are only studies that provided proportion incubation values for males and females. Phylogenetic path coefficients (Estimate), their standard error (SE) and 95% confidence intervals (CI) are shown. Significant results are marked in bold.

|  | EPP |  | |  | | Incubation | | | |
| --- | --- | --- | --- | --- | --- | --- | --- | --- | --- |
|  | Estimate | | SE | | CI | | Estimate | SE | CI |
| Maximum lifespan | **-0.219** | | **0.089** | | **-0.405, -0.044** | | -0.051 | 0.060 | -0.165, 0.063 |
| Clutch size | -0.029 | | 0.085 | | -0.208, 0.127 | | -0.057 | 0.058 | -0.171, 0.051 |
| Body mass | **0.172** | | **0.085** | | **0.019, 0.323** | | **0.277** | **0.066** | **0.145, 0.403** |
| EPP | NA | | NA | | NA | | **-0.058** | **0.046** | **-0.146, 0.032** |

**Table S13:** Effects of the three different life-history traits on EPP, and the effects of the life-history traits and EPP on occurrence of male incubation, based upon 142 Passerine monogamous species. Phylogenetic path coefficients (Estimate), their standard error (SE) and 95% confidence intervals (CI) are shown. Significant results are marked in bold.

|  | EPP |  |  | Incubation | | |
| --- | --- | --- | --- | --- | --- | --- |
|  | Estimate | SE | CI | Estimate | SE | CI |
| Maximum lifespan | **-0.172** | **0.081** | **-0.315, -0.005** | -0.034 | 0.231 | -0.526, 0.294 |
| Clutch size | -0.080 | 0.104 | -0.299, 0.153 | -0.414 | 0.356 | -0.940, 0.107 |
| Body mass | -0.026 | 0.092 | -0.204, 0.140 | -0.149 | 0.373 | -0.831, 0.234 |
| EPP | NA | NA | NA | -0.599 | 0.331 | -1.204, 0.000 |

**Table S14:** Effect of the three life-history traits on EPP, and the effects of the life-history traits and EPP on proportion of male incubation, based upon 128 Passerine species. Phylogenetic path coefficients (Estimate), their standard error (SE) and 95% confidence intervals (CI) are shown. Significant results are marked in bold.

|  | EPP |  | |  | | Incubation | | | |
| --- | --- | --- | --- | --- | --- | --- | --- | --- | --- |
|  | Estimate | | SE | | CI | | Estimate | SE | CI |
| Maximum lifespan | **-0.193** | | **0.084** | | **-0.364, -0.021** | | -0.027 | 0.070 | -0.151, 0.105 |
| Clutch size | -0.070 | | 0.111 | | -0.267, 0.109 | | -0.155 | 0.094 | -0.355, 0.008 |
| Body mass | -0.041 | | 0.097 | | -0.221, 0.171 | | -0.114 | 0.090 | -0.268, 0.061 |
| EPP | NA | | NA | | NA | | -0.127 | 0.071 | -0.262, 0.006 |

**Table S15:** Effect of life history traits on EPP and effects of life history traits and EPP on proportion of male provisioning based on 72 Passerine species. Phylogenetic path coefficients (estimate), their standard error (SE) and 95% confidence intervals (CI) are shown. Significant results are marked in bold.

|  | EPP |  |  | Provisioning rate | | |
| --- | --- | --- | --- | --- | --- | --- |
|  | Estimate | SE | CI | Estimate | SE | CI |
| Maximum lifespan | -0.138 | 0.110 | -0.345, 0.073 | 0.167 | 0.108 | -0.043, 0.381 |
| Clutch size | -0.127 | 0.132 | -0.389, 0.132 | **0.209** | **0.108** | **0.002, 0.390** |
| Body mass | 0.113 | 0.127 | -0.131, 0.356 | -0.029 | 0.110 | -0.247, 0.161 |
| EPP | NA | NA | NA | **-0.408** | **0.107** | **-0.602, -0.190** |

**Table S16:** Effects of the three different life-history traits on EPP, and the effects of the life-history traits and EPP on male biomass delivery rate, based upon 29 species. Phylogenetic path coefficients (Estimate), their standard error (SE) and 95% confidence intervals (CI) are shown. Significant results are marked in bold.

|  | EPP |  | |  | | Biomass delivery rate | | | |
| --- | --- | --- | --- | --- | --- | --- | --- | --- | --- |
|  | Estimate | | SE | | CI | | Estimate | SE | CI |
| Maximum lifespan | **-0.605** | | **0.294** | | **-1.130, -0.015** | | -0.253 | 0.304 | -0.775, 0.319 |
| Clutch size | -0.124 | | 0.197 | | -0.464, 0.199 | | 0.037 | 0.210 | -0.343, 0.422 |
| Body mass | 0.451 | | 0.282 | | -0.064, 1.012 | | 0.364 | 0.282 | -0.135, 0.878 |
| EPP | NA | | NA | | NA | | **-0.412** | **0.164** | **-0.691, -0.069** |

**Table S17:** Effect of the three life-history traits on EPP, and the effects of the life-history traits and EPP on male biomass delivery rate, based upon 28 species. Included are only studies that provided biomass delivery values for males and females. Phylogenetic path coefficients (Estimate), their standard error (SE) and 95% confidence intervals (CI) are shown. Significant results are marked in bold.

|  | EPP |  | |  | | Biomass delivery rate | | | |
| --- | --- | --- | --- | --- | --- | --- | --- | --- | --- |
|  | Estimate | | SE | | CI | | Estimate | SE | CI |
| Maximum lifespan | -0.597 | | 0.306 | | -1.163, 0.010 | | -0.243 | 0.312 | -0.942, 0.277 |
| Clutch size | -0.122 | | 0.199 | | -0.524, 0.253 | | 0.033 | 0.206 | -0.357, 0.410 |
| Body mass | 0.450 | | 0.301 | | -0.149, 1.024 | | 0.359 | 0.292 | -0.163, 0.982 |
| EPP | NA | | NA | | NA | | **-0.409** | **0.166** | **-0.708, -0.108** |

**Table S18:** Effect of the three life-history traits on EPP, and the effects of the life-history traits and EPP on male biomass delivery rate, based upon 15 Passerine species. Phylogenetic path coefficients (Estimate), their standard error (SE) and 95% confidence intervals (CI) are shown. Significant results are marked in bold.

|  | EPP |  | |  | | Biomass delivery rate | | | |
| --- | --- | --- | --- | --- | --- | --- | --- | --- | --- |
|  | Estimate | | SE | | CI | | Estimate | SE | CI |
| Maximum lifespan | -0.339 | | 0.313 | | -0.863, 0.194 | | -0.024 | 0.193 | -0.405, 0.352 |
| Clutch size | -0.241 | | 0.307 | | -0.734, 0.337 | | 0.067 | 0.329 | -0.488, 0.601 |
| Body mass | 0.020 | | 0.264 | | -0.408, 0.476 | | -0.071 | 0.250 | -0.479, 0.392 |
| EPP | NA | | NA | | NA | | -0.395 | 0.279 | -0.806, 0.063 |
