# Supplementary material for: The evolution of extra-pair paternity and paternal care in birds
Source: Behav Ecol. 2023 Jun 23;34(5):780–9. doi: 10.1093/beheco/arad053 (PMC10516673; doi:10.1093/beheco/arad053)
Supplement: arad053_suppl_Supplementary_Table_S1 [file arad053_suppl_supplementary_table_s1.pdf]

Supplementary materials

Table S1: Data and references for EPP-rates (measured as percentage of offspring sired by an extra-pair male), with male participation in nestbuilding and incubation (0 = male does not participate, 1 = male does participate), proportion of provisioning events (Pr (%)) and proportion of biomass (Biomass (%)) delivered by male. Rates noted with * indicate where at least one reference only gave interpreted result (e.g., "equal") proportion of provisioning events.

Latin	English name	EPP (%)	Ref. EPP	NB	Ref. NB	Inc	Ref. Inc	Pr (%)	Ref. Pr	Biomass (%)	Ref. biomass
Acanthiza pusilla	Brown thornbill	6.2	[1]	0	[2]	0	[2]	0.48	[3]		
Accipiter cooperii	Cooper's hawk	19.3	[1]	1	[2]	1	[2]				
Acrocephalus arundinaceus	Great reed warbler	4.8	[1]	0	[4]	0	[4]	0.4175	[5,6]		
Acrocephalus bistrigiceps	Black-browed reed-warbler	6.4	[1]	0	[7]	0	[7]				
Acrocephalus palustris	Marsh warbler	3.1	[1]	0	[4]	1	[4]	0.392	[5]		
Acrocephalus schoenobaenus	Sedge warbler	8.1	[1]	0	[4]	0	[4]	0.4715	[5,8]		
Acrocephalus scirpaceus	Eurasian reed-warbler	6.5	[1]	0	[4]	1	[4]	0.543	[9]		
Actitis hypoleucos	Common sandpiper	12.5	[1]	1	[4]	1	[4]				
Aegolius funereus	Boreal owl	0	[1]			0	[4]				
Agelaius phoeniceus	Red-winged blackbird	27.8	[1]	0	[2]	0	[2]				
Agelaius xanthomus	Yellow-shouldered blackbird	23	[1]	0	[2]	0	[2]				
Alauda arvensis	Skylark	20.5	[1]	0	[4]	0	[4]				
Alle alle	Little auk	2.3	[1]			1	[4]				
Ammodramus maritimus	Seaside sparrow	10.6	[1]	0	[2]	0	[2]				
Ammodramus savannarum	Grasshopper Sparrow	36.8	[10]	0	[11]	0	[2]	0.4626	[12]		
Anas platyrhynchos	Mallard	9.3	[1]	0	[4]	0	[4]				
Anas strepera	Gadwalls	4.2	[1]	0	[4]	0	[4]				

Anthus spinoletta	Water pipit	5.2	[1]	0	[4]	0	[4]	0.4495	[13]		
Aphrastura spinicauda	Thorn-tailed Rayadito	8.3	[14]	1	[15]	1	[15]	0.4930	[15]		
Apus apus	Common swift	4.5	[1]	1	[4]	1	[4]	0.4533	[16]		
Aquila heliaca	Eastern imperial eagle	0	[1]	1	[4]	1	[4]	0.3480	[17]		
Ardea alba	Great egret	0	[1]	1	[4]	1	[4]				
Asio otus	Long-eared owl	0	[1]			0	[4]				
Athene cunicularia	Burrowing owl	1.7	[1]			0	[2]				
Athene noctua	Little owl	0	[1]			0	[4]				
Baeolophus bicolor	Tufted titmouse	8.8	[1]	0	[2]	0	[2]				
Bartramia longicauda	Upland sandpiper	18.1	[1]			1	[2]				
Branta bernicla	Black brant	5.6	[1]	1	[4]	0	[4]				
Branta leucopsis	Barnacle goose	0	[1]	1	[4]	0	[4]				
Buteo ridgwayi	Ridgway's hawk	0	[1]	1	[18]	1	[18]	0.9	[18]		
Buteo swainsoni	Swainson’s hawk	5.4	[1]	1	[19]	0	[19]				
Calamospiza melanocorys	Lark bunting	25.1	[1]	0	[2]	0	[2]				
Calidris alba	Sanderling	2	[1]	0	[4]	1	[4]				
Calidris maritima	Purple sandpiper	1.2	[1]	1	[4]	1	[4]				
Calidris mauri	Western sandpiper	6.6	[1]			1	[4]				
Calonectris diomedea	Cory's shearwater	5.4	[1]			1	[20]	0.5*	[21]	0.5*	[21]
Cardinalis cardinalis	Northern cardinal	13.5	[1]	0	[2]	0	[2]	0.5469	[22,23]		
Carduelis cannabina	Eurasian linnet	3.8	[1]	0	[4]	0	[4]				
Carduelis tristis	American goldfinch	14.3	[1]	0	[2]	0	[2]				
Carpodacus erythrinus	Scarlet rosefinch	16.1	[1]	0	[2]	0	[2]	0.4837	[24,25]		
Carpodacus mexicanus	House finch	8.8	[1]	0	[2]	0	[2]	0.595	[26,27]		

Catharacta lonnbergi	Brown skua	0	[1]			1	[28]		
Catharacta maccormicki	South polar skua	7.1	[1]			0	[29]	0.5757	[29]
Centropus phasianinus	Pheasant coucal	18.6	[1]	1	[30]	1	[2]	0.8143	[30,31]
Cepphus grylle	Black guillemot	0	[1]			1	[4]	0.4862	[32]
Cercomacra tyrannina	Dusky antbird	0	[1]			1	[2]		
Charadrius alexandrinus	Kentish plover	0.9	[1]	1	[4]	1	[4]		
Charadrius falklandicus	Two-banded plover	0	[1]			1	[33]		
Charadrius hiaticula	Ringed plover	0	[1]	1	[4]	1	[4]		
Charadrius marginatus	White-fronted plover	0	[1]			1	[2]		
Charadrius modestus	Rufous-chested dotterel	0	[1]			1	[2]		
Charadrius nivosus	Snowy plover	0	[1]			1	[34]		
Charadrius pecuarius	Kittlitz's plover	0	[1]			1	[4]		
Charadrius ruficapillus	Red-capped plover	0	[1]			0	[2]		
Charadrius semipalmatus	Semipalmated plover	4.7	[1]			1	[4]		
Charadrius thoracicus	Madagascar plover	0	[1]			1	[35]		
Chen caerulescens	Lesser snow goose	5	[1]						
Chen rossii	Ross's goose	2.4	[1]			0	[36]		
Chlidonias hybrida	Whiskered tern	8.1	[1]	1	[4]	1	[4]		
Chlidonias niger	North American black tern	0	[1]	1	[37]	1	[37]		
Cinclus cinclus	White-throated dipper	1.6	[1]	1	[4]	0	[4]		
Circus pygargus	Montagu's harrier	3.1	[1]	0	[4]	0	[4]		
Cistothorus platensis	Grass wrens	19.43	[38]	1	[39]	0	[40]		
Coracias garrulus	European roller	5.3	[1]			1	[4]	0.5071	[41,42]
Coragyps atratus	Black vulture	0	[1]			1	[2]		

Corvus corone	Carrion crow	2.9	[43]	1	[2]	0	[2]				
Corvus monedula	Eurasian jackdaw	0.9	[1]	1	[4]	0	[4]				
Cyanocitta stelleri	Steller's jay	15-2	[1]	1	[2]	0	[2]				
Cyanoliseus patagonus	Burrowing parakeet	0	[1]			0	[44]				
Cygnus atratus	Black swan	15.1	[1]			1	[45]				
Delichon urbicum	House martin	18.8	[1]	1	[4]	1	[4]	0.4902	[46,47]		
Dendrocopos major	Great spotted woodpecker	0	[1]	1	[4]	1	[4]	0,5333	[48]		
Dendrocopos medius	Middle spotted woodpecker	0	[1]	1	[4]	1	[4]	0.4931	[48]		
Dendroica caerulescens	Black-throated blue warbler	22.7	[1]	0	[49]	0	[49]	0.4196	[50]	0.5479	[50,51]
Dendroica pensylvanica	Chestnut-sided warbler	47.4	[1]	0	[52]	0	[52]				
Dendroica petechia	Yellow warbler	31	[1]	0	[53]	0	[53]	0.5538	[54]		
Diomedea exulans	Wandering albatross	15.8	[1]			1	[55]			0.54	[56]
Dolichonyx oryzivorus	Bobolinks	42	[57]	0	[2]	0	[2]				
Dumetella carolinensis	Grey catbird	13	[1]	0	[2]	0	[2]	0.49	[58]		
Elaenia chiriquensis	Lesser elaenia	36.8	[1]	0	[59]	0	[59]				
Elaenia flavogaster	Yellow-bellied elaenia	4.2	[1]	1	[59].	0	[59]				
Emberiza citrinella	Yellowhammer	37.4	[1]	0	[4]	0	[4]	0.4145	[60]		
Emberiza schoeniclus	Reed bunting	40.4	[1]	0	[4]	0	[4]	0.39	[61,62]		
Empidonax minimus	Least flycatcher	34.9	[1]	0	[63]	0	[63]				
Empidonax traillii	Willow flycatcher	14.3	[1]	0	[64]	0	[64].				
Empidonax virescens	Acadian flycatcher	38.9	[1]	0	[65]	0	[65]				
Erithacus rubecula	European Robin	4	[66]	0	[4]	0	[4]				
Erythrura gouldiae	Gouldian finch	8.6	[1]			1	[2]	0.5	[67]		
Eudromias morinellus	Eurasian dotterel	4.5	[1]	1	[4]	1	[4]	1	[4]	1	[4]

Eudyptes pachyrhynchus	Fiordland penguin	0	[1]			1	[2]				
Eudyptes schlegeli	Royal penguin	3.8	[1]			1	[2]				
Euplectes orix	Red bishop	22.1	[1]	1	[2]	0	[2]	0	[2]	0	[2]
Falco columbarius	Merlin	0	[1]			1	[4]				
Falco eleonorae	Eleonoras falcon	0	[1]			0	[4]				
Falco naumanni	Lesser kestrel	7.3	[1]			0	[4]	0.615	[68]		
Falco peregrinus	Peregrine falcon	0	[1]			1	[4]	0.85	[69]	0.71	[69]
Falco sparverius	American kestrel	11.2	[1]			1	[70]	0.37	[71,72]		
Falco tinnunculus	Eurasian kestrel	1.9	[1]			0	[4]	0.8539	[73]		
Ficedula albicollis	Collared flycatcher	21.0	[1]	0	[4]	0	[4]	0.4761	[74,75]	0.4966	[76]
Ficedula hypoleuca	Pied flycatcher	9.4	[1]	0	[4]	0	[4]	0.4384	[77,78]	0.5396	[78]
Ficedula parva	Red-breasted flycatcher	7.5	[1]	0	[4]	0	[4]	0.59	[79]		
Ficedula zanthopygia	Yellow rumped flycatcher	22.2	[1]	0	[80]	0	[81]				
Fratercula arctica	Atlantic puffin	0	[1]	1	[4]	1	[4]				
Fregata minor	Great frigatebird	3.6	[1]	1	[82]	1	[82]				
Fringilla coelebs	Chaffinch	17	[1]	0	[4]	0	[4]				
Fulmarus glacialis	Northern fulmar	0	[1]			1	[4]				
Furnarius rufus	Rufous Hornero	3.33	[83]	1	[84]	1	[84]				
Gallinula chloropus	Moorhen	0	[1]	1	[4]	1	[4]	0.4268	[85]		
Gavia immer	Common loon	0	[1]	1	[4]	1	[4]				
Geospiza fortis	Medium ground-finch	17.1	[1]	1	[2]						
Geospiza scandens	Cactus finch	10.3	[1]	1	[2]	0	[2]				
Geothlypis trichas	Yellowthroat	19.1	[1]	0	[86]	0	[86]	0.5382	[87]		
Gerygone flavolateralis	Fan-tailed Gerygone	8.7	[88]								

Grallina cyanoleuca	Australian magpie-lark	2.9	[1]	1	[2]	1	[2]	0.5647	[89]		
Grus canadensis	Sandhill crane	11.1	[1]	1	[90]	1	[90]				
Gyps fulvus	Griffon vulture	0	[1]	1	[4]	1	[4]	0.5307	[91]		
Habia fuscicauda	Red-throated ant-tanager	41.5	[1]	0	[2]	0	[2]				
Haematopus ostralegus	Eurasian oystercatcher	1.5	[1]	1	[4]	1	[4]				
Haliaeetus albicilla	White-tailed eagle	0	[92]			1	[4]				
Hirundo ariel	Fairy martin	13.8	[1]	1	[2]	1	[2]				
Hirundo rustica	Barn swallow	24.3	[1]	1	[4]	0	[4]	0.5033	[47,93]		
Hylocichla mustelina	Wood thrush	20.5	[1]	0	[94]	0	[94]				
Hymenolaimus malacorhynchos	Blue duck	0	[1]			0	[95]				
Icteria virens	Yellow-breasted chat	30.8	[1]	0	[96]	0	[96]	0.5253	[97]	0.5155	[97]
Icterus galbula	Bullock´s oriole	32.2	[1]	0	[98]	0	[98]				
Jabiru mycteria	Jabiru	2.9	[1]	1	[99]	1	[99]				
Junco hyemalis	Dark-eyed junco	27.6	[1]	0	[2]	0	[2]				
Jynx torquilla	Eurasian wryneck	0.7	[1]			1	[4]				
Lagopus lagopus	Willow ptarmigan	9.4	[1]			0	[2]				
Lagopus leucura	White-tailed ptarmigan	5.2	[1]			0	[100]				
Laniarius atrococcineus	Crimson-breasted shrike	18.9	[1]	1	[2]						
Lanius bucephalus	Bull-headed shrike	10.1	[1]	1	[101]	0	[102]	0.629	[103]		
Lanius ludovicianus	Loggerhead shrike	4.5	[1]	0	[2]	0	[2]				
Lanius minor	Lesser gray shrike	0	[1]	1	[4]	0	[4]				
Larus canus	Common gull	3.6	[1]	1	[4]	1	[4]				
Larus occidentalis	Western gull	0	[1]	1	[104]	1	[104]	0.556	[105]	0.6266	[105]
Larus ridibundus	Black-headed gull	20.3	[1]	1	[4]	1	[4]				

Lichenostomus chrysops	Yellow-faced honeyeater	44.4	[1]	0	[2]	0	[2]				
Locustella luscinioides	Savi's warbler	4.1	[1]	1	[4]	1	[4]				
Loxia curvirostra	Red crossbill	0	[1]	0	[4]	0	[4]				
Loxioides bailleui	Palila	0	[1]	1	[2]	0	[2]	0.37	[106]		
Luscinia megarhynchos	Common nightingale	21.5	[1]					0.5063	[91]		
Luscinia svecica	Bluethroat	27.5	[1]	0	[107]	0	[107]	0.473	[108]		
Megascops asio	Eastern screech-owl	0	[1]			0	[2]	0.4095	[109]	0.4095	[109]
Melospiza georgiana	Swamp sparrow	20.9	[1]	0	[2]	0	[2]				
Melospiza melodia	Song sparrow	27.9	[1]	0	[2]	0	[2]	0.4722	[110–112]		
Miliaria calandra	Corn bunting	4.5	[1]	0	[4]	0	[4]	0.142	[113]		
Mycteria americana	Wood stork	0	[1]	1	[114]	1	[114]				
Myiopsitta monachus	Monk parakeet	0	[1]	1	[115]	0	[115]				
Nectarinia osea	Orange-tufted sunbird	22.7	[1]	0	[2]	0	[2]	0.25	[116]		
Notiomystis cincta	New Zealand hihi	65.2	[1]	0	[2]	0	[2]				
Oceanites oceanicus	Wilson's storm-petrel	0	[1]			1	[117]	0.7514	[117]	0.5084	[117]
Oceanodroma leucorhoa	Leach's storm-petrel	0	[1]			1	[118]	0.623	[118]		
Oenanthe oenanthe	Northern wheatear	16.5	[1]	0	[4]	0	[4]	0.5276	[119]	0.5586	[120]
Otus elegans	Elegant scops-owl	0.5	[1]			0	[121]				
Otus flammeolus	Flammulated owl	0	[1]			0	[2]				
Pachycephala pectoralis	Golden whistlers	19.2	[1]	0	[2]	1	[2]				
Pachyptila belcheri	Thin-billed prion	20.6	[1]					0.5*	[122,123]	0.5086*	[122,123]
Panurus biarmicus	Bearded tit	14.4	[1]	1	[4]	1	[4]	0.543	[124]		
Paradoxornis webbianus	Vinous-throated parrotbills	7.7	[1]	1	[2]	1	[2]	0.54	[125]		
Parus ater	Coal tit	31.2	[1]	0	[4]	0	[4]				

Parus atricapillus	Black-capped chickadee	11.8	[1]	0	[2]	0	[2]	0.63	[126]		
Parus caeruleus	Blue tit	13.05 0	[1,127]	0	[4]	0	[4]	0.4891	[128–131]		
Parus cristatus	Crested tit	11	[1]	0	[4]	0	[4]				
Parus major	Great tit	9.3	[1]	0	[4]	0	[4]	0.5721	[132–135]	0.5555	[132]
Parus montanus	Willow tit	6.0	[1]	0	[4]	0	[4]				
Parus teneriffae	African blue tit	15.3	[1]								
Parus varius	Varied tit	14.7	[1]	1	[2]	0	[2]				
Passer domesticus	House sparrow	15.8	[1]	1	[4]	1	[4]	0.4629	[136–139]		
Passer montanus	Tree sparrow	9.1	[1]	1	[4]	1	[4]				
Passerculus sandwichensis	Savannah sparrow	44.4	[1]	0	[2]	0	[2]	0.3454	[140–142]	0.2825	[140]
Passerina caerulea	Blue grosbeak	52.7	[1]	0	[2]	0	[2]	0.1361	[143]		
Passerina cyanea	Indigo bunting	34.9	[1]	0	[2]	0	[2]	0	[2]	0	[2]
Perisoreus canadensis	Canada jay	0	[144]	1	[145]	0	[145]				
Petroica australis	New Zealand robin	0.4	[1]	0	[2]	0	[2]	0.4249	[146]		
Petroica goodenovii	Red-capped robin	22.1	[1]	0	[2]	0	[2]				
Petronia petronia	Rock sparrow	27.5	[1]	0	[4]	0	[4]	0.3818	[147,148]		
Phainopepla nitens	Phainopepla	0	[1]	1	[149]	1	[149]				
Phalacrocorax aristotelis	Shag	9.3	[1]	1	[4]	1	[4]				
Phalacrocorax atriceps	Imperial shag	0	[1]								
Phalacrocorax carbo	Great cormorant	10.5	[1]	1	[4]	1	[4]				
Phalaropus lobatus	Red-necked phalarope	1.8	[1]	1	[4]	1	[4]	1	[4]	1	[4]
Philesturnus carunculatus	Saddleback	0	[1]	0	[2]	0	[2]				
Phoebastria irrorata	Waved albatross	17.6	[1]			1	[150]				

Phoenicurus ochruros	Black redstart	28.8	[1]	0	[4]	0	[4]	0.4385	[151]		
Phoenicurus phoenicurus	Common redstart	2	[1]	0	[4]	0	[4]	0.5403	[152]		
Phylidonyris pyrrhopterus	Crescent honeyeater	57.9	[1]	0	[2]	0	[2]	0.309	[153]		
Phylloscopus fuscatus	Dusky warbler	45.1	[1]	0	[154]	0	[154]				
Phylloscopus sibilatrix	Wood warbler	1.6	[1]	0	[4]	0	[4]	0.5657	[155]	0.5	[155]
Phylloscopus trochilus	Willow warbler	20.5	[1]	0	[4]	0	[4]	0.3642	[156]		
Picoides tridactylus	Eurasian three-toed woodpecker	4.4	[1]	1	[4]	1	[4]	0.7251	[157]		
Pipilo maculatus	Spotted towhee	26.3	[1]	0	[2]	0	[2]	0.4306	[158]		
Piranga olivacea	Scarlet tanager	16.7	[1]	0	[2]	0	[2]				
Platalea ajaja	Roseate spoonbill	2.7	[1]	1	[159]	1	[159]				
Platycercus elegans	Crimson Rosella	0	[160]			0	[161]				
Plectrophenax nivalis	Snow bunting	10.8	[1]	0	[4]	0	[4]	0.4597	[162–164]	0.5341	[162]
Pluvialis dominica	American golden plover	7.6	[1]			1	[2]				
Poecile gambeli	Mountain chickadees	17.9	[165]			0	[2]				
Poecile palustris	Marsh Tits	15.8	[166]	0	[2]	0	[2]				
Poephila acuticauda	Long-tailed finch	12.8	[1]			1	[167]				
Porphyrio hochstetteri	Takahe	0	[1]	1	[168]	1	[168]				
Prinia flaviventris	Yellow-bellied prinia	14.46	[169]	1	[2]	1	[2]				
Progne subis	Purple martin	21.8	[1]	1	[2]	0	[2]	0.4828	[170]		
Promerops cafer	Cape sugarbird	64.9	[1]	0	[2]	0	[2]				
Prosthemadera novaeseelandiae	Tui	55.2	[1]	1	[2]	0	[2]				
Puffinus tenuirostris	Short-tailed shearwater	10.8	[1]			1	[171]				
Pygoscelis adeliae	Adélie penguin	9.1	[1]			1	[2]				
Pygoscelis antarcticus	Chinstrap penguin	0	[1]			1	[2]				

Pygoscelis papua	Gentoo penguin	48.75	[172]			1	[2]				
Quelea quelea	Red-billed quelea	21.4	[1]	1	[2]						
Ramphocelus costaricensis	Cherrie's tanager	49.1	[1]	0	[2]	0	[2]				
Remiz coronatus	White-crowned penduline tit	0	[1]	1	[2]	0	[2]				
Rhipidura fuliginosa	Grey fantail	55.1	[1]	1	[2]	1	[2]				
Riparia riparia	Sand martin	17	[1]	1	[4]	1	[4]	0.5839	[173]		
Rissa tridactyla	Black-legged kittiwake	0	[1]	1	[4]	1	[4]	0.5*	[174]		
Sayornis phoebe	Eastern phoebe	5.7	[1]	0	[2]			0.4626*	[175,176]		
Serinus canaria	Canary	0	[1]	0	[4]	0	[4]				
Serinus serinus	Serin	6.5	[1]	0	[4]	0	[4]				
Setophaga ruticilla	American redstart	31.5	[1]	0	[177]	0	[177]	0.53	[178]	0.61	[178]
Sialia currucoides	Mountain bluebird	33.5	[1]	0	[179]	0	[179]	0.4529	[180,181]		
Sialia sialis	Eastern bluebird	8.4	[1]	0	[182]	0	[182]	0.5049	[183]		
Sicalis flaveola	Saffron finch	31.8	[184]	1	[185]	0	[185]	0.4518	[186]		
Sitta europaea	European nuthatch	9.6	[1]	0	[4]	0	[4]	0.71	[187]		
Spheniscus humboldti	Humboldt penguin	0	[1]			1	[188]				
Spheniscus magellanicus	Magellanic Penguin	31	[189]			1	[2]				
Spiza americana	Dickcissel	38.5	[1]	0	[2]	0	[2]	0	[2]	0	[2]
Spizella pusilla	Field sparrow	9.7	[1]	0	[2]	0	[2]	0.4875	[190]	0.5456	[190]
Steganopus tricolor	Wilson's phalarope	0	[1]			1	[2]	1	[2]	1	[2]
Sterna hirundo	Common tern	0.8	[1]			1	[4]	0.6681	[191,192]		
Strix aluco	Tawny owl	0.7	[1]			0	[4]	0.347	[193]	0.4229	[193]
Sturnus unicolor	Spotless starling	15.7	[1]	1	[4]	0	[4]				
Sturnus vulgaris	Common starling	15.4	[1]	1	[4]	0	[4]	0.5	[194]		

Sula dactylatra	Masked booby	0	[1]			1	[195]				
Sula granti	Nazca booby	0	[1]			1	[196]				
Sula nebouxii	Blue-footed booby	6.9	[1]			1	[197]				
Sula sula	Red-footed booby	0	[1]	1	[198]	1	[198]				
Tachycineta albilinea	Mangrove swallow	15.5	[1]					0.4793	[199]		
Tachycineta bicolor	Tree swallow	48	[1]	1	[2]	0	[2]	0.4313	[200–203]	0.375	[200]
Tachycineta leucorrhoa	White rumped swallow	56.4	[1]	1	[204]	0	[204]	0.4054			
Tachycineta meyeni	Chilean swallow	6.8	[205]			1	[206]				
Taeniopygia guttata	Zebra finch	1.8	[1]	1	[207]	1	[208]				
Thalassarche cauta	Shy albatross	6.9	[1]			1	[209]	0.5*	[210]	0.5102	[210]
Thalassarche chrysostoma	Grey-headed albatross	7.2	[1]			1	[211]	0.5044	[212]		
Thalassarche melanophrys	Black-browed albatross	5.7	[1]			1	[211]	0.5386	[212]		
Thalassoica antarctica	Antarctic petrel	7.3	[1]			1	[213]				
Thamnophilus atrinucha	Black-crowned antshrike	3.4	[1]	1	[2]	1	[2]				
Thryothorus ludovicianus	Carolina wren	0	[1]	1	[2]	0	[2]	0.5*	[214]		
Thryothorus pleurostictus	Banded wren	4.5	[1]	1	[215]	0	[215]				
Thryothorus rufalbus	Rufous-and-white wren	1.9	[1]	1	[216]	0	[216]				
Tockus monteiri	Monteiro's hornbill	0	[1]			1	[217]	0.5811	[218]		
Troglodytes aedon	House wren	14.1	[1]	1	[2]	0	[2]				
Troglodytes troglodytes	Eurasian wren	16.3	[1]	1	[4]	0	[4]				
Turdus albicollis	White-necked thrush	18.2	[1]								
Turdus grayi	Clay-colored robin	37.8	[1]	0	[219]	0	[219]	0.4211	[220]		
Turdus migratorius	American robin	48.1	[1]	0	[221]	0	[221]	0.4651	[222]		
Tyrannus forficatus	Scissor-tailed flycatchers	48.8	[1]	0	[223]	0	[223]	0.136	[224]		

Tyrannus tyrannus	Eastern kingbird	46	[1]	0	[2]	0	[2]	0.4488	[225–227]		
Tyto alba	Barn owl	0.7	[1]			0	[4]	0.64	[228]	0.3787	[228]
Upupa epops	Eurasian hoopoe	5	[1]	1	[4]	0	[4]	0.5938	[229]	0.6439	[229]
Uria lomvia	Thick-billed murres	7.4	[1]			1	[4]	0.5568	[230]		
Uria aalge	Common murres	7.8	[1]			1	[4]				
Vermivora chrysoptera	Golden-winged warbler	38.7	[1]	0	[231]	0	[231]				
Vireo griseus	White-eyed vireo	2	[1]	1	[2]	1	[2]	0.65	[232]		
Vireo olivaceus	Red-eyed vireo	57.9	[1]	0	[2]	0	[2]				
Vireo solitarius	Blue-headed vireo	2.7	[1]	1	[2]	1	[2]	0.516	[233]		
Volatinia jacarina	Blue-black grassquits	23.7	[1]	1	[234]	0	[234]				
Wilsonia citrina	Hooded warbler	26.7	[1]	0	[235]	0	[235]				
Zonotrichia albicollis	White-throated sparrow	15.6	[1]	0	[2]	0	[2]				
Zonotrichia capensis	Rufous-collared sparrow	46.8	[1]	0	[236]	0	[236]	0.1921	[237]		
Zonotrichia leucophrys	White-crowned sparrow	38.1	[1]	0	[2]	0	[2]	0.3822	[238,239]		
Zosterops lateralis	Capricorn silvereye	0	[1]	1	[240]	1	[240]				

References

1. Brouwer L, Griffith SC. 2019 Extra-pair paternity in birds. *Mol. Ecol.* **28**, 4864–4882.
2. Del Hoyo J, Del Hoyo J, Elliott A, Sargatal J. 1992 *Handbook of the birds of the world*. Lynx edicions Barcelona.
3. Green DJ. 2002 Pair bond duration influences paternal provisioning and the primary sex ratio of brown thornbill broods. *Anim. Behav.* **64**, 791–800.
4. Cramp S. 1977 *The Birds of the Western Palearctic*. Oxford: Oxford Univ. Press.
5. HOP H, Kleindorfer S, Ille R, Dittami J. 1995 Prey abundance and male parental behaviour in Acrocephalus warblers. *Ibis* **137**, 490–496.

6. Sejberg D, Bensch S, Hasselquist D. 2000 Nestling provisioning in polygynous great reed warblers (Acrocephalus arundinaceus): do males bring larger prey to compensate for fewer nest visits? *Behav. Ecol. Sociobiol.* **47**, 213–219.

7. Hamao S, Saito DS. 2005 Extrapair fertilization in the black-browed reed warbler (Acrocephalus bistrigiceps): effects on mating status and nesting cycle of cuckolded and cuckolder males. *The Auk* **122**, 1086–1096.

8. Buchanan KL, Catchpole CK. 2000 Song as an indicator of male parental effort in the sedge warbler. *Proc. R. Soc. Lond. B Biol. Sci.* **267**, 321–326. (doi:10.1098/rspb.2000.1003)

9. Hoi H, Krištofík J, Darolová A. 2013 Experimentally Simulating Paternity Uncertainty: Immediate and Long-Term Responses of Male and Female Reed Warblers Acrocephalus scirpaceus. *PLOS ONE* **8**, e62541. (doi:10.1371/journal.pone.0062541)

10. Danner JE, Small DM, Ryder TB, Lohr B, Masters BS, Gill DE, Fleischer RC. 2018 Temporal patterns of extra-pair paternity in a population of Grasshopper Sparrows (Ammodramus savannarum) in Maryland. *Wilson J. Ornithol.* **130**, 40–51.

11. Harrison HH. 1979 *A Field Guide to Western Birds' Nests: Of 520 Species Found Breeding in the United States West of the Mississippi River*. Houghton Mifflin Harcourt.

12. Adler J, Ritchison G. 2011 Provisioning behavior of male and female Grasshopper Sparrows. *Wilson J. Ornithol.* **123**, 515–520.

13. Rauter C, Brodmann P, Reyer H. 2000 Provisioning behaviour in relation to food availability and nestling food demand in the Water Pipit Anthus spinoletta. *Ardea* **88**.

14. Poblete Y, Botero-Delgadillo E, Espíndola-Hernández P, Südel G, Vásquez RA. 2021 Female extra-pair behavior is not associated with reduced paternal care in Thorn-tailed Rayadito. *Ecol. Evol.* **11**, 3065–3071.

15. Espíndola-Hernández P, Castaño-Villa GJ, Vásquez RA, Quirici V. 2017 Sex-specific provisioning of nutritious food items in relation to brood sex ratios in a non-dimorphic bird. *Behav. Ecol. Sociobiol.* **71**, 1–8.

16. Carere C, Alleva E. 1998 Sex differences in parental care in the common swift (Apus apus): effect of brood size and nestling age. *Can. J. Zool.* **76**, 1382–1387.

17. Margalida A, González LM, Sánchez R, Oria J, Prada L. 2007 Parental behaviour of Spanish Imperial Eagles Aquila adalberti: sexual differences in a moderately dimorphic raptor. *Bird Study* **54**, 112–119.

18. Wiley JW, Wiley BN. 1981 Breeding season ecology and behavior of Ridgway's Hawk (Buteo ridgwayi). *The Condor* **83**, 132–151.

19. Fitzner RE. 1980 Behavioral ecology of the Swainson's Hawk (Buteo swainsoni) in Washington.

20. Navarro J, González-Solís J, Viscor G. 2007 Nutritional and feeding ecology in Cory's shearwater Calonectris diomedea during breeding. *Mar. Ecol. Prog. Ser.* **351**, 261–271.

21. Granadeiro JP, Nunes M, Silva MC, Furness RW. 1998 Flexible foraging strategy of Cory's shearwater, Calonectris diomedea, during the chick-rearing period. *Anim. Behav.* **56**, 1169–1176.

22. Filliater TS, Breitwisch R. 1997 Nestling Provisioning by the Extremely Dichromatic Northern Cardinal. *Wilson Bull.* **109**, 145–153.

23. Linville SU, Breitwisch R, Schilling AJ. 1998 Plumage brightness as an indicator of parental care in northern cardinals. *Anim. Behav.* **55**, 119–127. (doi:10.1006/anbe.1997.0595)

24. Schnitzer J, Exnerová A, Poláková R, Vinkler M, Tomášek O, Munclinger P, Albrecht T. 2014 Male ornamentation and within-pair paternity are not associated with male provisioning rates in scarlet rosefinches Carpodacus erythrinus. *Acta Ethologica* **17**, 89–97.

25. Björklund M. 1990 Mate choice is not important for female reproductive success in the common rosefinch (Carpodacus erythrinus). *The Auk* **107**, 35–44.

26. McGraw KJ, Nolan PM, Stoehr AM, Hill GE. 2001 Intersexual differences in age-specific parental effort in the house finch (Carpodacus mexicanus). *Etologia* **9**, 35–41.

27. Stoehr AM, Hill GE. 2000 Testosterone and the allocation of reproductive effort in male house finches (Carpodacus mexicanus). *Behav. Ecol. Sociobiol.* **48**, 407–411.

28. Phillips RA. 2002 Trios of brown skuas at Bird Island, South Georgia: incidence and composition. *The Condor* **104**, 694–697.

29. Young EC. 1963 The breeding behaviour of the south polar skua Catharacta maccormicki. *Ibis* **105**, 203–233.

30. Maurer G. 2008 Who cares? Males provide most parental care in a monogamous nesting cuckoo. *Ethology* **114**, 540–547.

31. Taplin A, Beurteaux Y. 1992 Aspects of the breeding biology of the Pheasant Coucal Centropus phasianinus. *Emu* **92**, 141–146.

32. Cairns DK. 1987 The ecology and energetics of chick provisioning by Black Guillemots. *The Condor* **89**, 627–635.

33. St Clair JJ, Herrmann P, Woods RW, Székely T. 2010 Female-biased incubation and strong diel sex-roles in the Two-banded Plover Charadrius falklandicus. *J. Ornithol.* **151**, 811–816.

34. Page GW, Stenzel LE, Warriner JS, Warriner JC, Paton PW. 2020 Snowy Plover (Charadrius nivosus), version 1.0. *Birds World* (doi:10.2173/bow.snoplo5.01)

35. Wiersma P, Kirwan GM. 2020 Madagascar Plover (Charadrius thoracicus), version 1.0. *Birds World* (doi:10.2173/bow.madplo1.01)

36. Jónsson JE, Ryder JP, Alisauskas RT. 2020 Ross's Goose (Anser rossii), version 1.0. *Birds World* (doi:10.2173/bow.rosgoo.01)

37. Cuthbert NL. 1954 A nesting study of the Black Tern in Michigan. *The Auk* , 36–63.

38. Arrieta RS, Campagna L, Mahler B, Lovette I, Llambías PE. 2022 Local male breeding density affects extra-pair paternity in a south temperate population of grass wrens Cistothorus platensis. *J. Avian Biol.* **2022**, e02887.

39. Herkert JR, Kroodsma DE, Gibbs JP. 2021 Grass Wren (Cistothorus platensis), version 1.1. *Birds World* (doi:10.2173/bow.sedwre.01.1)

40. Burns JT. 1982 Nests, territories, and reproduction of Sedge Wrens (Cistothorus platensis). *Wilson Bull.* , 338–349.

41. Avilés JM, Parejo D, Rodríguez J. 2011 Parental favouritism strategies in the asynchronously hatching European Roller (Coracias garrulus). *Behav. Ecol. Sociobiol.* **65**, 1549–1557.

42. Expósito-Granados M, Parejo D, Avilés JM. 2016 Sex-Specific Parental Care in Response to Predation Risk in the European Roller, Coracias garrulus. *Ethology* **122**, 72–79. (doi:10.1111/eth.12444)

43. Knief U, Bossu CM, Wolf JB. 2020 Extra-pair paternity as a strategy to reduce the costs of heterospecific reproduction? Insights from the crow hybrid zone. *J. Evol. Biol.* **33**, 727–733.

44. Masello JF, Quillfeldt P. 2004 Are haematological parameters related to body condition, ornamentation and breeding success in wild burrowing parrots Cyanoliseus patagonus? *J. Avian Biol.* **35**, 445–454.

45. Taborsky M, Brugger C. 1994 The functional significance of shared incubation. *J. Für Ornithol.* **135**, 273.

46. Whittingham LA, Lifjeld JT. 1995 High paternal investment in unrelated young: extra-pair paternity and male parental care in house martins. *Behav. Ecol. Sociobiol.* **37**, 103–108. (doi:10.1007/BF00164155)

47. Westerterp KR, Bryant DM. 1984 Energetics of free existence in swallows and martins (hirundinidae) during breeding: a comparative study using doubly labeled water. *Oecologia* **62**, 376–381. (doi:10.1007/BF00384270)

48. Winkler H, Michalek K. 2001 Parental care and parentage in monogamous great spotted woodpeckers (Picoides major) and middle spotted woodpeckers (Picoides medius). *Behaviour* **138**, 1259–1285.

49. Harding KC. 1931 Nesting habits of the black throated blue warbler. *The Auk* , 512–522.

50. Stodola KW, Linder ET, Buehler DA, Franzreb KE, Kim DH, Cooper RJ. 2010 Relative influence of male and female care in determining nestling mass in a migratory songbird. *J. Avian Biol.* **41**, 515–522.

51. Goodbred CO, Holmes RT. 1996 Factors Affecting Food Provisioning of Nestling Black-Throated Blue Warblers. *Wilson Bull.* **108**, 467–479.

52. Lawrence LDK. 1948 Comparative study of the nesting behavior of Chestnut-sided and Nashville warblers. *The Auk* , 204–219.

53. Schrantz FG. 1943 Nest life of the eastern yellow warbler. *The Auk* , 367–387.

54. Yezerinac SM, Weatherhead PJ, Boag PT. 1996 Cuckoldry and lack of parentage-dependent paternal care in yellow warblers: a cost–benefit approach. *Anim. Behav.* **52**, 821–832.

55. Croxall JP, Ricketts C. 1983 Energy costs of incubation in the Wandering Albatross Diomedea exulans. *Ibis* **125**, 33–39.

56. Berrow SD, Croxall JP. 2001 Provisioning rate and attendance patterns of wandering albatrosses at Bird Island, South Georgia. *The Condor* **103**, 230–239.

57. White EM, Perlut NG, Travis SE, Strong AM. 2021 Microsatellite markers yield new insight into extra-pair paternity in Bobolinks (Dolichonyx oryzivorus). *Wilson J. Ornithol.* **133**, 476–483.

58. Dolby AS, Clarkson CE, Haas ET, Miller JK, Havens LE, Cox BK. 2005 Do song-phrase production rate and song versatility honestly communicate male parental quality in the Gray Catbird? *J. Field Ornithol.* **76**, 287–292.

59. Stutchbury BJ, Morton ES, Woolfenden B. 2007 Comparison of the mating systems and breeding behavior of a resident and a migratory tropical flycatcher. *J. Field Ornithol.* **78**, 40–49.

60. Sundberg J, Larsson C. 1994 Male coloration as an indicator of parental quality in the yellowhammer, Emberiza citrinella. *Anim. Behav.* **48**, 885–892.

61. Suter SM, Bielańska J, Röthlin-Spillmann S, Strambini L, Meyer DR. 2009 The cost of infidelity to female reed buntings. *Behav. Ecol.* **20**, 601–608. (doi:10.1093/beheco/arp037)

62. Bouwman KM, Lessells CM, Komdeur J. 2005 Male reed buntings do not adjust parental effort in relation to extrapair paternity. *Behav. Ecol.* **16**, 499–506. (doi:10.1093/beheco/ari021)

63. Walkinshaw LH. 1966 Summer observations of the Least Flycatcher in Michigan. *Jack-Pine Warbler* **44**, ll.

64. McCabe RA. 1993 *The little green bird: ecology of the willow flycatcher*. Rusty Rock Press, Department of Wildlife Ecology [University of Wisconsin].

65. Allen MC, Napoli MM, Sheehan J, Master TL, Pyle P, Whitehead DR, Taylor T. 2020 Acadian Flycatcher (Empidonax virescens), version 1.0. *Birds World* (doi:10.2173/bow.acafly.01)

66. Gwiazdowska A, Karpińska O, Kamionka-Kanclerska K, Rowiński P, Panagiotopoulou H, Pomorski JJ, Broughton RK, da Silva LF, Rutkowski R. 2021 First microsatellite markers for the European Robin (Erithacus rubecula) and their application in analysis of parentage and genetic diversity. *Sci. Rep.* **11**, 1–9.

67. Brazill-Boast J, Griffith SC, Pryke SR. 2013 Morph-dependent resource acquisition and fitness in a polymorphic bird. *Evol. Ecol.* **27**, 1189–1198. (doi:10.1007/s10682-013-9651-1)

68. Negro, J. J., Donazar, J. A., & Hiraldo, F. 1992 Kleptoparasitism and cannibalism in a colony of Lesser Kestrels (Falco naumanni). *J. Raptor Res.* , 225–228.

69. Olsen P, Doyle V, Boulet M. 1998 Variation in Male Provisioning in Relation to Brood Size of Peregrine Falcons Falco peregrinus. *Emu* **98**, 297–304. (doi:10.1071/mu98041)

70. Smallwood JA, Bird DM. 2020 American Kestrel (Falco sparverius), version 1.0. *Birds World* (doi:10.2173/bow.amekes.01)

71. Liébana MS, Sarasola JH, Bó MS. 2009 Parental Care and Behavior of Breeding American Kestrels (Falco sparverius) in Central Argentina. *J. Raptor Res.* **43**, 338–344. (doi:10.3356/JRR-08-82.1)

72. Smith DG, Wilson CR, Frost HH. 1972 The Biology of the American Kestrel in Central Utah. *Southwest. Nat.* **17**, 73–83. (doi:10.2307/3669841)

73. Masman D, Daan S, Dijkstra C. 1988 Time Allocation in the Kestrel (Falco tinnunculus), and the Principle of Energy Minimization. *J. Anim. Ecol.* **57**, 411–432. (doi:10.2307/4914)

74. Szász E, Markó G, Hegyi G, Török J, Garamszegi LZ, Rosivall B. 2019 Nest-site defence aggression during courtship does not predict nestling provisioning in male collared flycatchers. *Behav. Ecol. Sociobiol.* **73**, 62. (doi:10.1007/s00265-019-2672-1)

75. Part T, Gustafsson L, Moreno J. 1992 'Terminal Investment' and a Sexual Conflict in the Collared Flycatcher (Ficedula albicollis). *Am. Nat.* **140**, 868–882.

76. Cauchard L, Macqueen EI, Lilley R, Bize P, Doligez B. 2021 Inter-individual variation in provisioning rate, prey size and number, and links to total prey biomass delivered to nestlings in the Collared Flycatcher (Ficedula albicollis). *Avian Res.* **12**, 15. (doi:10.1186/s40657-021-00247-8)

77. Lifield JT, Slagsvold T. 1990 Manipulations of male parental investment in polygynous pied flycatchers, Ficedula hypoleuca. *Behav. Ecol.* **1**, 48–54. (doi:10.1093/beheco/1.1.48)

78. Alatalo RV, Gottlander K, Lundberg A. 1988 Conflict or Cooperation between Parents in Feeding Nestlings in the Pied Flycatcher Ficedula hypoleuca. *Ornis Scand. Scand. J. Ornithol.* **19**, 31–34. (doi:10.2307/3676524)

79. Mitrus C, Mitrus J, Sikora M. 2010 Sex Differences in the Rate of Food Provisioning to Nestlings Red-Breasted Flycatchers (Ficedula parva). *Ann. Zool. Fenn.* **47**, 144–148. (doi:10.5735/086.047.0207)

80. ZANTHOPYGIA F. 2010 Breeding biology of the yellow-rumped flycatcher Ficedula zanthopygia in northeast China. *Ardeola* **57**, 103–110.

81. Clement P, de Juana E. 2020 Yellow-rumped Flycatcher (Ficedula zanthopygia), version 1.0. *Birds World* (doi:10.2173/bow.korfly1.01)

82. Gauger Metz VH, Schreiber EA. 2020 Great Frigatebird (Fregata minor), version 1.0. *Birds World* (doi:10.2173/bow.grefri.01)

83. Diniz P, Macedo RH, Webster MS. 2019 Duetting correlates with territory quality and reproductive success in a suboscine bird with low extra-pair paternity. *Auk Ornithol. Adv.* **136**, uky004.

84. Massoni V, Reboreda JC, López GC, Aldatz MF. 2012 High coordination and equitable parental effort in the Rufous Hornero. *The Condor* **114**, 564–570.

85. Leonard ML, Horn AG, Eden SF. 1988 Parent-offspring aggression in moorhens. *Behav. Ecol. Sociobiol.* **23**, 265–270.

86. Stewart RE. 1953 A life history study of the Yellow-throat. *Wilson Bull.* **65**, 99–115.

87. Mitchell DP, Dunn P, Whittingham LA, Freeman-Gallant CR. 2007 Attractive males provide less parental care in two populations of the common yellowthroat. *Anim. Behav.* (doi:10.1016/j.anbehav.2006.07.006)

88. Bojarska K *et al.* 2018 Mating system and extra-pair paternity in the Fan-tailed Gerygone Gerygone flavolateralis in relation to parasitism by the Shining Bronze-cuckoo Chalcites lucidus. *Plos One* **13**, e0194059.

89. Hall ML. 1999 The importance of pair duration and biparental care to reproductive success in the monogamous Australian magpie-lark. *Aust. J. Zool.* **47**, 439–454. (doi:10.1071/zo99037)

90. In press. Sandhill Crane - Antigone canadensis - Birds of the World. See https://birdsoftheworld.org/bow/species/sancra/cur/introduction (accessed on 26 June 2022).

91. Bartsch C, Weiss M, Kipper S. 2015 Multiple song features are related to paternal effort in common nightingales. *BMC Evol. Biol.* **15**, 115. (doi:10.1186/s12862-015-0390-5)

92. Rymešová D, Pavlíček D, Kirner J, Mráz J, Papoušek I, Literák I. 2020 Parentage analysis in the White-tailed Eagle Haliaeetus albicilla: are moulted feathers from nest sites a reliable source of parental DNA? *Acta Ornithol.* **55**, 41–52.

93. Maguire SE, Safran RJ. 2010 Morphological and genetic predictors of parental care in the North American barn swallow Hirundo rustica erythrogaster. *J. Avian Biol.* **41**, 74–82. (doi:10.1111/j.1600-048X.2009.04806.x)

94. Evans M, Gow E, Roth RR, Johnson MS, Underwood TJ. 2020 Wood Thrush (Hylocichla mustelina), version 1.0. *Birds World* (doi:10.2173/bow.woothr.01)

95. Carboneras C, Kirwan GM. 2020 Blue Duck (Hymenolaimus malacorhynchos), version 1.0. *Birds World* (doi:10.2173/bow.bluduc1.01)

96. In press. Yellow-breasted Chat - Icteria virens - Birds of the World. See https://birdsoftheworld.org/bow/species/yebcha/cur/introduction (accessed on 26 June 2022).

97. Cooper SW, Ritchison G. 2005 Nestling provisioning by male and female Yellow-breasted Chats: no relationships between morphology and parental care. *J. Field Ornithol.* **76**, 298–302. (doi:10.1648/0273-8570-76.3.298)

98. Bent AC. 1958 Life histories of North American blackbirds, orioles, tanagers, and allies. *Bull. U. S. Natl. Mus.*

99. Barnhill RA, Weyer D, Young WF, Smith KG, James DA. 2005 Breeding biology of Jabirus (Jabiru mycteria) in Belize. *Wilson Bull.* **117**, 142–153.

100. Giesen KM, Braun CE. 1979 Nesting behavior of female White-tailed Ptarmigan in Colorado. *The Condor* **81**, 215–217.

101. Yamagishi S, Saito M. 1985 Function of courtship feeding in the Bull-headed Shrike, Lanius bucephalus. *J. Ethol.* **3**, 113–121.

102. Yosef R, International Shrike Working Group I. 2020 Bull-headed Shrike (Lanius bucephalus), version 1.0. *Birds World* (doi:10.2173/bow.buhshr1.01)

103. Nishida Y, Takagi M. 2018 Song performance is a condition-dependent dynamic trait honestly indicating the quality of paternal care in the bull-headed shrike. *J. Avian Biol.* **49**, e01794. (doi:10.1111/jav.01794)

104. Pierotti R. 1976 Sex roles, social structure, and the role of the environment in the Western Gull. *Unpubl. Masters Thesis Calif. State Univ. Sacram.*

105. Pierotti R. 1981 Male and Female Parental Roles in the Western Gull under Different Environmental Conditions. *The Auk* **98**, 532–549.

106. Pletschet SM, Kelly JF. 1990 Breeding Biology and Nesting Success of Palila. *The Condor* **92**, 1012–1021. (doi:10.2307/1368737)

107. Laskemoen T, Fossøy F, Rudolfsen G, Lifjeld JT. 2008 Age-related variation in primary sexual characters in a passerine with male age-related fertilization success, the bluethroat Luscinia svecica. *J. Avian Biol.* **39**, 322–328.

108. Smiseth PT, Amundsen T. 2000 Does female plumage coloration signal parental quality? A male removal experiment with the bluethroat (Luscinia s. svecica). *Behav. Ecol. Sociobiol.* **47**, 205–212. (doi:10.1007/s002650050657)

109. Courter JR. 2017 Provisioning Behavior of Male and Female Eastern Screech-Owls During the Post-Brooding Period. *Am. Midl. Nat.* **177**, 69–74. (doi:10.1674/0003-0031-177.1.69)

110. Nordlund CA, Barber CA. 2005 Parental Provisioning in Melospiza melodia (Song Sparrows). *Northeast. Nat.* **12**, 425–432. (doi:10.1656/1092-6194(2005)012[0425:PPIMMS]2.0.CO;2)

111. Grunst ML, Rotenberry JT, Grunst AS. 2016 Elevating perceived predation risk modifies the relationship between parental effort and song complexity in the song sparrow Melospiza melodia. *J. Avian Biol.* **47**, 57–68. (doi:10.1111/jav.00758)

112. Gow EA, Arcese P, Dagenais D, Sardell RJ, Wilson S, Reid JM. 2019 Testing predictions of inclusive fitness theory in inbreeding relatives with biparental care. *Proc. R. Soc. B Biol. Sci.* **286**, 20191933. (doi:10.1098/rspb.2019.1933)

113. Hartley IR, Shepherd M. 1994 Female reproductive success, provisioning of nestlings and polygyny in corn buntings. *Anim. Behav.* **48**, 717–725. (doi:10.1006/anbe.1994.1290)

114. Clark ES. 1980 The attentiveness and time budget of a pair of nesting Wood Storks. In *Proceedings of the Colonial Waterbird Group*, pp. 204–215. JSTOR.

115. Eberhard JR. 1998 Breeding biology of the Monk Parakeet. *Wilson Bull.* , 463–473.

116. Goldstein H, Yom-Tov Y. 1988 Breeding biology of the orange-tufted sunbird in Israel. *Ardea* **76**, 169–174.

117. Gladbach A, Braun C, Nordt A, Peter H-U, Quillfeldt P. 2009 Chick provisioning and nest attendance of male and female Wilson's storm petrels Oceanites oceanicus. *Polar Biol.* **32**, 1315–1321.

118. Mauck RA, Zangmeister JL, Cerchiara JC, Huntington CE, Haussmann MF. 2011 Male-biased reproductive effort in a long-lived seabird. *Evol. Ecol. Res.* **13**, 19–33.

119. Moreno J. 1984 Parental care of fledged young, division of labor, and the development of foraging techniques in the Northern Wheatear (Oenanthe oenanthe L.). *The Auk* **101**, 741–752.

120. Moreno J. 1987 Parental care in the wheatear Oenanthe oenanthe: effects of nestling age and brood size. *Ornis Scand.* , 291–301.

121. Murakami R, Sawada A, Ono H, Takagi M. 2022 The effect of experience on parental role division in Ryukyu Scops Owl Otus elegans. *Ornithol. Sci.* **21**, 35–44.

122. Weimerskirch H, Chastel O, Ackermann L. 1995 Adjustment of parental effort to manipulated foraging ability in a pelagic seabird, the thin-billed prion Pachyptila belcheri. *Behav. Ecol. Sociobiol.* **36**, 11–16. (doi:10.1007/BF00175723)

123. Quillfeldt P, Strange IJ, Segelbacher G, Masello JF. 2007 Male and female contributions to provisioning rates of thin-billed prions, Pachyptila belcheri, in the South Atlantic. *J. Ornithol.* **148**, 367–372. (doi:10.1007/s10336-007-0138-0)

124. Hoi H, Hoi-Leitner M. 1997 An alternative route to coloniality in the bearded tit: females pursue extra-pair fertilizations. *Behav. Ecol.* **8**, 113–119. (doi:10.1093/beheco/8.2.113)

125. Lee J-W, Kim H-Y, Hatchwell BJ. 2010 Parental provisioning behaviour in a flock-living passerine, the Vinous-throated Parrotbill Paradoxornis webbianus. *J. Ornithol.* **151**, 483–490.

126. Brewer R. 1961 Comparative notes on the life history of the Carolina Chickadee. *Wilson Bull.* , 348–373.

127. Arct A, Drobniak SM, Mellinger S, Martyka R, Gustafsson L, Cichoń M. 2022 Extra-pair paternity in Blue Tits (Cyanistes caeruleus) depends on the combination of social partners' age. *Ibis* **164**, 388–395. (doi:10.1111/ibi.13022)

128. Slagsvold T, Lifjeld JT. 1990 Influence of Male and Female Quality on Clutch Size in Tits (Parus Spp.). *Ecology* **71**, 1258–1266. (doi:10.2307/1938263)

129. Serrano-Davies E, Sanz JJ. 2017 Habitat structure modulates nestling diet composition and fitness of Blue Tits *Cyanistes caeruleus* in the Mediterranean region. *Bird Study* **64**, 295–305. (doi:10.1080/00063657.2017.1357678)

130. GarcÍa-Navas V, Sanz JJ. 2012 Environmental and Within-Nest Factors Influencing Nestling-Feeding Patterns of Mediterranean Blue Tits (Cyanistes Caeruleus). *The Condor* **114**, 612–621. (doi:10.1525/cond.2012.110120)

131. Banbura, J., Perret, P., Blondel, J., Sauvages, A. N. N. E., Galan, M. J., & Lambrechts, M. M. 2001 Sex differences in parental care in a Corsican Blue Tit Parus caeruleus population. *Ardea* **89**, 517–526.

132. Remeš V, Matysioková B. 2013 More ornamented females produce higher-quality offspring in a socially monogamous bird: an experimental study in the great tit (Parus major). *Front. Zool.* **10**, 14. (doi:10.1186/1742-9994-10-14)

133. Both C, Michler S, Tinbergen J, Komdeur J, Velde M van der, Bleeker M. 2010 Parental provisioning in relation to offspring sex and sex ratio in the great tit (Parus major). *Behaviour* **147**, 1355–1378. (doi:10.1163/000579510X517208)

134. Sanz JJ, Tinbergen JM. 1999 Energy expenditure, nestling age, and brood size: an experimental study of parental behavior in the great tit Parus major. *Behav. Ecol.* **10**, 598–606. (doi:10.1093/beheco/10.5.598)

135. Verhulst S, Tinbergen JM. 1997 Clutch size and parental effort in the Great Tit Parus major. *Ardea -Wagening.-* **85**, 111–126.

136. Voltura KM, Schwagmeyer PL, Mock DW. 2002 Parental Feeding Rates in the House Sparrow, Passer domesticus: Are Larger-Badged Males Better Fathers? *Ethology* **108**, 1011–1022. (doi:10.1046/j.1439-0310.2002.00831.x)

137. Ringsby TH, Berge T, Saether B-E, Jensen H. 2009 Reproductive success and individual variation in feeding frequency of House Sparrows (Passer domesticus). *J. Ornithol.* **150**, 469–481. (doi:10.1007/s10336-008-0365-z)

138. Kopisch AD, Schwagmeyer PL, Mock DW. 2005 Individual Consistency in Parental Effort Across Multiple Stages of Care in the House Sparrow, Passer domesticus. *Ethology* **111**, 1062–1070. (doi:10.1111/j.1439-0310.2005.01137.x)

139. Dugas MB. 2009 House sparrow, Passer domesticus, parents preferentially feed nestlings with mouth colours that appear carotenoid-rich. *Anim. Behav.* **78**, 767–772. (doi:10.1016/j.anbehav.2009.07.009)

140. Perlut NG, Kelly LM, Zalik NJ, Strong AM. 2012 Male savannah sparrows provide less parental care with increasing paternity loss. *Northeast. Nat.* **19**, 335–344.

141. Weatherhead PJ. 1979 Do Savannah Sparrows Commit the Concorde Fallacy? *Behav. Ecol. Sociobiol.* **5**, 373–381.

142. Wheelwright NathanielT, Schultz CherylB, Hodum PeterJ. 1992 Polygyny and male parental care in Savannah sparrows: effects on female fitness. *Behav. Ecol. Sociobiol.* **31**. (doi:10.1007/BF00171683)

143. Keyser AJ, Hill GE. 2000 Structurally based plumage coloration is an honest signal of quality in male blue grosbeaks. *Behav. Ecol.* **11**, 202–209. (doi:10.1093/beheco/11.2.202)

144. Sorensen MC, Strickland D, Freeman NE, Fuirst M, Sutton AO, Norris DR. 2022 Early-life experience shapes patterns of senescence in a food-caching passerine. *Biol. Lett.* **18**, 20210532.

145. Strickland D, Ouellet H. 1993 *Gray Jay: Perisoreus Canadensis*. American Ornithologists' Union.

146. Heber S. 2013 Anecdotal evidence of interspecific parental care: Feeding and brooding of robin (Petroica australis) nestlings by a female bellbird (Anthornis melanura). *Notornis* **60**, 186–187.

147. García-Navas V, Rincón AG del, Ferrer ES, Fathi H. 2013 Mating strategies, parental investment and mutual ornamentation in Iberian Rock Sparrows (Petronia petronia). *Behaviour* **150**, 1641–1663. (doi:10.1163/1568539X-00003112)

148. Griggio M, Zanollo V, Hoi H. 2010 Female ornamentation, parental quality, and competitive ability in the rock sparrow. *J. Ethol.* **28**, 455–462. (doi:10.1007/s10164-010-0205-5)

149. Rand AL, Rand RM. 1943 Breeding notes on the Phainopepla. *The Auk* , 333–341.

150. Huyvaert KP, Anderson DJ, Parker PG. 2006 Mate opportunity hypothesis and extrapair paternity in waved albatrosses (Phoebastria irrorata). *The Auk* **123**, 524–536.

151. Draganoiu TI, Nagle L, Musseau R, Kreutzer M. 2005 Parental care and brood division in a songbird, the black redstart. *Behaviour* **142**, 1495–1514. (doi:10.1163/156853905774831828)

152. Kryštofková M, Exnerová A. 2006 Parental foraging strategies and feeding of nestlings in Common Redstart Phoenicurus phoenicurus. **83**, 10.

153. Clarke RH, Clarke MF. 1999 The social organization of a sexually dimorphic honeyeater: the Crescent Honeyeater Phylidonyris pyrrhoptera, at Wilsons Promontory, Victoria. *Aust. J. Ecol.* **24**, 644–654. (doi:10.1046/j.1442-9993.1999.00990.x)

154. Forstmeier W, Kuijper DP, Leisler B. 2001 Polygyny in the dusky warbler, Phylloscopus fuscatus: the importance of female qualities. *Anim. Behav.* **62**, 1097–1108.

155. Temrin H, Brodin A, Åkerström O, Stenius S. 1997 Parental investment in monogamous pairs of Wood Warblers (Phylloscopus sibilatrix). *J. Für Ornithol.* **138**, 93–101. (doi:10.1007/BF01651655)

156. Bj0rnstad G, Lifjeld JT. 1996 Male parental care promotes early fledging in an open-nester, the Willow Warbler Phylloscopus trochilus. *Ibis* **138**, 229–235. (doi:10.1111/j.1474-919X.1996.tb04333.x)

157. Pechacek P, Michalek KG, Winkler H, Blomqvist D. 2006 Classical polyandry found in the three-toed woodpecker Picoides tridactylus. *J. Ornithol.* **147**, 112–114. (doi:10.1007/s10336-005-0026-4)

158. McKay JE, Murphy MT, Smith SB, Richardson JK. 2009 Fecal-Sac Ingestion by Spotted Towhees. *The Condor* **111**, 503–510. (doi:10.1525/cond.2009.080065)

159. Allen RP. 1942 *The roseate spoonbill*. National Audubon Society.

160. Eastwood JR, Berg ML, Ribot RF, Stokes HS, Martens JM, Buchanan KL, Walder K, Bennett AT. 2018 Pair fidelity in long-lived parrots: genetic and behavioural evidence from the Crimson Rosella (Platycercus elegans). *Emu-Austral Ornithol.* **118**, 369–374.

161. Lenz M. 1988 Crimson Rosellas' Platycercus elegans' nesting in buildings in Canberra. *Aust. Bird Watch.* **12**, 171–173.

162. Lyon BE, Montgomerie RD, Hamilton LD. 1987 Male parental care and monogamy in snow buntings. *Behav. Ecol. Sociobiol.* **20**, 377–382. (doi:10.1007/BF00300684)

163. Falconer CM, Mallory ML, Nol E. 2008 Breeding biology and provisioning of nestling snow buntings in the Canadian High Arctic. *Polar Biol.* **31**, 483–489. (doi:10.1007/s00300-007-0374-z)

164. Hofstad E, Espmark Y, Moksnes A, Haugan T, Ingebrigtsen M. 2002 The relationship between song performance and male quality in snow buntings (*Plectrophenax nivalis*). *Can. J. Zool.* **80**, 524–531. (doi:10.1139/z02-033)

165. Bonderud ES, Otter KA, Burg TM, Marini KLD, Reudink MW. 2018 Patterns of extra-pair paternity in mountain chickadees. *Ethology* **124**, 378–386. (doi:10.1111/eth.12747)

166. Wang J, Wei Y, Zhang L, Jiang Y, Li K, Wan D. 2021 High level of extra-pair paternity in the socially monogamous Marsh Tits (Poecile palustris). *Avian Res.* **12**, 69. (doi:10.1186/s40657-021-00304-2)

167. van Rooij E, Griffith S. 2011 Breeding ecology of an Australian estrildid, the Long-tailed Finch (Poephila acuticauda). *Emu* **111**, 297. (doi:10.1071/MU10092)

168. del Hoyo J, Collar N, Sharpe CJ. 2020 South Island Takahe (Porphyrio hochstetteri), version 1.0. *Birds World* (doi:10.2173/bow.takahe3.01)

169. Ding Z-F, Zhang C-L, Zhang W-S, Yuan Q-M, Wang L-W, Ren G, Li E, Hu H-J, Liang W. 2021 Determining the level of extra-pair paternity in yellow-bellied prinias, a socially monogamous passerine. *Zool. Res.* **42**, 108–115. (doi:10.24272/j.issn.2095-8137.2020.079)

170. Wagner RH, Schug MD, Morton ES. 1996 Confidence of paternity, actual paternity and parental effort by purple martins. *Anim. Behav.* **52**, 123–132. (doi:10.1006/anbe.1996.0158)

171. Carey MJ. 2011 Incubation routine, duration of foraging trips and regulation of body mass in Short-tailed Shearwaters (Ardenna tenuirostris). *Emu-Austral Ornithol.* **111**, 166–171.

172. de Mendonça Dantas GP, Gonzaga LG, da Silveira AS, Werle GB, da Cruz Piuco R, Petry MV. 2020 Extra-pair paternity and intraspecific brood parasitism in the Gentoo Penguin (Pygoscelis papua) on Elephant Island, Antarctica. *Polar Biol.* **43**, 851–859.

173. Westerterp KR, Bryant DM. 1984 Energetics of free existence in swallows and martins (hirundinidae) during breeding: a comparative study using doubly labeled water. *Oecologia* **62**, 376–381. (doi:10.1007/BF00384270)

174. Moe B, Langseth I, Fyhn M, Gabrielsen GW, Bech C. 2002 Changes in body condition in breeding kittiwakes Rissa tridactyla. *J. Avian Biol.* **33**, 225–234. (doi:10.1034/j.1600-048X.2002.330304.x)

175. Horn R, Ritchison G. 2017 Plumage Colouration and the Provisioning Behaviour of Male and Female Eastern Phoebes. *Avian Biol. Res.* **10**, 164–173. (doi:10.3184/175815617X14969254461387)

176. Conrad KF, Robertson RJ. 1993 Patterns of Parental Provisioning by Eastern Phoebes. *The Condor* **95**, 57–62. (doi:10.2307/1369386)

177. Sturm L. 1945 A study of the nesting activities of the American Redstart. *The Auk* , 189–206.

178. Omland KE, Sherry TW. 1994 Parental Care at Nests of Two Age Classes of Male American Redstart: Implications for Female Mate Choice. *The Condor* **96**, 606–613. (doi:10.2307/1369463)

179. Criddle N. 1927 Habits of the mountain bluebird in Manitoba. *Can. Field-Nat.* **41**, 40–44.

180. Morrison A, Flood NJ, Reudink MW. 2014 Reproductive correlates of plumage coloration of female Mountain Bluebirds. *J. Field Ornithol.* **85**, 168–179.

181. Johnson LS, Brubaker JL, Ostlind E, Balenger SL. 2007 Effect of altitude on male parental expenditure in Mountain Bluebirds (Sialia currucoides): are higher-altitude males more attentive fathers? *J. Ornithol.* **148**, 9–16.

182. Gowaty PA, Plissner JH. 2020 Eastern Bluebird (Sialia sialis), version 1.0. *Birds World* (doi:10.2173/bow.easblu.01)

183. Siefferman L, Hill GE. 2005 Male eastern bluebirds trade future ornamentation for current reproductive investment. *Biol. Lett.* **1**, 208–211. (doi:10.1098/rsbl.2004.0274)

184. Benítez Saldívar MJ, Miño CI, Massoni V. 2019 Genetic mating system, population genetics and effective size of Saffron Finches breeding in southern South America. *Genetica* **147**, 315–326.

185. Burnham H, Cruz-Bernate L. 2020 Parental investment does not directly affect reproductive success in the saffron finch. *J. Avian Biol.* **51**.

186. Palmerio AG, Massoni V. 2011 Parental care does not vary with age-dependent plumage in male Saffron Finches Sicalis flaveola. *Ibis* **153**, 421–424. (doi:10.1111/j.1474-919X.2011.01103.x)

187. Pagenkopf K, Wesotowski T. 2002 Do male Nuthatches (Sitta europaea) guard their mates? *J. Für Ornithol.* **143**, 145–154. (doi:10.1007/BF02465446)

188. Martínez I, Christie D, Jutglar F, Garcia E, Kirwan GM. 2020 Humboldt Penguin (Spheniscus humboldti), version 1.0. *Birds World* (doi:10.2173/bow.humpen1.01)

189. Marasco ACM, Morgante JS, Barrionuevo M, Frere E, de Mendonça Dantas GP. 2020 Molecular evidence of extra-pair paternity and intraspecific brood parasitism by the Magellanic Penguin (Spheniscus magellanicus). *J. Ornithol.* **161**, 125–135.

190. Carey M. 1990 Effects of brood size and nestling age on parental care by male field sparrows (Spizella pusilla). *The Auk* **107**, 580–586.

191. Wiggins DA, Morris RD. 1987 Parental care of the Common Tern Sterna hirundo. *Ibis* **129**, 533–540. (doi:10.1111/j.1474-919X.1987.tb08241.x)

192. Burness G, Morris R, Bruce J. 2011 Seasonal and annual variation in brood attendance, prey type delivered to chicks, and foraging patterns of male Common Terns (Sterna hirundo). *Can. J. Zool.* **72**, 1243–1251. (doi:10.1139/z94-166)

193. Sasvári L, Hegyi Z, Csörgõ T, Hahn I. 2000 Age-dependent diet change, parental care and reproductive costin tawny owls Strix aluco. *Acta Oecologica* **21**, 267–275.

194. Wright J, Cuthill I. 1990 Biparental care: short-term manipulation of partner contribution and brood size in the starling, Sturnus vulgaris. *Behav. Ecol.* **1**, 116–124.

195. Nelson S, Nelson B. 1978 *The Sulidae: gannets and boobies*. Oxford University Press, USA.

196. Milligan CL, Wood CM. 1987 Effects of Strenuous Activity on Intracellular and Extracellular Acid-Base Status and H^+ Exchange with the Environment in the Inactive, Benthic Starry Flounder Platichthys stellatus. *Physiol. Zool.* **60**, 37–53.

197. Hernández Díaz JA, Salazar Gómez EN. 2020 Blue-footed Booby (Sula nebouxii), version 1.0. *Birds World* (doi:10.2173/bow.bfoboo.01)

198. Amerson AB, Shelton PC. 1976 The Natural History of Johnston Atoll, Central Pacific Ocean.

199. Stager M, Lopresti E, Pratolongo FA, Ardia DR, Cooper CB, Iñigo-elias EE, Molina J, Taylor N, Winkler DW. 2012 Reproductive biology of a narrowly endemic Tachycineta swallow in dry, seasonal forest in coastal Peru. *Ornitol. Neotropical* **23**, 95–112.

200. McCarty JP. 2002 The Number of Visits to the Nest by Parents Is an Accurate Measure of Food Delivered to Nestlings in Tree Swallows (El número de visitas al nido por los padres es una medida exacta del alimento llevado a los pichones en Tachycineta bicolor). *J. Field Ornithol.* **73**, 9–14.

201. Lifjeld JT, Dunn PO, Robertson RJ, Boag PT. 1993 Extra-pair paternity in monogamous tree swallows. *Anim. Behav.* **45**, 213–229. (doi:10.1006/anbe.1993.1028)

202. Whittingham LA, Dunn PO, Clotfelter ED. 2003 Parental allocation of food to nestling tree swallows: the influence of nestling behaviour, sex and paternity. *Anim. Behav.* **65**, 1203–1210. (doi:10.1006/anbe.2003.2178)

203. Ardia DR. 2007 Site- and sex-level differences in adult feeding behaviour and its consequences to offspring quality in tree swallows (Tachycineta bicolor) following brood-size manipulation. *Can. J. Zool.* **85**, 847–854. (doi:10.1139/Z07-070)

204. Wischhoff U, Marques-Santos F, Ardia DR, Roper JJ. 2015 White-rumped swallows prospect while they are actively nesting. *J. Ethol.* **33**, 145–150.

205. Ferretti V, Liljesthröm M, López AS, Lovette IJ, Winkler DW. 2016 Extra-pair paternity in a population of Chilean Swallows breeding at 54 degrees south. *J. Field Ornithol.* **87**, 155–161.

206. Ospina EA, Cooper CB, Liljesthröm M, Ardia DR, Winkler DW. 2015 Biparental nest-attendance in Chilean Swallows (Tachycineta meyeni) breeding in Ushuaia, Argentina. *Emu-Austral Ornithol.* **115**, 76–79.

207. Payne RB. 2020 Zebra Finch (Taeniopygia guttata), version 1.0. *Birds World* (doi:10.2173/bow.zebfin2.01)

208. E. Gorman H, E. Arnold K, G. Nager R. 2005 Incubation effort in relation to male attractiveness in zebra finches Taeniopygia guttata. *J. Avian Biol.* **36**, 413–420.

209. Abbott CL, Double MC, Gales R, Cockburn A. 2006 Copulation behaviour and paternity in shy albatrosses (Thalassarche cauta). *J. Zool.* **270**, 628–635.

210. Hedd A, Gales R, Brothers N. 2002 Provisioning and growth rates of shy albatrosses at Albatross Island, Tasmania. *The Condor* **104**, 12–29.

211. Prince PA, Ricketts C, Thomas G. 1981 Weight loss in incubating albatrosses and its implications for their energy and food requirements. *The Condor* **83**, 238–242.

212. Huin N, PRlNCE PA, Briggs DR. 2000 Chick provisioning rates and growth in Blacklbrowed Albatross Diomedea melanophris and Grey-headed Albatross D. chrysostoma at Bird Island, South Georgia. *Ibis* **142**, 550–565.

213. LORENTSEN S-H, RØV N. 1995 Incubation and brooding performance of the Antarctic petrel Thalassoica antarctica at Svarthamaren, Dronning Maud Land. *Ibis* **137**, 345–351.

214. Neudorf DLH, Brodrick MJ, Cureton JC II. 2013 Parental Provisioning by Carolina Wrens. *Wilson J. Ornithol.* **125**, 179–184. (doi:10.1676/12-009.1)

215. Vehrencamp SL, Hall ML, Bohman ER, Depeine CD, Dalziell AH. 2007 Song matching, overlapping, and switching in the banded wren: the sender's perspective. *Behav. Ecol.* **18**, 849–859.

216. Douglas SB, Heath DD, Mennill DJ. 2012 Low levels of extra-pair paternity in a neotropical duetting songbird, the rufous-and-white wren (Thryothorus rufalbus). *The Condor* **114**, 393–400.

217. Klaassen M, Brenninkmeijer A, Boix-Hinzen C, Mendelsohn J. 2003 Fathers with highly demanding partners and offspring in a semidesert environment: Energetic aspects of the breeding system of Monteiro's Hornbills (Tockus monteiri) in Namibia. *The Auk* **120**, 866–873.

218. Kemp MI. 1972 A study of the biology of Monteiro's Hornbill. *Ann. Transvaal Mus.* **27**, 255–268.

219. Panwar P, Deshwal A, Kannan R, Collar N, Spencer AJ. 2021 Clay-colored Thrush (Turdus grayi), version 2.0. *Birds World* (doi:10.2173/bow.clcrob.02)

220. Sánchez NV, Vargas-Castro LE, Barrantes G. 2018 Nestling feeding, nest success, and notes of parental care in the Clay-colored Thrush (*Turdus grayi*): the role of females and males. *Wilson J. Ornithol.* **130**, 437–444. (doi:10.1676/17-002.1)

221. Vanderhoff N, Pyle P, Patten MA, Sallabanks R, James FC. 2020 American Robin (Turdus migratorius), version 1.0. *Birds World* (doi:10.2173/bow.amerob.01)

222. Weatherhead PJ, Mcrae SB. 1990 Brood care in American robins: implications for mixed reproductive strategies by females. *Anim. Behav.* **39**, 1179–1188.

223. Fitch Jr FW. 1950 Life history and ecology of the Scissor-tailed Flycatcher, Muscivora forficata. *The Auk* **67**, 145–168.

224. Regosin JV, Pruett-Jones S. 1995 Aspects of breeding biology and social organization in the Scissor-tailed Flycatcher. *The Condor* **97**, 154–164.

225. Woodard JD, Murphy MT. 1999 Sex roles, parental experience and reproductive success of eastern kingbirds,Tyrannus tyrannus. *Anim. Behav.* **57**, 105–115. (doi:10.1006/anbe.1998.0998)

226. Morehouse EL, Brewer R. 1968 Feeding of Nestling and Fledgling Eastern Kingbirds. *The Auk* **85**, 44–54. (doi:10.2307/4083623)

227. Chutter CM, Redmond LJ, Cooper NW, Dolan AC, Duffield D, Murphy MT. 2016 Paternal behaviour in a socially monogamous but sexually promiscuous passerine bird. *Behaviour* **153**, 443–466. (doi:10.1163/1568539X-00003359)

228. Pande S, Dahanukar N. 2012 Reversed sexual dimorphism and differential prey delivery in barn owls (Tyto alba). *J. Raptor Res.* **46**, 184–189.

229. Arlettaz R, Schaad M, Reichlin TS, Schaub M. 2010 Impact of weather and climate variation on Hoopoe reproductive ecology and population growth. *J. Ornithol.* **151**, 889–899. (doi:10.1007/s10336-010-0527-7)

230. Paredes R, Boness DJ, Jones IL. 2006 Parental roles of male and female thick-billed murres and razorbills at the Gannet Islands, Labrador. *Behaviour* **143**, 451–481.

231. In press. Golden-winged Warbler - Vermivora chrysoptera - Birds of the World. See https://birdsoftheworld.org/bow/species/gowwar/cur/introduction (accessed on 26 June 2022).

232. Ritchison G, Hawkins JA, Ritchison BC. 2019 Brooding and provisioning of nestlings by male and female White-eyed Vireos (Vireo griseus). *Avian Biol. Res.* **12**, 28–32. (doi:10.1177/1758155919832138)

233. Morton ES, Stutchbury BJM, Howlett JS, Piper WH. 1998 Genetic monogamy in blue-headed vireos and a comparison with a sympatric vireo with extrapair paternity. *Behav. Ecol.* **9**, 515–524. (doi:10.1093/beheco/9.5.515)

234. Caetano JV, Maia MR, Manica LT, Macedo RH. 2014 Immune-related effects from predation risk in Neotropical blue-black grassquits (Volatinia jacarina). *Behav. Processes* **109**, 58–63.

235. Niven DK. 1993 Male-male nesting behavior in Hooded Warblers. *Wilson Bull.* **105**, 190–193.

236. Miller AH, Miller VD. 1968 The behavioral ecology and breeding biology of the Andean sparrow, Zonotrichia capensis. *Caldasia* , 83–154.

237. Moore I, Class A. 2010 Is there a trade-off between caring for offspring and territorial aggression in tropical male rufous-collared sparrows (Zonotrichia capensis)? *Behaviour* **147**, 1819–1839. (doi:10.1163/000579510X538296)

238. Laubach ZM, Perng W, Lombardo M, Murdock C, Foufopoulos J. 2015 Determinants of parental care in Mountain White-crowned Sparrows (Zonotrichia leucophrys oriantha). *Auk Ornithol. Adv.* **132**, 893–902.

239. Morton ML, Orejuela JE, Budd SM. 1972 The biology of immature mountain white-crowned sparrows (Zonotrichia leucophrys oriantha) on the breeding ground. *The Condor* **74**, 423–430.

240. Kikkawa J, Wilson JM. 1983 Breeding and dominance among the Heron Island Silvereyes Zosterops lateralis chlorocephala. *Emu-Austral Ornithol.* **83**, 181–198.
