# Supplementary material for: The evolution of extra-pair paternity and paternal care in birds
Source: Behav Ecol. 2023 Jun 23;34(5):780–9. doi: 10.1093/beheco/arad053 (PMC10516673; doi:10.1093/beheco/arad053)
Supplement: arad053_suppl_Supplementary_Table_S19 [file arad053_suppl_supplementary_table_s19.pdf]

Supplementary materials

Table S19: Data for all species, with mean clutch size (Mean CS), body mass (BM) and maximum lifespan and their references. Sampling categories refer to sample size category from AnAge, categorized as 1) tiny: <10, 2) small: 10-100, 3) medium: 100-1000, 4) large: >1000. Comments section include all considerations that were made in relation to the given measurements.

Latin	English	Mean CS	Ref. CS	BM (g)	Ref. BM	Lifespan	Ref. lifespan	Sampling category	Comment
Acanthiza pusilla	Brown thornbill	3	[1]	7.475	[2]	17.6	[3]	4	
Accipiter cooperii	Cooper's hawk	4.5	[1]	439	[4]	20.3	[3]	3	
Acrocephalus arundinaceus	Great reed warbler	4.5	[5]	30	[2]	10.1	[3]	3	
Acrocephalus bistrigiceps	Black-browed reed-warbler	5	[1]	8.7	[2]	11.38	[3]		Mean max age for *Acrocephalus* used
Acrocephalus palustris	Marsh warbler	4	[5]	11.5	[2]	9.8	[3]	3	
Acrocephalus schoenobaenus	Sedge warbler	5.5	[5]	12.5	[2]	11.8	[3]	3	
Acrocephalus scirpaceus	Eurasian reed-warbler	4	[5]	12.3	[2]	16.9	[3]	4	
Actitis hypoleucos	Common sandpiper	4	[5]	48	[2]	14.5	[3]	3	
Aegolius funereus	Boreal owl	5	[5]	140.6	[2]	15.9	[3]	3	
Agelaius phoeniceus	Red-winged blackbird	4	[1]	52.4	[2]	20	[3]	3	
Agelaius xanthomus	Yellow-shouldered blackbird	2.5	[1]	38.4	[2]	12.6	[3]	3	
Alauda arvensis	Skylark	4	[5]	35	[2]	10.1	[3]	3	
Alle alle	Little auk	1	[5]	171	[2]	7.5	[6]		
Ammodramus maritimus	Seaside sparrow	4.5	[1]	22.225	[2]	9	[3]	3	
Ammodramus savannarum	Grasshopper Sparrow	4.5	[1]	17.56	[2]	9.1	[3]	3	
Anas platyrhynchos	Mallard	11	[5]	1126.25	[2]	29.1	[3]	3	
Anas strepera	Gadwalls	10	[5]	12.1	[2]	22.3	[3]	3	

Anthus spinoletta	Water pipit	5	[5]	23.9	[2]	8.8	[3]	3	
Aphrastura spinicauda	Thorn-tailed Rayadito	3.5	[1]	11.5	[2]	10.5	[3]	1	Mean max age for *Furnariidae* used
Apus apus	Common swift	2.5	[5]	37.6	[2]	21.1	[3]	3	
Aquila heliaca	Eastern imperial eagle	2.5	[5]	3262	[3]	25.9	[6]	3	
Ardea alba	Great egret	4	[5]	873.5	[2]	22.8	[3]	3	
Asio otus	Long-eared owl	4	[5]	229	[2]	27.8	[3]	2	
Athene cunicularia	Burrowing owl	8.5	[1]	151	[2]	11	[3]	3	
Athene noctua	Little owl	5	[5]	169	[2]	11.1	[3]	3	
Baeolophus bicolor	Tufted titmouse	6	[1]	21.6	[2]	13.3	[3]	3	
Bartramia longicauda	Upland sandpiper	4	[1]	159	[2]	8.9	[3]	3	
Branta bernicla	Black brant	4	[5]	1279.25	[2]	28.8	[3]	4	
Branta leucopsis	Barnacle goose	4.5	[5]	1687	[2]	28.2	[3]	3	
Buteo ridgwayi	Ridgway's hawk	2	[1]	356.55	[4]	23.075	[3]	4	Mean age for *Buteo* species used
Buteo swainsoni	Swainson’s hawk	2.5	[1]	958.5	[2]	24.9	[3]	3	
Calamospiza melanocorys	Lark bunting	3.5	[1]	37.6	[2]	4.8	[3]	3	
Calidris alba	Sanderling	4	[5]	57	[2]	18.6	[3]	2	
Calidris maritima	Purple sandpiper	4	[5]	62.7	[2]	20.8	[3]	3	
Calidris mauri	Western sandpiper	4	[5]	27.85	[2]	9.2	[3]	2	
Calonectris diomedea	Cory's shearwater	1	[5]	535	[2]	24.8	[3]	3	
Cardinalis cardinalis	Northern cardinal	3	[1]	42.65	[2]	28.5	[3]	3	
Carduelis cannabina	Eurasian linnet	5	[5]	19.55	[2]	9.4	[3]	3	
Carduelis tristis	American goldfinch	4.5	[1]	12.8	[2]	13	[3]	3	
Carpodacus erythrinus	Scarlet rosefinch	5	[1]	24	[2]	9	[3]	3	

Carpodacus mexicanus	House finch	5	[1]	21.4	[2]	11.6	[3]	3	
Catharacta lonnbergi	Brown skua	2	[1]	1687	[2]	28.8	[3]	3	
Catharacta maccormicki	South polar skua	2	[1]	1349	[2]	23.5	[3]	3	
Centropus phasianinus	Pheasant coucal	4	[1]	389.5	[2]	8.5	[3]	4	Mean age for *Cuculidae* used
Cepphus grylle	Black guillemot	1.5	[5]	378	[2]	29.9	[3]	2	
Cercomacra tyrannina	Dusky antbird	2	[1]	16.3	[2]	13	[3]	2	
Charadrius alexandrinus	Kentish plover	3	[5]	42.3	[2]	19	[3]	3	
Charadrius falklandicus	Two-banded plover	3	[1]	65	[2]	15.43	[3]	4	Mean max age for *Charadrius* used
Charadrius hiaticula	Ringed plover	3.5	[5]	64.05	[2]	20.8	[3]	3	
Charadrius marginatus	White-fronted plover	2	[1]	48.3	[2]	15.43	[3]	4	Mean age for *Charadrius* used
Charadrius modestus	Rufous-chested dotterel	2	[1]	78	[2]	15.43	[3]	4	Mean age for *Charadrius* used
Charadrius nivosus	Snowy plover	4	[1]	42.3	[2]	19	[3]	3	
Charadrius pecuarius	Kittlitz's plover	2	[5]	34	[2]	15.43	[3]	4	Mean age for *Charadrius* used
Charadrius ruficapillus	Red-capped plover	2	[1]	37.4	[2]	21	[3]	3	
Charadrius semipalmatus	Semipalmated plover	3.5	[5]	46.75	[2]	9.2	[3]	2	
Charadrius thoracicus	Madagascar plover	1.5	[1]	11.5	[2]	15.43	[3]	4	Mean max age for *Charadrius* used
Chen caerulescens	Lesser snow goose								
Chen rossii	Ross's goose	4	[1]	1636	[2]	22.5	[3]	4	
Chlidonias hybrida	Whiskered tern	2.5	[5]	83.9	[2]	16	[3]	2	
Chlidonias niger	North American black tern	2.5	[1]	65.3	[2]	21	[3]	1	
Cinclus cinclus	White-throated dipper	4.5	[5]	61.7	[2]	10.6	[3]	4	
Circus pygargus	Montagu's harrier	4.5	[5]	315.5	[2]	16.3	[3]	3	
Cistothorus platensis	Grass wrens	5	[1]	9.583	[2]	9.25	[3]	3	Mean max age for *Troglodytidae* used
Coracias garrulus	European roller	4.5	[5]	146	[2]	9.2	[3]	3	

Coragyps atratus	Black vulture	2	[1]	1899.5	[2]	25.5	[3]	3	
Corvus corone	Crow	4	[1]	570	[2]	19.2	[3]	3	
Corvus monedula	Eurasian jackdaw	5	[5]	246	[2]	20.3	[3]	3	
Cyanocitta stelleri	Steller's jay	4	[1]	128	[2]	16.1	[3]	3	
Cyanoliseus patagonus	Burrowing parakeet	3.5	[1]	278	[2]	20.6	[3]	4	Max age for *Trichoglossus haematodus* used
Cygnus atratus	Black swan	5	[1]	5650	[2]	24.7	[3]	4	
Delichon urbicum	House martin	4	[5]	14.5	[2]	15	[3]	3	
Dendrocopos major	Great spotted woodpecker	5.5	[5]	81.6	[2]	12.7	[3]	3	
Dendrocopos medius	Middle spotted woodpecker	5.5	[5]	59	[2]	9.3	[3]	3	
Dendroica caerulescens	Black-throated blue warbler	5.5	[1]	10.15	[2]	10	[3]	2	
Dendroica pensylvanica	Chestnut-sided warbler	4	[1]	9.3	[2]	6.9	[3]	2	
Dendroica petechia	Yellow warbler	4	[1]	10.16	[2]	11	[3]	3	
Diomedea exulans	Wandering albatross	1	[1]	7245	[2]	50	[3]	3	
Dolichonyx oryzivorus	Bobolinks	5	[1]	31.55	[2]	9	[3]	3	
Dumetella carolinensis	Grey catbird	3.5	[1]	35.3	[2]	17.9	[3]	4	
Elaenia chiriquensis	Lesser elaenia	2	[1]	15.4	[2]	13.6	[3]	3	Max age for *Elaenia martinica* used
Elaenia flavogaster	Yellow-bellied elaenia	2	[1]	24.8	[2]	13.6	[3]	3	Max age for *Elaenia martinica* used
Emberiza citrinella	Yellowhammer	4.5	[5]	29.7	[2]	13.2	[3]	3	
Emberiza schoeniclus	Reed bunting	4.5	[5]	18.45	[2]	12.2	[3]	3	
Emberiza schoeniclus	Reed bunting	4.5	[5]	18.45	[2]	12.2	[3]	3	
Empidonax minimus	Least flycatcher	4	[1]	10	[2]	8	[3]	3	
Empidonax traillii	Willow flycatcher	3.5	[1]	13.4	[2]	11	[3]	3	
Empidonax virescens	Acadian flycatcher	3	[1]	12.6	[2]	12.1	[3]	3	

Erithacus rubecula	European Robin	5.5	[5]	17.7	[2]	19.3	[3]	4	
Erythrura gouldiae	Gouldian finch	6	[1]	13.8	[2]	2.2	[3]	2	Max age for *Erythrura trichroa* used
Eudromias morinellus	Eurasian dotterel	3	[5]	113.5	[2]	11.8	[3]	3	
Eudyptes pachyrhynchus	Fiordland penguin	2	[1]	3910	[2]	27.4	[3]		
Eudyptes schlegeli	Royal penguin	2	[1]	4250	[2]	15.8	[3]	1	
Euplectes orix	Red bishop	3	[1]	23.1	[2]	12	[3]		
Falco columbarius	Merlin	4	[5]	190.5	[2]	12.7	[3]	3	
Falco eleonorae	Eleonoras falcon	2.5	[5]	390	[2]	11.2	[3]	3	
Falco naumanni	Lesser kestrel	4	[5]	152.5	[2]	10.9	[3]	3	
Falco peregrinus	Peregrine falcon	3.5	[5]	697.5	[2]	25	[3]	3	
Falco sparverius	American kestrel	5	[1]	116.21	[2]	9.8	[3]	3	
Falco tinnunculus	Eurasian kestrel	4.5	[5]	184	[2]	22.8	[3]	4	
Ficedula albicollis	Collared flycatcher	6	[5]	12.7	[2]	9.8	[3]	3	
Ficedula hypoleuca	Pied flycatcher	6.5	[5]	13.9	[2]	15	[3]	4	
Ficedula parva	Red-breasted flycatcher	5.5	[5]	9.9	[2]	12.4	[3]	4	Mean max age of *Ficedula albicollis* and *Ficedula hypoleuca* used
Ficedula zanthopygia	Yellow rumped flycatcher	6	[1]	11.2	[2]	12.4	[3]	4	Mean max age of *Ficedula albicollis* and *Ficedula hypoleuca* used
Fratercula arctica	Atlantic puffin	1	[5]	408.8	[2]	45	[3]	4	
Fregata minor	Great frigatebird	1	[1]	1296	[2]	43	[3]	3	
Fringilla coelebs	Chaffinch	4.5	[5]	21.4	[2]	15.5	[6]	4	
Fulmarus glacialis	Northern fulmar	1	[5]	613	[2]	51	[3]	3	
Furnarius rufus	Rufous Hornero	3	[1]	47	[2]	10.5	[3]	2	
Gallinula chloropus	Moorhen	7	[5]	343.5	[2]	18.6	[3]	3	Mean max age for *Furnariidae* used

Gavia immer	Common loon	2	[5]	4980	[2]	24.1	[3]	3	
Geospiza fortis	Medium ground-finch	4	[1]	24	[2]	15	[3]	2	Max age for *Geospiza fuliginosa* used
Geospiza scandens	Cactus finch	3.5	[1]	22.7	[2]	15	[3]	2	Max age for *Geospiza fuliginosa* used
Geothlypis trichas	Yellowthroat	4	[1]	9.58	[2]	11.5	[3]	3	
Gerygone flavolateralis	Fan-tailed Gerygone	2.5	[1]	6.3	[2]	6.6	[3]	3	Mean max age for *Gerygone* species used
Grallina cyanoleuca	Australian magpie-lark	3.5	[1]	82.775	[2]	12.1	[3]	3	
Grus canadensis	Sandhill crane	2	[1]	4390.5	[2]	36.6	[3]	3	
Gyps fulvus	Griffon vulture	1	[5]	7436	[2]	25.15	[3]	3	Mean max age for *Gyps africanus* and Gyps coprotheres used
Habia fuscicauda	Red-throated ant-tanager	3	[1]	35.4	[2]	13.18	[3]	3	Mean max age for *Cardinalidae* used
Haematopus ostralegus	Eurasian oystercatcher	3	[5]	526	[2]	43.3	[3]	3	
Haliaeetus albicilla	White-tailed eagle	2	[5]	4793	[2]	25	[3]	3	
Hirundo ariel	Fairy martin	3.5	[1]	10.85	[2]	5.3	[3]	4	
Hirundo rustica	Barn swallow	4.5	[5]	18.3	[2]	16	[3]	3	
Hylocichla mustelina	Wood thrush	3.5	[1]	100.3	[2]	10.2	[3]	3	
Hymenolaimus malacorhynchos	Blue duck	5.5	[1]	832.5	[2]	24.11	[3]	4	Mean max age for *Anatidae* used
Icteria virens	Yellow-breasted chat	4	[1]	26	[3]	8.9	[3]	2	
Icterus galbula	Bullock´s oriole	5	[1]	32.85	[2]	14	[3]	3	
Jabiru mycteria	Jabiru	3.5	[1]	6404.5	[2]	39	[3]	3	Max age for *Ciconia ciconia* used
Junco hyemalis	Dark-eyed junco	4	[1]	19.89	[2]	11.3	[3]	4	
Jynx torquilla	Eurasian wryneck	8.5	[5]	35	[2]	10	[3]	3	
Lagopus lagopus	Willow ptarmigan	8.5	[5]	570.6	[2]	9	[3]	2	
Lagopus leucura	White-tailed ptarmigan	6.5	[1]	355	[2]	15	[3]	2	

Laniarius atrococcineus	Crimson-breasted shrike	2.5	[1]	48.8	[2]	8.25	[7]		
Lanius bucephalus	Bull-headed shrike	5.5	[1]	39.75	[2]	8.1	[3]		
Lanius ludovicianus	Loggerhead shrike	6	[1]	51.75	[2]	12.5	[3]	2	
Lanius minor	Lesser gray shrike	5.5	[5]	46.5	[2]	10.075	[3]	3	Mean max age for *Lanius* used
Larus canus	Common gull	3	[5]	403.5	[2]	33.7	[3]	3	
Larus occidentalis	Western gull	3	[1]	931.5	[2]	33.9	[3]	3	
Larus ridibundus	Black-headed gull	2.5	[5]	284	[2]	32.9	[3]	3	
Lichenostomus chrysops	Yellow-faced honeyeater	2.5	[1]	17.3	[2]	12.6	[3]	4	
Locustella luscinioides	Savi's warbler	4	[5]	13.9	[2]	9.8	[3]	3	
Loxia curvirostra	Red crossbill	3.5	[5]	38.5	[2]	16.1	[3]	2	
Loxioides bailleui	Palila	2	[1]	37.85	[2]	13	[3]	3	
Luscinia megarhynchos	Common nightingale	4.5	[5]	19.63	[2]	10.9	[3]	3	
Luscinia svecica	Bluethroat	5.5	[5]	17.78	[2]	11.4	[3]	3	
Megascops asio	Eastern screech-owl	3.5	[1]	180.5	[2]	12.9	[3]	3	Max age for *Megascops kennicottii* used
Melospiza georgiana	Swamp sparrow	3.5	[1]	16.1	[2]	7.8	[3]	3	
Melospiza melodia	Song sparrow	3.5	[1]	23.17	[2]	11.3	[3]	3	
Miliaria calandra	Corn bunting	5	[5]	48.8	[3]	10.6	[3]	3	
Mycteria americana	Wood stork	3	[5]	2558.5	[2]	39	[3]	2	Max age for *Ciconia ciconia* used
Myiopsitta monachus	Monk parakeet	6	[1]	120	[2]	20.6	[3]	4	Max age for *Trichoglossus haematodus* used
Nectarinia osea	Orange-tufted sunbird	2	[1]	7.2	[2]				
Notiomystis cincta	New Zealand hihi	4	[1]	93.5	[2]				
Oceanites oceanicus	Wilson's storm-petrel	1	[1]	30.45	[2]	19.8	[3]	2	Mean max age for *Oceanitidae* used
Oceanodroma leucorhoa	Leach's storm-petrel	1	[5]	37.05	[2]	36	[3]	3	

Oenanthe oenanthe	Northern wheatear	5.5	[5]	22.65	[2]	10.1	[3]	3	
Otus elegans	Elegant scops-owl	2.5	[1]	124	[2]	6.9	[3]	3	
Otus flammeolus	Flammulated owl	3	[1]	60.15	[2]	8.1	[3]	3	Max age for *Otus scops* used
Pachycephala pectoralis	Golden whistlers	2.5	[1]	30.31	[2]	18	[3]	4	
Pachyptila belcheri	Thin-billed prion	1	[1]	145	[2]	16.5	[3]	2	Max age for *Pachyptila turtur* used
Panurus biarmicus	Bearded tit	6	[5]	13.2	[2]	7.1	[3]	3	
Paradoxornis webbianus	Vinous-throated parrotbills	5	[1]	10.9	[2]				
Parus ater	Coal tit	9	[5]	9.2	[2]	9.5	[3]	3	
Parus atricapillus	Black-capped chickadee	7	[1]	10.8	[2]	12.4	[3]	4	
Parus caeruleus	Blue tit	10	[5]	10.35	[2]	14.6	[3]	3	
Parus cristatus	Crested tit	8	[5]	11.5	[2]	11.6	[3]	3	
Parus major	Great tit	10.5	[5]	18.25	[2]	15.4	[3]	4	
Parus montanus	Willow tit	7.5	[5]	11.1	[2]	11.3	[3]	3	
Parus teneriffae	African blue tit								
Parus varius	Varied tit	6.5	[1]	17	[2]	10.84	[3]	4	Mean max age for *Paridae* used
Passer domesticus	House sparrow	4	[5]	27.7	[2]	23	[3]	3	
Passer montanus	Tree sparrow	4.5	[5]	22	[2]	13.1	[3]	3	
Passerculus sandwichensis	Savannah sparrow	4.5	[1]	20.61	[2]	6.8	[3]	3	
Passerina caerulea	Blue grosbeak	4	[1]	27.4	[2]	10.2	[3]	1	
Passerina cyanea	Indigo bunting	3.5	[1]	14.7	[2]	11	[3]	3	
Perisoreus canadensis	Canada jay	3.5	[1]	70.3	[3]	19.2	[3]	2	
Petroica australis	New Zealand robin	2.5	[1]	31.65	[2]	7.5	[3]	3	Mean max age for *Petroica species* used
Petroica goodenovii	Red-capped robin	2.5	[1]	8.9	[2]	5.5	[3]	3	
Petronia petronia	Rock sparrow	5.5	[5]	30.2	[2]	7	[3]	3	

Phainopepla nitens	Phainopepla	2.5	[1]	22.1	[2]		[3]		
Phalacrocorax aristotelis	Shag	3.5	[5]	1773	[2]	30.6	[3]	3	
Phalacrocorax atriceps	Imperial shag								
Phalacrocorax carbo	Great cormorant	3.5	[5]	2571.16	[2]	32.1	[3]	4	
Phalaropus lobatus	Red-necked phalarope	4	[5]	36.75	[2]	12.9	[3]	1	
Philesturnus carunculatus	Saddleback	1.5	[1]	74.95	[2]		[3]		
Phoebastria irrorata	Waved albatross	1	[1]	3395	[2]	40	[3]	3	
Phoenicurus ochruros	Black redstart	5	[5]	16.5	[2]	10.2	[3]	3	
Phoenicurus phoenicurus	Common redstart	6	[5]	14.6	[2]	10.2	[3]	3	
Phylidonyris pyrrhopterus	Crescent honeyeater	2	[1]	16.4	[2]	9.9	[3]	4	
Phylloscopus fuscatus	Dusky warbler	5	[1]	8.75	[2]	10	[3]	3	Mean max age for *Phylloscopus* used
Phylloscopus sibilatrix	Wood warbler	6	*[5]*	9.2	[2]	10.2	[3]	3	
Phylloscopus trochilus	Willow warbler	6	[5]	8.7	[2]	11.8	[3]	3	
Picoides tridactylus	Eurasian three-toed woodpecker	4	[5]	65.7	[2]	11.2	[3]	3	
Pipilo maculatus	Spotted towhee	3.5	[1]	39.225	[2]	11	[3]	3	
Piranga olivacea	Scarlet tanager	3.5	[1]	28.2	[2]	11.9	[3]	3	
Platalea ajaja	Roseate spoonbill	3	[1]	1490	[2]	28	[3]	3	
Platycercus elegans	Crimson Rosella	5	[1]	132.5	[2]		[3]		
Plectrophenax nivalis	Snow bunting	5	*[5]*	42.2	[2]	10.2	[3]	4	
Pluvialis dominica	American golden plover	4	[1]	151.5	[2]	13	[3]	2	
Poecile gambeli	Mountain chickadees	6	[1]	11.1	[2]	10.1	[3]	2	
Poecile palustris	Marsh Tits	8	[5]	11.15	[2]	13.2	[3]	3	
Poephila acuticauda	Long-tailed finch	4.5	[1]	14	[2]	6	[3]	3	

Porphyrio hochstetteri	Takahe	2	[1]	2470.5	[2]	8.4	[3]	2	Max age for *Porphyrio porphyrio* used
Prinia flaviventris	Yellow-bellied prinia	4	[1]	7	[2]	3.6	[3]	2	Max age for *Cisticola exilis* used
Progne subis	Purple martin	4.5	[1]	53.8	[2]	13.8	[3]	2	
Promerops cafer	Cape sugarbird	2	[1]	34.85	[2]		[3]		
Prosthemadera novaeseelandiae	Tui	3	[1]	107.3	[2]	12.57	[3]	4	Mean max age for *Meliphagidae* used
Puffinus tenuirostris	Short-tailed shearwater	1	[1]	559	[2]	39.9	[3]	3	
Pygoscelis adeliae	Adélie penguin	2	[1]	4850	[2]	26.66	[3]	4	Mean max age for *Spheniscidae* used
Pygoscelis antarcticus	Chinstrap penguin	2	[1]	4155.5	[2]	26.66	[3]	4	Mean max age for *Spheniscidae* used
Pygoscelis papua	Gentoo penguin	2	[1]	5950	[2]	26.66	[3]	4	Mean max age for *Spheniscidae* used
Quelea quelea	Red-billed quelea	3	[1]	19.4	[2]	10	[3]	4	
Ramphocelus costaricensis	Cherrie's tanager	2	[1]	32	[2]	9.75	[3]	3	Mean max age for *Eucometis penicillata* and *Thraupis episcopus* used
Remiz coronatus	White-crowned penduline tit	6	[1]	6.9	[2]	7.2	[3]	4	Max age for *Remiz pendulinus* used
Rhipidura fuliginosa	Grey fantail	3	[1]	7.55	[2]	9.25	[3]	3	Mean max age for *Rhipidura rufifrons* and *Rhipidura leucophrys* used
Riparia riparia	Sand martin	5	[5]	12.375	[2]	10.1	[3]	4	
Rissa tridactyla	Black-legged kittiwake	2	[5]	420.75	[2]	28.5	[3]	3	
Sayornis phoebe	Eastern phoebe	5	[1]	19.7	[2]	10.3	[3]	3	
Serinus canaria	Canary	3.5	[5]	24.3	[2]	13.3	[3]	3	Body mass and max age for *Serinus serinus* used
Serinus serinus	Serin	3.5	[5]	11.2	[2]	13.3	[3]	3	
Setophaga ruticilla	American redstart	3.5	[1]	8.25	[2]	10.1	[3]	3	
Sialia currucoides	Mountain bluebird	6	[1]	29.6	[2]	9	[3]	3	

Sialia sialis	Eastern bluebird	5	[1]	27.5	[2]	10.5	[3]	3	
Sicalis flaveola	Saffron finch	3	[1]	17	[2]	11.6	[3]	3	Mean max age for *Thraupidae* used
Sitta europaea	European nuthatch	7	[5]	20.8	[2]	12.9	[3]	3	
Spheniscus humboldti	Humboldt penguin	2	[1]	4379	[2]	26.66	[3]	4	Mean max age for *Spheniscidae* used
Spheniscus magellanicus	Magellanic Penguin	2	[1]	4120	[2]	30	[3]	3	
Spiza americana	Dickcissel	4.5	[1]	26.275	[2]	4.8	[3]	3	
Spizella pusilla	field sparrow	4	[1]	12.5	[2]	10.3	[3]	3	
Steganopus tricolor	Wilson's phalarope	4	[1]	59.95	[2]	10	[3]	3	
Sterna hirundo	Common tern	2	[5]	128.5	[2]	33	[3]	3	
Strix aluco	Tawny owl	3.5	[5]	475	[2]	22.4	[3]	3	
Sturnus unicolor	Spotless starling	4.5	[5]	86	[2]	22.9	[3]	4	Body mass and max age for *Sturnus vulgaris* used
Sturnus vulgaris	Common starling	5	[5]	86	[2]	22.9	[3]	4	
Sula dactylatra	Masked booby	1.5	[1]	1734.16	[2]	25.5	[3]	4	
Sula granti	Nazca booby	1.5	[1]	1754	[2]	21	[3]	3	
Sula nebouxii	Blue-footed booby	2	[1]	1542	[2]	18	[3]	3	
Sula sula	Red-footed booby	1	[1]	1048.25	[2]	23	[3]	3	
Tachycineta albilinea	Mangrove swallow	4	[1]	16.7	[2]	10.6	[3]	3	Mean max age for *Tachycineta bicolor* and *Tachycineta thalassina* used
Tachycineta bicolor	Tree swallow	5.5	[1]	21.2	[2]	12.1	[3]	3	
Tachycineta leucorrhoa	White rumped swallow	5.5	[1]	15.3	[2]	10.6	[3]	3	Mean max age for *Tachycineta bicolor* and *Tachycineta thalassina* used
Tachycineta meyeni	Chilean swallow	5	[1]	14.9	[2]	10.6	[3]	3	Mean max age for *Tachycineta bicolor* and *Tachycineta thalassina* used

Taeniopygia guttata	Zebra finch	5	[1]	12.05	[2]	12	[3]	4	
Thalassarche cauta	Shy albatross	1	[1]	3911.25	[2]	30.9	[3]	3	
Thalassarche chrysostoma	Grey-headed albatross	1	[1]	3507.5	[2]	47.2	[3]	3	
Thalassarche melanophrys	Black-browed albatross	1	[1]	3519.56	[2]	43.7	[3]	3	
Thalassoica antarctica	Antarctic petrel	1	[1]	708.5	[2]	15	[3]	3	
Thamnophilus atrinucha	Black-crowned antshrike	2	[1]	23.6	[2]	14.9	[3]	2	Mean max age for *Thamnophilidae* used
Thryothorus ludovicianus	Carolina wren	4.5	[1]	18.575	[2]	9.2	[3]	3	
Thryothorus pleurostictus	Banded wren	3.5	[1]	17.7	[2]	9.2	[3]	3	Max age for *Thryothorus ludovicianus* used
Thryothorus rufalbus	Rufous-and-white wren	2.5	[1]	23.61	[2]	9.2	[3]	3	Max age for *Thryothorus ludovicianus* used
Tockus monteiri	Monteiro’s hornbill	5	[1]						
Troglodytes aedon	House wren	6	[1]	10.83	[2]	9	[3]	3	
Troglodytes troglodytes	Eurasian wren	6.5	[5]	9.91	[2]	7	[3]	3	
Turdus albicollis	White-necked thrush	2.5	[1]						
Turdus grayi	Clay-colored robin	3	[1]	79.5	[2]	9.6	[3]	3	
Turdus migratorius	American robin	3.5	[1]	78.5	[2]	17	[3]	3	
Tyrannus forficatus	Scissor-tailed flycatchers	4.5	[1]	39.3	[2]	9	[3]	2	Mean max age for *Tyrannus verticalis* and *Tyrannus tyrannus* used
Tyrannus tyrannus	Eastern kingbird	3.5	[1]	39.925	[2]	11.1	[3]	2	
Tyto alba	Barn owl	5.5	[5]	350	[2]	34	[3]	3	
Upupa epops	Eurasian hoopoe	7.5	[5]	65.7	[2]	11.1	[3]	3	
Uria lomvia	Thick-billed murres	1	[5]	964	[2]	29	[3]	3	

Uria aalge	Common murres	1	[5]	991.75	[2]	42.8	[3]	4	
Vermivora chrysoptera	Golden-winged warbler	5	[1]	8.75	[2]	7.9	[3]	1	
Vireo griseus	White-eyed vireo	4	[1]	11.4	[2]	10.9	[3]	2	
Vireo olivaceus	Red-eyed vireo	3	[1]	16.16	[2]	10.2	[3]	2	
Vireo solitarius	Blue-headed vireo	3.5	[1]	15.3	[2]	7.4	[3]	3	
Volatinia jacarina	Blue-black grassquits	3	[1]	9.95	[2]	11.6	[3]	3	Mean max age for *Thraupidae* used
Wilsonia citrina	Hooded warbler	4	[1]	10.55	[2]	8.2	[3]	3	
Zonotrichia albicollis	White-throated sparrow	4	[1]	24.4	[2]	14.9	[3]	3	
Zonotrichia capensis	Rufous-collared sparrow	2.5	[1]	20.19	[2]	12.6	[3]	4	Mean max age for *Zonotrichia* species used
Zonotrichia leucophrys	White-crowned sparrow	5	[1]	27.45	[2]	13.3	[3]	4	
Zosterops lateralis	Capricorn silvereye	3	[1]	12.7	[2]	18.7	[3]	4	

References

1. Del Hoyo J, Del Hoyo J, Elliott A, Sargatal J. 1992 *Handbook of the birds of the world*. Lynx edicions Barcelona.

2. Dunning Jr JB. 2007 *CRC handbook of avian body masses*. CRC press.

3. Tacutu R *et al.* 2018 Human ageing genomic resources: new and updated databases. *Nucleic Acids Res.* **46**, D1083–D1090.

4. Woolaver LG, Nichols RK, Morton E, Stutchbury BJ. 2013 Nestling sex ratio in a critically endangered dimorphic raptor, Ridgway's Hawk (Buteo ridgwayi). *J. Raptor Res.* **47**, 117–126.

5. Cramp S. 1977 *The Birds of the Western Palearctic*. Oxford: Oxford Univ. Press.

6. Fransson T. 2010 EURING list of longevity records for European birds. *Httpwww Euring Orgdataandcodeslongevity-Voous Htm*

7. Fry H. 2020 Crimson-breasted Gonolek (Laniarius atrococcineus), version 1.0. *Birds World* (doi:10.2173/bow.crbgon1.01)
