# Supplementary material for: The evolution of extra-pair paternity and paternal care in birds
Source: Behav Ecol. 2023 Jun 23;34(5):780–9. doi: 10.1093/beheco/arad053 (PMC10516673; doi:10.1093/beheco/arad053)
Supplement: arad053_suppl_Supplementary_Table_S20 [file arad053_suppl_supplementary_table_s20.pdf]

Supplementary materials

Tabell S20: Data and references on proportion of male nestbuilding and incubation for the included species. Columns marked with * represent values when only studies providing values are included, and not interpreted results.

Latin	**English name**	**% NB**	**Ref**	**% NB***	**Ref**	**% Inc**	**Ref**	**% Inc***	**Ref**
Acanthiza pusilla	Brown thornbill	0	[1]	0	[1]	0	[1]	0	[1]
Accipiter cooperii	Cooper's hawk	0.69	[2]	0.69	[2]				
Acrocephalus arundinaceus	Great reed warbler	0	[3]	0	[3]	0	[3]	0	[3]
Acrocephalus bistrigiceps	Black-browed reed-warbler	0	[4]	0	[4]	0	[4]	0	[4]
Acrocephalus palustris	Marsh warbler	0	[3]	0	[3]	0.506	[5]	0.506	[5]
Acrocephalus schoenobaenus	Sedge warbler	0	[3]	0	[3]	0	[3]	0	[3]
Acrocephalus scirpaceus	Eurasian reed-warbler	0	[3]	0	[3]	0.35	[6–8]	0.35	[6–8]
Actitis hypoleucos	Common sandpiper					0.5	[9]		
Aegolius funereus	Boreal owl					0	[3]	0	
Agelaius phoeniceus	Red-winged blackbird	0	[1]	0		0	[1]	0	
Agelaius xanthomus	Yellow-shouldered blackbird	0	[1]	0		0	[1]	0	
Alauda arvensis	Skylark	0	[3]	0		0	[3]	0	
Alle alle	Little auk					0.5	[10,11]		
Ammodramus maritimus	Seaside sparrow	0	[1]	0	[1]	0	[1]	0	[1]
Ammodramus savannarum	Grasshopper Sparrow	0	[12]	0	[12]	0	[1]	0	[1]
Anas platyrhynchos	Mallard	0	[3]	0	[3]	0	[3]	0	[3]
Anas strepera	Gadwalls	0	[3]	0	[3]	0	[3]	0	[3]
Anthus spinoletta	Water pipit	0	[3]	0	[3]	0	[3]	0	[3]
Aquila heliaca	Eastern imperial eagle	0.1157	[13]	0.1157	[13]	0.059	[13]	0.059	[13]
Asio otus	Long-eared owl					0	[3]	0	[3]
Athene cunicularia	Burrowing owl					0	[1]	0	[1]
Athene noctua	Little owl					0	[3]	0	[3]

Baeolophus bicolor	Tufted titmouse	0	[1]	0	[1]	0	[1]	0	[1]
Branta bernicla	Black brant					0	[3]	0	[3]
Branta leucopsis	Barnacle goose					0	[3]	0	[3]
Buteo ridgwayi	Ridgway's hawk	0.76	[14]	0.76	[14]				
Buteo swainsoni	Swainson’s hawk					0	[15]	0	[15]
Calamospiza melanocorys	Lark bunting	0	[1]	0	[1]	0	[1]	0	[1]
Calidris alba	Sanderling	0	[3]	0	[3]				
Calidris maritima	Purple sandpiper	1	[16]	1	[16]	0.481	[17]	0.481	[17]
Calonectris diomedea	Cory's shearwater					0.5294	[18]	0.5294	[18]
Cardinalis cardinalis	Northern cardinal	0	[1]	0	[1]	0	[1]	0	[1]
Carduelis cannabina	Eurasian linnet	0	[3]	0	[3]	0	[3]	0	[3]
Carduelis tristis	American goldfinch	0	[1]	0	[1]	0	[1]	0	[1]
Carpodacus erythrinus	Scarlet rosefinch	0	[1]	0	[1]	0	[1]	0	[1]
Carpodacus mexicanus	House finch	0	[1]	0	[1]	0	[1]	0	[1]
Catharacta maccormicki	South polar skua					0	[19]	0	[19]
Centropus phasianinus	Pheasant coucal	0.9	[20]	0.9	[20]	1	[20]	1	[20]
Cercomacra tyrannina	Dusky antbird					0.5795	[21]	0.5795	[21]
Charadrius alexandrinus	Kentish plover					0.411	[22]	0.411	[22]
Charadrius falklandicus	Two-banded plover					0.3695	[23]	0.3695	[23]
Charadrius hiaticula	Ringed plover					0.515	[24]	0.515	[24]
Charadrius modestus	Rufous-chested dotterel					0.375	[25]	0.375	[25]
Charadrius ruficapillus	Red-capped plover					0	[1]	0	[1]
Charadrius semipalmatus	Semipalmated plover					0.5	[26]		
Chen rossii	Ross's goose					0	[27]	0	[27]
Chlidonias hybrida	Whiskered tern					0.5	[28]		
Cinclus cinclus	White-throated dipper					0	[3]	0	[3]

Circus pygargus	Montagu's harrier	0	[3]	0	[3]	0	[3]	0	[3]
Cistothorus platensis	Grass wrens	1	[29]	1	[29]	0	[30]	0	[30]
Coragyps atratus	Black vulture					0.5	[31]		
Corvus corone	Carrion crow					0	[1]	0	[1]
Corvus monedula	Eurasian jackdaw					0	[3]	0	[3]
Cyanocitta stelleri	Steller’s jay					0	[1]	0	[1]
Cyanoliseus patagonus	Burrowing parakeet					0	[32]	0	[32]
Cygnus atratus	Black swan					0.576	[33]	0.576	[33]
Delichon urbicum	House martin					0.516	[34]	0.516	[34]
Dendrocopos medius	Middle spotted woodpecker	0.705	[35]	0.705	[35]				
Dendroica caerulescens	Black-throated blue warbler	0	[36]	0	[36]	0	[36]	0	[36]
Dendroica pensylvanica	Chestnut-sided warbler	0	[37]	0	[37]	0	[37]	0	[37]
Dendroica petechia	Yellow warbler	0	[38]	0	[38]	0	[38]	0	[38]
Diomedea exulans	Wandering albatross					0.5604	[39]	0.5604	[39]
Dolichonyx oryzivorus	Bobolinks	0	[1]	0	[1]	0	[1]	0	[1]
Dumetella carolinensis	Grey catbird	0	[1]	0	[1]	0	[1]	0	[1]
Elaenia chiriquensis	Lesser elaenia	0	[40]	0	[40]	0	[40]	0	[40]
Elaenia flavogaster	Yellow-bellied elaenia					0	[40]	0	[40]
Emberiza citrinella	Yellowhammer	0	[3]	0	[3]	0	[3]	0	[3]
Emberiza schoeniclus	Reed bunting	0	[3]	0	[3]	0	[3]	0	[3]
Empidonax minimus	Least flycatcher	0	[41]	0	[41]	0	[41]	0	[41]
Empidonax traillii	Willow flycatcher	0	[42]	0	[42]	0	[42]	0	[42]
Empidonax virescens	Acadian flycatcher	0	[43]	0	[43]	0	[43]	0	[43]
Erithacus rubecula	European Robin	0	[3]	0	[3]	0	[3]	0	[3]
Eudromias morinellus	Eurasian dotterel	1	[3]	1	[3]	1	[3]	1	[3]
Euplectes orix	Red bishop	1	[44]	1	[44]	0	[1]	0	[1]

Falco columbarius	Merlin					0.072	[45]	0.072	[45]
Falco eleonorae	Eleonoras falcon					0	[3]	0	[3]
Falco naumanni	Lesser kestrel					0	[3]	0	[3]
Falco peregrinus	Peregrine falcon					0.2155	[46]	0.2155	[46]
Falco tinnunculus	Eurasian kestrel					0	[3]	0	[3]
Ficedula albicollis	Collared flycatcher	0	[3]	0	[3]	0	[3]	0	[3]
Ficedula hypoleuca	Pied flycatcher	0	[3]	0	[3]	0	[3]	0	[3]
Ficedula parva	Red-breasted flycatcher	0	[3]	0	[3]	0	[3]	0	[3]
Ficedula zanthopygia	Yellow rumped flycatcher	0	[47]	0	[47]	0	[48]	0	[48]
Fringilla coelebs	Chaffinch	0	[3]	0	[3]	0	[3]	0	[3]
Fulmarus glacialis	Northern fulmar					0.555	[49,50]	0.555	[49,50]
Gavia immer	Common loon					0.5154	[51,52]	0.5154	[51,52]
Geospiza scandens	Cactus finch					0	[1]	0	[1]
Geothlypis trichas	Yellowthroat	0	[53]	0	[53]	0	[53]	0	[53]
Grus canadensis	Sandhill crane					0.66	[54]	0.66	[54]
Habia fuscicauda	Red-throated ant-tanager	0	[1]	0	[1]	0	[1]	0	[1]
Haematopus ostralegus	Eurasian oystercatcher					0.5	[55]		
Hirundo ariel	Fairy martin					0.448	[56]	0.448	[56]
Hirundo rustica	Barn swallow					0	[3]	0	[3]
Hylocichla mustelina	Wood thrush	0	[57]	0	[57]	0	[57]	0	[57]
Hymenolaimus malacorhynchos	Blue duck					0	[58]	0	[58]
Icteria virens	Yellow-breasted chat	0	[59]	0	[59]	0	[59]	0	[59]
Icterus galbula	Bullock´s oriole	0	[60]	0	[60]	0	[60]	0	[60]
Junco hyemalis	Dark-eyed junco	0	[1]	0	[1]	0	[1]	0	[1]
Lagopus lagopus	Willow ptarmigan					0	[1]	0	[1]
Lagopus leucura	White-tailed ptarmigan					0	[61]	0	[61]

Lanius bucephalus	Bull-headed shrike					0	[62]	0	[62]
Lanius ludovicianus	Loggerhead shrike	0	[1]	0	[1]	0	[1]	0	[1]
Lanius minor	Lesser gray shrike					0	[3]	0	[3]
Larus occidentalis	Western gull					0.4455	[63]	0.4455	[63]
Lichenostomus chrysops	Yellow-faced honeyeater	0	[1]	0	[1]	0	[1]	0	[1]
Loxia curvirostra	Red crossbill	0	[3]	0	[3]	0	[3]	0	[3]
Loxioides bailleui	Palila					0	[1]	0	[1]
Luscinia svecica	Bluethroat	0	[64]	0	[64]	0	[64]	0	[64]
Megascops asio	Eastern screech-owl					0	[1]	0	[1]
Melospiza georgiana	Swamp sparrow	0	[1]	0	[1]	0	[1]	0	[1]
Melospiza melodia	Song sparrow	0	[1]	0	[1]	0	[1]	0	[1]
Miliaria calandra	Corn bunting	0	[3]	0	[3]	0	[3]	0	[3]
Myiopsitta monachus	Monk parakeet					0	[65]	0	[65]
Nectarinia osea	Orange-tufted sunbird	0	[1]	0	[1]	0	[1]	0	[1]
Notiomystis cincta	New Zealand hihi	0	[1]	0	[1]	0	[1]	0	[1]
Oceanodroma leucorhoa	Leach's storm-petrel					0.56	[66]	0.56	[66]
Oenanthe oenanthe	Northern wheatear	0	[3]	0	[3]	0	[3]	0	[3]
Otus elegans	Elegant scops-owl					0	[67]	0	[67]
Otus flammeolus	Flammulated owl					0	[1]	0	[1]
Pachycephala pectoralis	Golden whistlers	0	[1]	0	[1]	0.2844	[68]	0.2844	[68]
Panurus biarmicus	Bearded tit					0.55	[69]	0.55	[69]
Paradoxornis webbianus	Vinous-throated parrotbills	0.5	[70]			0.5	[70]		
Parus ater	Coal tit	0	[3]	0	[3]	0	[3]	0	[3]
Parus atricapillus	Black-capped chickadee	0	[1]	0	[1]	0	[1]	0	[1]
Parus caeruleus	Blue tit	0	[3]	0	[3]	0	[3]	0	[3]
Parus cristatus	Crested tit	0	[3]	0	[3]	0	[3]	0	[3]

Parus major	Great tit	0	[3]	0	[3]	0	[3]	0	[3]
Parus montanus	Willow tit	0	[3]	0	[3]	0	[3]	0	[3]
Parus varius	Varied tit					0	[1]	0	[1]
Passer domesticus	House sparrow					0.3221	[71]	0.3221	[71]
Passer montanus	Tree sparrow	0.5	[72]	0.5	[72]				
Passerculus sandwichensis	Savannah sparrow	0	[1]	0	[1]	0	[1]	0	[1]
Passerina caerulea	Blue grosbeak	0	[1]	0	[1]	0	[1]	0	[1]
Passerina cyanea	Indigo bunting	0	[1]	0	[1]	0	[1]	0	[1]
Perisoreus canadensis	Canada jay					0	[73]	0	[73]
Petroica australis	New Zealand robin	0	[1]	0	[1]	0	[1]	0	[1]
Petroica goodenovii	Red-capped robin	0	[1]	0	[1]	0	[1]	0	[1]
Petronia petronia	Rock sparrow	0	[3]	0	[3]	0	[3]	0	[3]
Phalaropus lobatus	Red-necked phalarope	1	[74]	1	[74]	1	[74]	1	[74]
Philesturnus carunculatus	Saddleback	0	[1]	0	[1]	0	[1]	0	[1]
Phoenicurus ochruros	Black redstart	0	[3]	0	[3]	0	[3]	0	[3]
Phoenicurus phoenicurus	Common redstart	0	[3]	0	[3]	0	[3]	0	[3]
Phylidonyris pyrrhopterus	Crescent honeyeater	0	[1]	0	[1]	0	[1]	0	[1]
Phylloscopus fuscatus	Dusky warbler	0	[75]	0	[75]	0	[75]	0	[75]
Phylloscopus sibilatrix	Wood warbler	0	[3]	0	[3]	0	[3]	0	[3]
Phylloscopus trochilus	Willow warbler	0	[3]	0	[3]	0	[3]	0	[3]
Pipilo maculatus	Spotted towhee	0	[1]	0	[1]	0	[1]	0	[1]
Piranga olivacea	Scarlet tanager	0	[1]	0	[1]	0	[1]	0	[1]
Platycercus elegans	Crimson Rosella					0	[76]	0	[76]
Plectrophenax nivalis	Snow bunting	0	[3]	0	[3]	0	[3]	0	[3]
Poecile gambeli	Mountain chickadees					0	[1]	0	[1]
Poecile palustris	Marsh Tits	0	[1]	0	[1]	0	[1]	0	[1]

Progne subis	Purple martin					0	[1]	0	[1]
Promerops cafer	Cape sugarbird	0	[1]	0	[1]	0	[1]	0	[1]
Prosthemadera novaeseelandiae	Tui					0	[1]	0	[1]
Puffinus tenuirostris	Short-tailed shearwater					0.52	[77]	0.52	[77]
Pygoscelis papua	Gentoo penguin					0.5376	[78]	0.5376	[78]
Ramphocelus costaricensis	Cherrie's tanager	0	[1]	0	[1]	0	[1]	0	[1]
Remiz coronatus	White-crowned penduline tit					0	[1]	0	[1]
Rhipidura fuliginosa	Grey fantail					0.4316	[79]	0.4316	[79]
Rissa tridactyla	Black-legged kittiwake					0.46	[80]	0.46	[80]
Sayornis phoebe	Eastern phoebe	0	[1]	0	[1]				
Serinus canaria	Canary	0	[3]	0	[3]	0	[3]	0	[3]
Serinus serinus	Serin	0	[3]	0	[3]	0	[3]	0	[3]
Setophaga ruticilla	American redstart	0	[81]	0	[81]	0	[81]	0	[81]
Sialia currucoides	Mountain bluebird	0	[82]	0	[82]	0	[82]	0	[82]
Sialia sialis	Eastern bluebird	0	[83]	0	[83]	0	[83]	0	[83]
Sicalis flaveola	Saffron finch					0	[84]	0	[84]
Sitta europaea	European nuthatch	0	[3]	0	[3]	0	[3]	0	[3]
Spiza americana	Dickcissel	0	[1]	0	[1]	0	[1]	0	[1]
Spizella pusilla	Field sparrow	0	[1]	0	[1]	0	[1]	0	[1]
Steganopus tricolor	Wilson's phalarope					1	[85]	1	[85]
Sterna hirundo	Common tern					0.4569	[86]	0.4569	[86]
Strix aluco	Tawny owl					0	[3]	0	[3]
Sturnus unicolor	Spotless starling					0	[3]	0	[3]
Sturnus vulgaris	Common starling					0	[3]	0	[3]
Sula dactylatra	Masked booby					0.5484	[87]	0.5484	[87]
Sula sula	Red-footed booby					0.5	[88,89]		

Tachycineta bicolor	Tree swallow					0	[1]	0	[1]
Tachycineta leucorrhoa	White rumped swallow					0	[90]	0	[90]
Thalassoica antarctica	Antarctic petrel					0.5851	[91]	0.5851	[91]
Thryothorus ludovicianus	Carolina wren					0	[1]	0	[1]
Thryothorus pleurostictus	Banded wren					0	[92]	0	[92]
Thryothorus rufalbus	Rufous-and-white wren					0	[93]	0	[93]
Troglodytes aedon	House wren					0	[1]	0	[1]
Troglodytes troglodytes	Eurasian wren	1	[94]	1	[94]	0	[3]	0	[3]
Turdus grayi	Clay-colored robin	0	[95]	0	[95]	0	[95]	0	[95]
Turdus migratorius	American robin	0	[96]	0	[96]	0	[96]	0	[96]
Tyrannus forficatus	Scissor-tailed flycatchers	0	[97]	0	[97]	0	[97]	0	[97]
Tyrannus tyrannus	Eastern kingbird	0	[1]	0	[1]	0	[1]	0	[1]
Tyto alba	Barn owl					0	[3]	0	[3]
Upupa epops	Eurasian hoopoe					0	[3]	0	[3]
Uria lomvia	Thick-billed murres					0.5	[98]		
Uria aalge	Common murres					0.4813	[99,100]	0.4813	[99,100]
Vermivora chrysoptera	Golden-winged warbler	0	[101]	0	[101]	0	[101]	0	[101]
Vireo olivaceus	Red-eyed vireo	0	[1]	0	[1]	0	[1]	0	[1]
Vireo solitarius	Blue-headed vireo	0.243	[102]	0.243	[102]	0.491	[102]	0.491	[102]
Volatinia jacarina	Blue-black grassquits					0	[103]	0	[103]
Wilsonia citrina	Hooded warbler	0	[104]	0	[104]	0	[104]	0	[104]
Zonotrichia albicollis	White-throated sparrow	0	[1]	0	[1]	0	[1]	0	[1]
Zonotrichia capensis	Rufous-collared sparrow	0	[105]	0	[105]	0	[105]	0	[105]
Zonotrichia leucophrys	White-crowned sparrow	0	[1]	0	[1]	0	[1]	0	[1]
Zosterops lateralis	Capricorn silvereye	0	[106]	0	[106]				

1. Del Hoyo J, Del Hoyo J, Elliott A, Sargatal J. 1992 *Handbook of the birds of the world*. Lynx edicions Barcelona.

2. Rosenfield RN, Bielefeldt J, Cary J. 1991 Copulatory and other pre-incubation behaviors of Cooper's Hawks. *Wilson Bull.* **103**, 656–660.

3. Cramp S. 1977 *The Birds of the Western Palearctic*. Oxford: Oxford Univ. Press.

4. Hamao S, Saito DS. 2005 Extrapair fertilization in the black-browed reed warbler (Acrocephalus bistrigiceps): effects on mating status and nesting cycle of cuckolded and cuckolder males. *The Auk* **122**, 1086–1096.

5. Dowsett-Lemaire F. 1981 Eco-ethological aspects of breeding in the Marsh Warbler, Acrocephalus palustris. *Rev. Ecol. Terre Vie* **35**, 437–492.

6. Klimczuk E, Halupka L, Czyż B, Borowiec M, Nowakowski JJ, Sztwiertnia H. 2015 Factors driving variation in biparental incubation behaviour in the reed warbler Acrocephalus scirpaceus. *Ardea* **103**, 51–59.

7. Hoi H, Fessl B, Kleindorfer S. 1995 More is not always better: male incubation in two Acrocephalus warblers. *Behaviour* **132**, 607–625.

8. Brown P, Davies MG. 1949 *Reed-Warblers: an introduction to their breeding-biology and behaviour*. Foy Publications.

9. JÖNSSON PE, ALERSTAM T. 1990 The adaptive significance of parental role division and sexual size dimorphism in breeding shorebirds. *Biol. J. Linn. Soc.* **41**, 301–314.

10. Wojczulanis-Jakubas K, Jakubas D, Stempniewicz L. 2009 Sex-specific parental care by incubating little auks (Alle alle). *Ornis Fenn* **86**, 140–148.

11. Stempniewicz L, Jezierski J. 1987 Incubation shifts and chick feeding rate in the Little Auk Alle alle in Svalbard. *Ornis Scand.* , 152–155.

12. Harrison HH. 1979 *A Field Guide to Western Birds' Nests: Of 520 Species Found Breeding in the United States West of the Mississippi River*. Houghton Mifflin Harcourt.

13. Margalida A, González LM, Sánchez R, Oria J, Prada L. 2007 Parental behaviour of Spanish Imperial Eagles Aquila adalberti: sexual differences in a moderately dimorphic raptor. *Bird Study* **54**, 112–119.

14. Wiley JW, Wiley BN. 1981 Breeding season ecology and behavior of Ridgway's Hawk (Buteo ridgwayi). *The Condor* **83**, 132–151.

15. Fitzner RE. 1980 Behavioral ecology of the Swainson's Hawk (Buteo swainsoni) in Washington.

16. In press. Breeding - Purple Sandpiper - Calidris maritima - Birds of the World. See https://birdsoftheworld.org/bow/species/pursan/cur/breeding (accessed on 2 September 2022).

17. Pierce EP. 1997 Sex roles in the monogamous Purple Sandpiper Calidris maritima in Svalbard. *Ibis* **139**, 159–169.

18. Granadeiro JP, Burns MD, Furness RW. 1998 Patterns of activity and burrow attendance in Cory's Shearwater Calonectris diomedea as revealed by a novel logging technique. *Ibis* **140**, 458–466.

19. Young EC. 1963 The breeding behaviour of the south polar skua Catharacta maccormicki. *Ibis* **105**, 203–233.

20. Maurer G. 2008 Who cares? Males provide most parental care in a monogamous nesting cuckoo. *Ethology* **114**, 540–547.

21. Skutch AF. 1960 *Life histories of Central American birds. 2, Families Vireonidae, Sylviidae, Turdidae, Troglodytidae, Paridae, Corvidae, Hirundinidae and Tyrannidae*. Cooper Ornithological Society.

22. AlRashidi M. 2016 Breeding biology of the Kentish Plover Charadrius alexandrinus in the Sabkhat Al-Fasl Lagoons, Saudi Arabia (Aves: Charadriiformes). *Zool. Middle East* **62**, 105–111.

23. St Clair JJ, Herrmann P, Woods RW, Székely T. 2010 Female-biased incubation and strong diel sex-roles in the Two-banded Plover Charadrius falklandicus. *J. Ornithol.* **151**, 811–816.

24. Wallander J. 2003 Sex roles during incubation in the Common Ringed Plover. *The Condor* **105**, 378–381.

25. St Clair JJ, Kuepper C, Herrmann P, Woods RW, Szekely T. 2010 Unusual incubation sex-roles in the Rufous-chested Dotterel Charadrius modestus. *Ibis* **152**, 402–404.

26. Blanken MS, Nol E. 1998 Factors affecting parental behavior in Semipalmated Plovers. *The Auk* **115**, 166–174.

27. Jónsson JE, Ryder JP, Alisauskas RT. 2020 Ross's Goose (Anser rossii), version 1.0. *Birds World* (doi:10.2173/bow.rosgoo.01)

28. Swift JJ. 1960 Notes on the behaviour of Whiskered Terns. *Br Birds* **53**, 557–572.

29. In press. Breeding - Grass Wren - Cistothorus platensis - Birds of the World. See https://birdsoftheworld.org/bow/species/sedwre/cur/breeding (accessed on 2 September 2022).

30. Burns JT. 1982 Nests, territories, and reproduction of Sedge Wrens (Cistothorus platensis). *Wilson Bull.* , 338–349.

31. Stewart PA. 1974 A nesting of Black Vultures. *The Auk* **91**, 595–600.

32. Masello JF, Quillfeldt P. 2004 Are haematological parameters related to body condition, ornamentation and breeding success in wild burrowing parrots Cyanoliseus patagonus? *J. Avian Biol.* **35**, 445–454.

33. Taborsky M, Brugger C. 1994 The functional significance of shared incubation. *J. Für Ornithol.* **135**, 273.

34. Whittingham LA, Lifjeld JT. 1995 High paternal investment in unrelated young: extra-pair paternity and male parental care in house martins. *Behav. Ecol. Sociobiol.* **37**, 103–108.

35. Wiktander U, Olsson O, Nilsson SG. 2000 Parental care and social mating system in the Lesser Spotted Woodpecker Dendrocopos minor. *J. Avian Biol.* **31**, 447–456.

36. Harding KC. 1931 Nesting habits of the black throated blue warbler. *The Auk* , 512–522.

37. Lawrence LDK. 1948 Comparative study of the nesting behavior of Chestnut-sided and Nashville warblers. *The Auk* , 204–219.

38. Schrantz FG. 1943 Nest life of the eastern yellow warbler. *The Auk* , 367–387.

39. Croxall JP, Ricketts C. 1983 Energy costs of incubation in the Wandering Albatross Diomedea exulans. *Ibis* **125**, 33–39.

40. Stutchbury BJ, Morton ES, Woolfenden B. 2007 Comparison of the mating systems and breeding behavior of a resident and a migratory tropical flycatcher. *J. Field Ornithol.* **78**, 40–49.

41. Walkinshaw LH. 1966 Summer observations of the Least Flycatcher in Michigan. *Jack-Pine Warbler* **44**, ll.

42. McCabe RA. 1993 *The little green bird: ecology of the willow flycatcher*. Rusty Rock Press, Department of Wildlife Ecology [University of Wisconsin].

43. Allen MC, Napoli MM, Sheehan J, Master TL, Pyle P, Whitehead DR, Taylor T. 2017 Acadian Flycatcher (Empidonax virescens). *Birds N. Am. PG Rodewald Ed. Cornell Lab Ornithol. Ithaca NY Version* **2**.

44. Craig AJF. 2020 Southern Red Bishop (Euplectes orix), version 1.0. *Birds World* (doi:10.2173/bow.redbis.01)

45. Sodhi NS, James PC, Warkentin IG, Oliphant LW. 1992 Breeding ecology of urban Merlins (Falco columbarius). *Can. J. Zool.* **70**, 1477–1483.

46. Razafimanjato G, de Roland L-AR, Rabearivony J, Thorstrom R. 2007 Nesting biology and food habits of the Peregrine Falcon Falco peregrinus radama in the south-west and central plateau of Madagascar. *Ostrich-J. Afr. Ornithol.* **78**, 7–12.

47. Deng, Qiu-Xiang, Deng, Wen-Hong, Gao, Wei. 2010 Breeding biology of the yellow-rumped flycatcher Ficedula zanthopygia in northeast China. *Ardeola* **57**, 103–110.

48. Clement P, de Juana E. 2020 Yellow-rumped Flycatcher (Ficedula zanthopygia), version 1.0. *Birds World* (doi:10.2173/bow.korfly1.01)

49. Hatch SA. 1990 Incubation rhythm in the fulmar Fulmarus glacialis: annual variation and sex roles. *Ibis* **132**, 515–524.

50. Mallory ML. 2009 Incubation scheduling by northern fulmars (Fulmarus glacialis) in the Canadian High Arctic. *J. Ornithol.* **150**, 175–181.

51. Gostomski TJ, Evers DC. 1998 Time-activity budget for common loons, Gavia immer, nesting on Lake Superior. *Can. Field-Nat.* **112**, 191–197.

52. Goodale W, Attix L, Evers D. 2005 Common Loon, Gavia immer, nest attendance patterns recorded by remote video camera. *Can. Field-Nat.* **119**, 455–456.

53. Stewart RE. 1953 A life history study of the Yellow-throat. *Wilson Bull.* **65**, 99–115.

54. Duan W, Bookhout TA. 1997 Breeding Behavior of Isolation-Reared Sandhill Cranes (Comportamiento Reproductivo de Grus canadensis tabida Criadas en Aislamiento). *J. Field Ornithol.* , 200–207.

55. Ens BJ, Kruijt JP, Jenkins L, Burke T, Heg D. 1993 Why does the typically monogamous oystercatcher (Haematopus ostralegus) engage in extra-pair copulations? *Behaviour* **126**, 247–289.

56. Magrath MJ, Elgar MA. 1997 Paternal care declines with increased opportunity for extra–pair matings in fairy martins. *Proc. R. Soc. Lond. B Biol. Sci.* **264**, 1731–1736.

57. Evans M, Gow E, Roth RR, Johnson MS, Underwood TJ, Poole AF. 2011 Wood thrush (Hylocichla mustelina), version 2.0. *Birds N. Am. Cornell Lab Ornithol. Ithaca N. Y. USA Httpsdoi Org102173bna* **246**.

58. Carboneras C, Kirwan GM. 2020 Blue Duck (Hymenolaimus malacorhynchos), version 1.0. *Birds World* (doi:10.2173/bow.bluduc1.01)

59. In press. Yellow-breasted Chat - Icteria virens - Birds of the World. See https://birdsoftheworld.org/bow/species/yebcha/cur/introduction (accessed on 8 September 2022).

60. Bent AC. 1958 Life histories of North American blackbirds, orioles, tanagers, and allies. *Bull. U. S. Natl. Mus.*

61. Giesen KM, Braun CE. 1979 Nesting behavior of female White-tailed Ptarmigan in Colorado. *The Condor* **81**, 215–217.

62. Yosef R, International Shrike Working Group I. 2020 Bull-headed Shrike (Lanius bucephalus), version 1.0. *Birds World* (doi:10.2173/bow.buhshr1.01)

63. Pierotti R. 1981 Male and female parental roles in the Western Gull under different environmental conditions. *The Auk* **98**, 532–549.

64. Laskemoen T, Fossøy F, Rudolfsen G, Lifjeld JT. 2008 Age-related variation in primary sexual characters in a passerine with male age-related fertilization success, the bluethroat Luscinia svecica. *J. Avian Biol.* **39**, 322–328.

65. Eberhard JR. 1998 Breeding biology of the Monk Parakeet. *Wilson Bull.* , 463–473.

66. Mauck RA, Zangmeister JL, Cerchiara JC, Huntington CE, Haussmann MF. 2011 Male-biased reproductive effort in a long-lived seabird. *Evol. Ecol. Res.* **13**, 19–33.

67. Murakami R, Sawada A, Ono H, Takagi M. 2022 The effect of experience on parental role division in Ryukyu Scops Owl Otus elegans. *Ornithol. Sci.* **21**, 35–44.

68. van Dongen WF, Yocom LL. 2005 Breeding biology of a migratory Australian passerine, the golden whistler (Pachycephala pectoralis). *Aust. J. Zool.* **53**, 213–220.

69. Hoi H, Hoi-Leitner M. 1997 An alternative route to coloniality in the bearded tit: females pursue extra-pair fertilizations. *Behav. Ecol.* **8**, 113–119.

70. Kim C-H. 1998 Social behavior of the crow tit Paradoxornis webbiana during the breeding season. 한국조류학회지 **5**, 17–26.

71. Bartlett TL, Mock DW, Schwagmeyer PL. 2005 Division of labor: incubation and biparental care in house sparrows (Passer domesticus). *The Auk* **122**, 835–842.

72. In press. Eurasian Tree Sparrow - Passer montanus - Birds of the World. See https://birdsoftheworld.org/bow/species/eutspa/cur/introduction (accessed on 8 September 2022).

73. Strickland D, Ouellet H. 1993 *Gray Jay: Perisoreus Canadensis*. American Ornithologists' Union.

74. Rubega MA, Schamel D, Tracy DM. 2020 Red-necked Phalarope (Phalaropus lobatus), version 1.0. *Birds World* (doi:10.2173/bow.renpha.01)

75. Forstmeier W, Kuijper DPJ, Leisler B. 2001 Polygyny in the dusky warbler, Phylloscopus fuscatus: the importance of female qualities. *Anim. Behav.* **62**, 1097–1108. (doi:10.1006/anbe.2001.1859)

76. Lenz M. In press. Crimson Rosellas 'Platycercus elegans' nesting in buildings in Canberra. *Aust. Bird Watch.* **12**, 171–173. (doi:10.3316/informit.600540682278238)

77. Carey MJ. 2011 Incubation routine, duration of foraging trips and regulation of body mass in Short-tailed Shearwaters (Ardenna tenuirostris). *Emu - Austral Ornithol.* **111**, 166–171. (doi:10.1071/MU10043)

78. Williams TD, Rothery P. 1990 Factors Affecting Variation in Foraging and Activity Patterns of Gentoo Penguins (Pygoscelis papua) During the Breeding Season at Bird Island, South Georgia. *J. Appl. Ecol.* **27**, 1042–1054. (doi:10.2307/2404394)

79. Powlesland MH. 1982 A breeding study of the South Island fantail (Rhipidura fuliginosa fuliginosa). *Notornis* **29**, 181–195.

80. Coulson JC, Wooller RD. 1984 Incubation under natural conditions in the kittiwake gull, Rissa tridactyla. *Anim. Behav.* **32**, 1204–1215.

81. Sturm L. 1945 A study of the nesting activities of the American Redstart. *The Auk* , 189–206.

82. Criddle N. 1927 Habits of the mountain bluebird in Manitoba. *Can. Field-Nat.* **41**, 40–44.

83. In press. Breeding - Eastern Bluebird - Sialia sialis - Birds of the World. See https://birdsoftheworld.org/bow/species/easblu/cur/breeding (accessed on 8 September 2022).

84. Burnham H, Cruz-Bernate L. 2020 Parental investment does not directly affect reproductive success in the saffron finch. *J. Avian Biol.* **51**.

85. In press. Breeding - Wilson's Phalarope - Phalaropus tricolor - Birds of the World. See https://birdsoftheworld.org/bow/species/wilpha/cur/breeding (accessed on 8 September 2022).

86. Wiggins DA, Morris RD. 1987 Parental care of the Common Tern Sterna hirundo. *Ibis* **129**, 533–540.

87. Nelson JB. 1967 The breeding behaviour of the White Booby Sula dactylatra. *Ibis* **109**, 194–231.

88. Nelson S, Nelson B. 1978 *The Sulidae: gannets and boobies*. Oxford University Press, USA.

89. Chastel O, Lormée H. 2002 Patterns of prolactin secretion in relation to incubation failure in a tropical seabird, the red-footed booby. *The Condor* **104**, 873–876.

90. Wischhoff U, Marques-Santos F, Ardia DR, Roper JJ. 2015 White-rumped swallows prospect while they are actively nesting. *J. Ethol.* **33**, 145–150.

91. Lorentsen S-H, Røv N. 1995 Incubation and brooding performance of the Antarctic petrel Thalassoica antarctica at Svarthamaren, Dronning Maud Land. *Ibis* **137**, 345–351.

92. Vehrencamp SL, Hall ML, Bohman ER, Depeine CD, Dalziell AH. 2007 Song matching, overlapping, and switching in the banded wren: the sender's perspective. *Behav. Ecol.* **18**, 849–859.

93. Douglas SB, Heath DD, Mennill DJ. 2012 Low levels of extra-pair paternity in a neotropical duetting songbird, the rufous-and-white wren (Thryothorus rufalbus). *The Condor* **114**, 393–400.

94. Kroodsma DE, Brewer D, Christie D, Marks JS. 2020 Eurasian Wren (Troglodytes troglodytes), version 1.0. *Birds World* (doi:10.2173/bow.winwre4.01)

95. In press. Breeding - Clay-colored Thrush - Turdus grayi - Birds of the World. See https://birdsoftheworld.org/bow/species/clcrob/cur/breeding (accessed on 8 September 2022).

96. In press. Breeding - American Robin - Turdus migratorius - Birds of the World. See https://birdsoftheworld.org/bow/species/amerob/cur/breeding (accessed on 8 September 2022).

97. Fitch Jr FW. 1950 Life history and ecology of the Scissor-tailed Flycatcher, Muscivora forficata. *The Auk* **67**, 145–168.

98. Paredes R, Boness DJ, Jones IL. 2006 Parental roles of male and female thick-billed murres and razorbills at the Gannet Islands, Labrador. *Behaviour* **143**, 451–481.

99. Verspoor E, Birkhead TR, Nettleship DN. 1987 Incubation and brooding shift duration in the Common Murre, Uria aalge. *Can. J. Zool.* **65**, 247–252.

100. Wanless S, Harris MP. 1986 Time spent at the colony by male and female guillemots Uria aalge and razorbills Alca torda. *Bird Study* **33**, 168–176.

101. In press. Breeding - Golden-winged Warbler - Vermivora chrysoptera - Birds of the World. See https://birdsoftheworld.org/bow/species/gowwar/cur/breeding (accessed on 8 September 2022).

102. Morton ES, Stutchbury BJ, Howlett JS, Piper WH. 1998 Genetic monogamy in blue-headed vireos and a comparison with a sympatric vireo with extrapair paternity. *Behav. Ecol.* **9**, 515–524.

103. Caetano JV, Maia MR, Manica LT, Macedo RH. 2014 Immune-related effects from predation risk in Neotropical blue-black grassquits (Volatinia jacarina). *Behav. Processes* **109**, 58–63.

104. Niven DK. 1993 Male-male nesting behavior in Hooded Warblers. *Wilson Bull.* **105**, 190–193.

105. Miller AH, Miller VD. 1968 The behavioral ecology and breeding biology of the Andean sparrow, Zonotrichia capensis. *Caldasia* , 83–154.

106. Barnett CA, Briskie JV. 2010 Silvereyes Zosterops lateralis increase incubation attentiveness in response to increased food availability. *Ibis* **152**, 169–172.
